# A Synthetic Lectin for Glucuronate

**DOI:** 10.1021/acscentsci.5c00951

**Published:** 2025-08-01

**Authors:** Canjia Zhai, Chengkai Xu, Yunpeng Cui, Lukasz Wojtas, Jianfeng Cai, Wenqi Liu

**Affiliations:** Department of Chemistry, 7831 University of South Florida, 4202 E. Fowler Ave, Tampa, Florida 33620, United States

## Abstract

The selective recognition of hydrophilic carbohydrates in water remains a longstanding challenge in supramolecular chemistry due to solvent competition and the lack of a strong driving force comparable to the hydrophobic effect. Herein, we report the design, synthesis, and characterization of a water-soluble tetralactam macrocycle, MPNT^2+^·2Cl^–^, as a highly effective synthetic lectin for glucuronate. MPNT^2+^·2Cl^–^ features two dimethylnaphthalene panels, pyridinium spacers, and morpholine side chains, forming a rigid, preorganized cavity with convergent hydrogen bond donors, polarized C–H donors, and complementary electrostatic interactions. The receptor achieves a binding affinity of 103,000 M^–1^ for glucuronate in water, over 19-fold higher than previous synthetic systems, along with excellent selectivity over structurally similar carbohydrates. Single-crystal X-ray analysis, DFT calculation, and IGMH analysis reveal a dense network of [N–H···O], [C–H···O], and [C–H···π] interactions, highlighting the role of stereoelectronic complementarity in complex formation. Moreover, MPNT^2+^·2Cl^–^ acts as a chiroptical sensor, producing binding-induced circular dichroism signals that enable sensitive detection of glucuronic acid at physiologically relevant concentrations. This work presents a generalizable strategy for designing synthetic lectins that recognize carbohydrates in aqueous solutions and opens up new possibilities for developing molecular sensors and diagnostic tools for biologically important anionic sugars.

## Graphical abstract

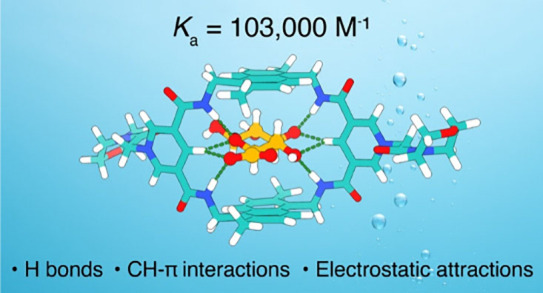

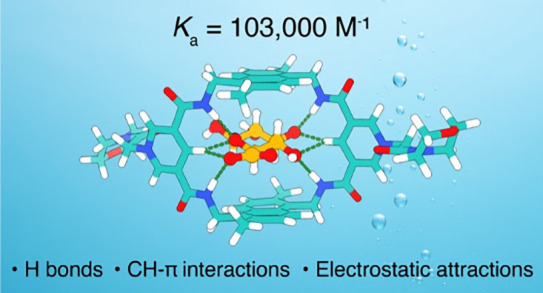

## Introduction

Glucuronic acid is an anionic sugar that is critical to human health and disease.^1,2^ Owing to its highly hydrophilic nature, it serves as a key solubilizing agent in glucuronidation, a major pathway for detoxifying and eliminating a wide range of endogenous and exogenous compounds. Abnormal levels of glucuronic acid often signal disruptions in detoxification and metabolic processes, and it is increasingly recognized as a valuable biomarker for toxin exposure, liver dysfunction, and certain metabolic disorders.^3–5^ Developing synthetic lectins that selectively bind and report glucuronic acid holds significant promise for advancing disease diagnostics and enabling new therapeutic strategies.^6–9^

One of the longstanding challenges in supramolecular chemistry is the development of synthetic receptors capable of effectively and selectively binding hydrophilic substrates in water.^10–13^ Conventional receptors rely^14–20^ on noncovalent interactions such as hydrogen bonding and electrostatic forces, which, while highly effective in organic solvents, are dramatically weakened or even disrupted in water, a polar medium that competes as both a hydrogen bond donor and acceptor.^21,22^ Moreover, the strong solvation of both hydrophilic substrates and the polar functional groups of receptors imposes a substantial desolvation penalty that must be overcome to achieve effective binding. Unlike the well-established hydrophobic effect that facilitates the recognition of nonpolar substrates, no comparable driving force exists for the recognition of hydrophilic targets in water.^23–25^ Carbohydrates and hydrophilic anions, in particular, are notoriously challenging to bind in water using synthetic receptors.^26–32^ Glucuronate, which uniquely integrates both sugar and anionic character within a small molecular framework, exemplifies a formidable challenge for selective recognition in water.

Lectins are a class of proteins that nature employs to achieve selective carbohydrate binding. These biological receptors address the challenge of recognizing hydrophilic substrates by forming well-defined binding pockets with an array of functional groups that bind substrates through multiple noncovalent interactions.^33^ A representative example is the carbohydrate-binding module CBM35 (Figure 1a), which features^34^ a tryptophan aromatic surface for [C–H···π] interactions, a guanidinium side chain to form salt bridges with carboxylate, a calcium ion to coordinate with carboxylate, and tyrosine and asparagine residues that establish a network of hydrogen bonds. Designing synthetic analogues of lectins remains a formidable challenge, as small-molecule receptors have limited scaffolds to replicate such sophisticated functional group arrangements. Furthermore, synthetic receptors are typically constructed from hydrophobic organic building blocks, leading to poor water solubility. Incorporating water-solubilizing groups without disrupting essential binding motifs requires meticulous molecular design.

**Figure 1:**
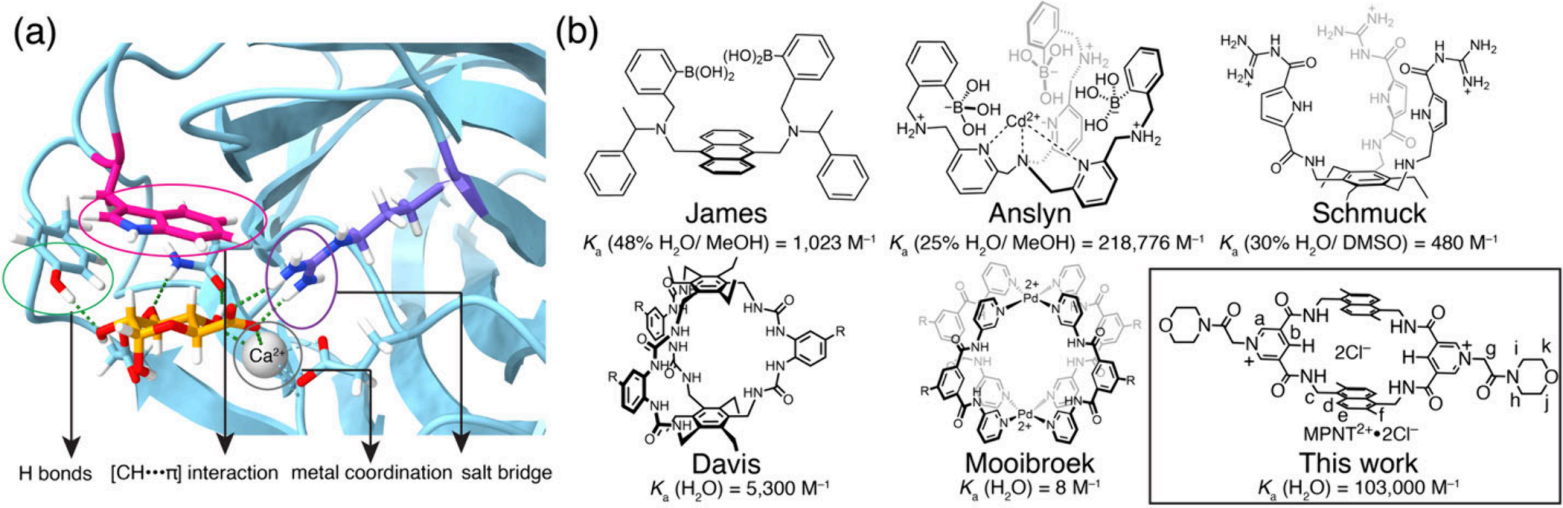
(a) Single-crystal structure (PDB ID: 4QB2) of glucuronate bound to CBM35. (b) Comparison of the binding performance of current synthetic receptors for glucuronic acid.

As a result, early synthetic receptors targeting glucuronic acid were mainly studied in organic solvents containing less than 50% water. Notable examples include James’s boronic acid receptors, which bind^35^ cis-diols of carbohydrates by forming boronic esters while employing anthracene panels for [C–H···π] interactions; Anslyn’s receptors, which combine^36^ boronic acid motifs with Cd^2+^ coordination sites to bind anionic carboxylated and phosphorylated sugars; and Schmuck’s guanidinium-based systems, which use^37^ charge-assisted hydrogen bonding to capture anionic sugars. While these systems exhibited promising binding affinities, they generally showed poor selectivity toward glucuronic acid over structurally similar carbohydrates.

More recently, synthetic receptors operating in fully aqueous media have emerged. Davis’s hexaurea temple combines^38^ [C–H···π] interactions with hydrogen bonding, while Mooibroek’s highly charged receptor (net charge +16) employs^39^ a convergent hydrogen-bonding pocket to bind glucuronate through charge-assisted interactions. Despite these promising developments, existing water-compatible receptors struggle with modest binding affinities and lack selectivity against structurally similar carbohydrates. Compounding this challenge is the lack of single-crystal structures of synthetic receptor–carbohydrate complexes, which limits detailed insights into their binding mechanisms in water. At a fundamental level, the field continues to face the unmet challenge of establishing general molecular design principles for the selective and effective recognition of anionic carbohydrates like glucuronate in water.

Our group has developed a dynamic approach to designing hydrogen-bonding receptors to address^40–42^ the longstanding challenges of carbohydrate and anion recognition in water. These receptors incorporate pyridinium spacers, which enhance water solubility and introduce polarized C–H bonds at the para position of the pyridinium ring, serving as additional hydrogen bond donors. This strategy has delivered good performance in recognizing highly hydrophilic substrates such as oxalate, sulfate, and glucose. To further improve glucose binding affinity in water, we replaced^40^ the durene units with anthracene panels. The expanded aromatic surface enhanced glucose recognition at low concentrations by promoting [C–H···π] interactions. However, while increasing aromatic surface area appears to be an intuitive strategy for boosting substrate binding, we observed that the large aromatic surfaces of anthracene induce receptor aggregation at higher concentrations, leading to a dramatic loss in binding affinity. Similar phenomena have been reported by the Davis group, where the enlargement of aromatic panels caused^43,44^ the collapse of the binding cavity. These findings underscore a fundamental challenge in receptor design: simply increasing aromatic surface area is not a general solution for improving substrate binding. Instead, there is a critical need to identify the sweet spot where the aromatic panels provide enough surface area for stabilizing interactions without causing receptor aggregation or cavity collapse.

Herein, we report the design and synthesis of a tetralactam macrocycle, MPNT^2+^·2Cl^–^, featuring two parallel 2,6-dimethylnaphthalene panels. The methyl groups on the naphthalene impose^45^ steric hindrance on the methylene protons, effectively preventing cavity collapse and creating a rigid, preorganized binding pocket with four convergent hydrogen bond donors. To enhance water solubility, we employed chloroacetylmorpholine as an alkylating agent, addressing the aggregation issues encountered in our previous work. Additionally, the two pyridinium spacers not only contribute polarized C–H bonds as hydrogen bond donors but also introduce proximal positive charges around the binding pocket, facilitating glucuronate binding through complementary electrostatic attractions. As a result, we successfully integrated [C–H···π] interactions, electrostatic attractions, and a robust hydrogen-bonding network within a small but compact synthetic receptor, achieving a binding affinity of 103,000 M^–1^—more than 19-fold stronger than the best previously reported receptor^38^ for this highly challenging hydrophilic target in water. The receptor also exhibits good selectivity for glucuronate over structurally similar carbohydrates. Furthermore, single-crystal X-ray analysis of the glucuronate⊂MPNT^2+^ complex provided direct structural evidence of the multiple noncovalent interactions responsible for its high affinity. We also demonstrated the receptor’s utility as a chiroptical sensor capable of reporting physiological concentrations of glucuronic acid in water through binding-induced circular dichroism signals. This work not only establishes a powerful platform for selective carbohydrate recognition in aqueous media but also opens new avenues for developing synthetic sensors and diagnostic tools targeting biologically important hydrophilic substrates.

## Results and Discussion

### Synthesis

Conventionally, tetralactam macrocycles have been synthesized^46–48^ using a statistical high-dilution approach, in which bisacyl chlorides react with bisamines to generate the target macrocycle alongside larger structural analogues and linear oligomers. This method typically suffers from low yields and presents significant challenges in product separation and purification. To overcome these limitations, we adopted a dynamic covalent approach pioneered by Mastalerz,^49,50^ Andrews,^51–53^ Badjić^54^ and our group.^40–42^ By simply mixing Bisamine 1 with bisaldehyde 2, we achieve (Scheme 1) the quantitative formation of the [2 + 2] imine macrocycle, capitalizing on the reversible nature of imine chemistry. This dynamic system allows^55–57^ error-checking and self-correction, ultimately funneling the reaction toward imine macrocycle 3 as the thermodynamically favored product. The resulting imine macrocycle can then be efficiently converted into the corresponding amide macrocycle 4 via Pinnick oxidation, achieving a yield of 68%. Notably, this approach allows for rapid isolation of the macrocycle through simple solvent washes, eliminating the need for column chromatography. As a final step, we directly alkylated the pyridine spacers using chloroacetylmorpholine, affording the water-soluble receptor MPNT^2+^·2Cl^–^ in a good yield of 60%. Overall, this versatile and efficient synthetic strategy provides a robust general platform for constructing hydrogen-bonding macrocycles and cages, significantly expanding opportunities for developing new hydrogen-bonding systems.

**Scheme 1:**
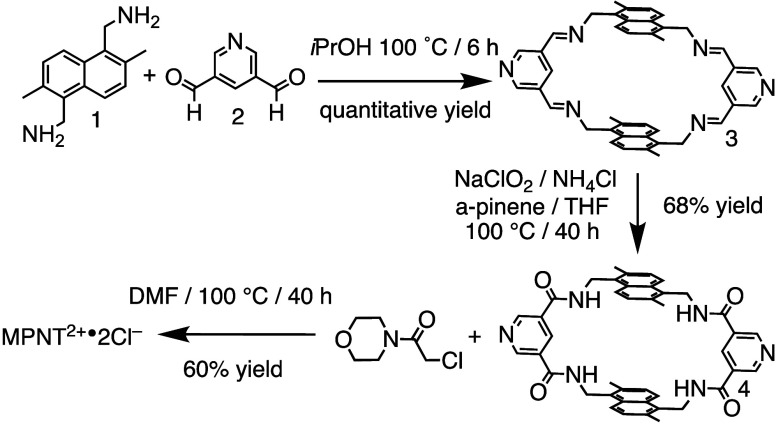
Synthesis of MPNT^2+^·2Cl^–^

### Evaluation of Carbohydrate Binding in Water

To assess the aggregation behavior of MPNT^2+^·2Cl^–^, we conducted (Figure S15) dilution experiments by recording the ^1^H NMR spectra of MPNT^2+^·2Cl^–^ in D_2_O over a concentration range of 0.015 to 0.45 mM. Across this range, no significant changes were observed in the chemical shifts of the macrocycle, indicating that MPNT^2+^·2Cl^–^ does not undergo aggregation in aqueous solution. These findings demonstrate that our design strategy—incorporating highly water-soluble morpholine side chains—effectively resolves the aggregation issues encountered in our previously reported systems.^40^

The binding affinities between MPNT^2+^·2Cl^–^ and various carbohydrates were evaluated using standard ^1^H NMR titration experiments. Initial titrations of MPNT^2+^·2Cl^–^ with glucuronic acid in D_2_O resulted in significant peak broadening of the inward-facing proton *b*, making it difficult to track (Figure S17) its chemical shift changes. To address this issue, we performed titration experiments in a mixed solvent system containing 98% H_2_O and 2% D_2_O, which allowed for observing the NH resonances and their chemical shift changes upon glucuronic acid addition. Under these conditions, titration with glucuronic acid produced (Figure 2a) a pronounced downfield shift (Δδ_b_ = +0.78 ppm) for proton *b*, accompanied by peak broadening. Concurrently, the NH protons exhibited an upfield shift (Δδ_NH_ = −0.11 ppm) with similar broadening. Additionally, protons *d* and *e* on the naphthalene panel showed slight upfield shifts (Δδ_d_ = −0.06 ppm; Δδ_e_ = −0.02 ppm). These observations strongly support the binding of glucuronate within the receptor’s cavity through a network of hydrogen bonding and [C–H···π] interactions. The displacement of cavity water molecules by the glucuronate substrate alters the local environment of the receptor’s protons, leading to the observed chemical shift changes.

**Figure 2:**
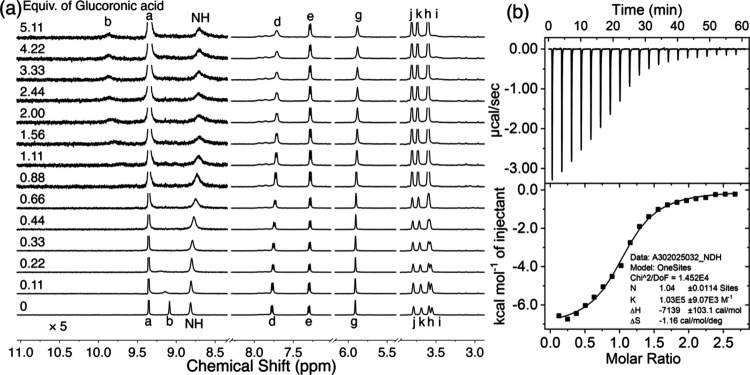
(a) Partial ^1^H NMR spectra of MPNT^2+^·2Cl^–^ (600 MHz, 98% H_2_O+2% D_2_O, 0.45 mM) titrated with glucuronic acid. The left portion of the spectra was partially zoomed in 5 times to track the change of NH and b protons. (b) ITC profile of MPNT^2+^·2Cl^–^ (0.15 mM) titrated with glucuronic acid in water at 23 °C.

The binding between MPNT^2+^·2Cl^–^ and glucuronate was further confirmed by a ^1^H–^1^H Nuclear Overhauser Effect Spectroscopy (NOESY) experiment, which revealed (Figure S14) cross-peaks indicating through-space correlations between the axial C–H protons of glucuronate and protons d and e on the naphthalene panel. These observations provide compelling evidence for the presence of [C–H···π] interactions. This result is further corroborated (Figure S12) by the upfield shift of the C–H protons of the glucuronate in the presence of MPNT^2+^·2Cl^–^. High-resolution mass spectrometry (HRMS) provided further confirmation, revealing a peak at *m*/*z* 570.2392 that closely matched the theoretical *m*/*z* of 570.2397 for the glucuronic acid⊂MPNT^2+^ adduct (Figure S5). This peak corresponds to the molecular formula [C_60_H_68_N_8_O_15_]^2+^, providing definitive evidence for the stoichiometry and formation of the complex.

By monitoring the chemical shift changes of the NH peaks upon incremental addition of glucuronic acid, we found that the binding affinity was too high to be reliably determined by NMR titration, indicating exceptionally strong binding beyond the measurable range of this technique. To overcome this limitation, we employed isothermal titration calorimetry (ITC), a more sensitive and quantitative method, to determine the binding constant between MPNT^2+^·2Cl^–^ and glucuronate. The ITC isotherm revealed exothermic binding peaks, consistent with an enthalpically favorable process. Fitting of the ITC data yielded (Figure 2b) a binding constant of 103,000 M^–1^ and a 1:1 binding stoichiometry. Notably, this affinity is over 19-fold higher than that reported by the Davis group for the best-known receptor^38^ targeting glucuronic acid, a particularly challenging hydrophilic substrate in water. The ITC analysis revealed that the binding is predominantly enthalpy-driven (Δ*H* = −7.1 kcal/mol) with only a minor entropic penalty (*T*Δ*S* = −0.3 kcal/mol). These results suggest that multiple noncovalent interactions—including hydrogen bonding, electrostatic attraction, and [C–H···π] interactions—effectively compensate for the desolvation penalty and provide substantial binding enthalpy to drive complex formation. Additionally, the release of surface-bound water molecules from both MPNT^2+^·2Cl^–^ and glucuronate into the bulk solution likely helps offset the loss of conformational and translational freedom of motion upon complexation, as reflected by the minimal entropy cost. Conformational analysis of the cavity water molecules suggests that no high-energy water exists,^58–60^ indicating that displacement of cavity water is unlikely to provide a significant enthalpic driving force for guest binding in this system.

Given the low p*K*_a_ of glucuronic acid (∼3.2), the binding affinity measured under our experimental conditions is expected to primarily reflect interaction with its deprotonated form, glucuronate. To further support this conclusion, we conducted (Figure S41) an ITC experiment using sodium glucuronate, which yielded a comparable binding constant of 6.8 × 10^4^ M^–1^. Additionally, we performed (Figure S42) a titration using glucuronic acid in a buffered solution at pH 6.5, where it exists predominantly in its ionized form. Under these conditions, a slightly lower binding affinity of 4.3 × 10^4^ M^–1^ was observed, likely due to minor competitive effects from buffer components. Despite this modest reduction, the overall consistency in binding affinities strongly supports that the high-affinity interaction observed in our system arises from the binding of glucuronate to MPNT^2+^.

The binding affinities between MPNT^2+^·2Cl^–^ and various carbohydrates (Scheme 2) were determined using ^1^H NMR and ITC experiments. Overall, the two independent methods yielded consistent binding constants (Table 1), supporting the reliability of our data. Glucose, a neutral structural analogue of glucuronic acid, exhibited a binding constant of 1,700 M^–1^ with MPNT^2+^·2Cl^–^ as measured by ITC, consistent with our previously reported systems and in line with the expected trend. As the size of the aromatic panels increases from durene to naphthalene to anthracene, the corresponding tetralactam macrocycles show progressively stronger affinities, with binding constants of 684 M^–1^, 1,700 M^–1^, and 2,410 M^–1^, respectively.^40,42^ Although MPNT^2+^·2Cl^–^ exhibits slightly lower affinity for glucose compared to its anthracene analogue, it provides a practical advantage by avoiding aggregation. ITC measurements revealed that glucose binding to MPNT^2+^·2Cl^–^ is driven predominantly by favorable enthalpy changes (Δ*H* = −4.4 kcal/mol) with minimal entropy penalty (*T*Δ*S* = −0.4 kcal/mol). These thermodynamic parameters indicate that the combined effects of hydrogen bonding and [C–H···π] interactions are sufficient to offset the desolvation penalty associated with glucose binding. Furthermore, the entropic gain from the release of surface-bound water on glucose nearly compensates for the conformational entropy loss upon binding, making MPNT^2+^·2Cl^–^ one of the best glucose binders reported to date.

**Scheme 2:**
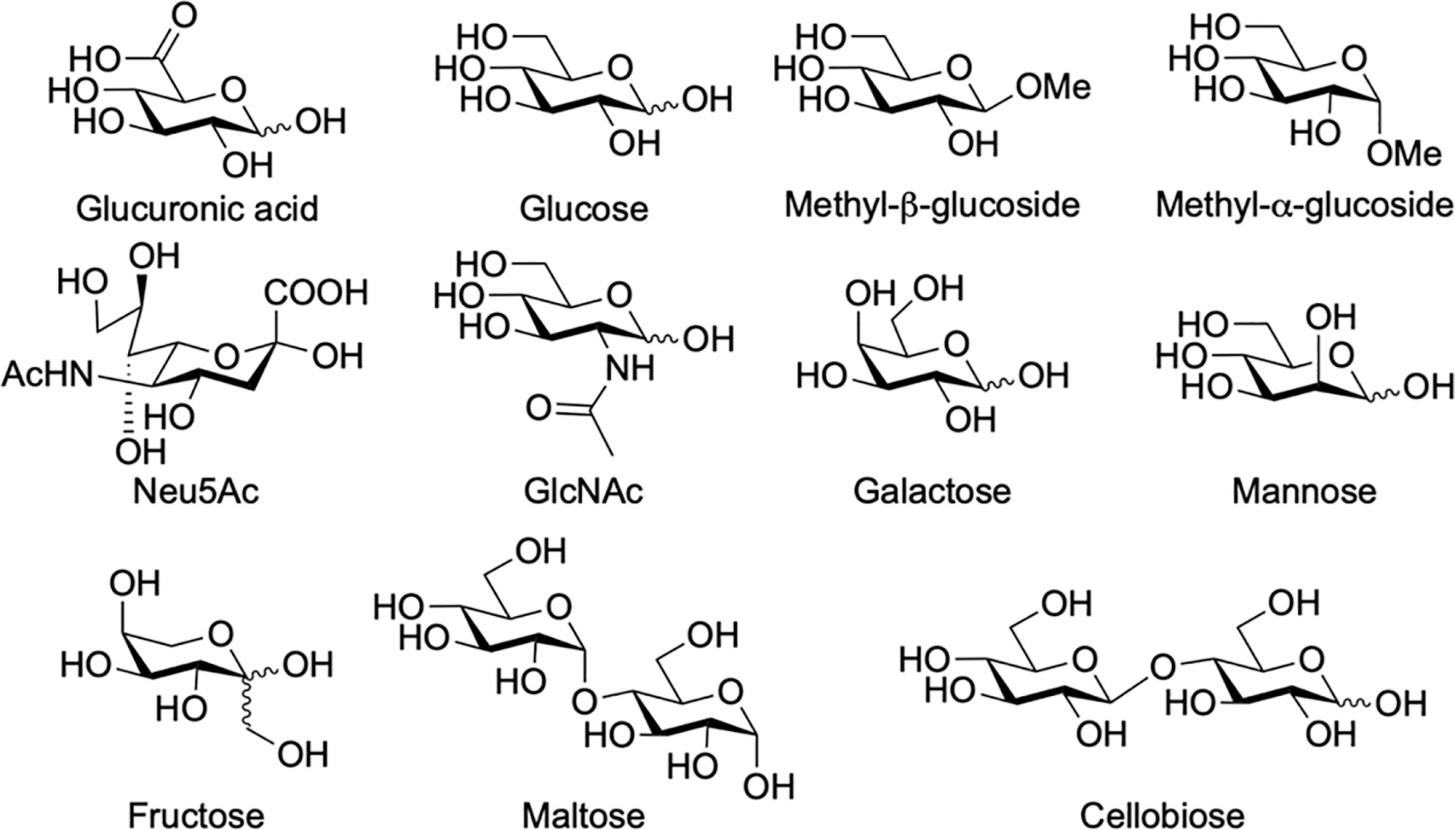
Structural Formulas of Carbohydrates Investigated

**Table 1:** Summary of Binding Constants between MPNT^2+^·2Cl^–^ and Carbohydrates Measured in H_2_O

		BPTL^2+^/*K*_a_ (M^–1^)		Δ*G*	Δ*H*	*TΔS*
Entry	Substrates^a^	NMR	ITC	(kcal/mol)	(kcal/mol)	(kcal/mol)
1	Glucuronic acid	>10^5^ ^b^	(1.0 ± 0.1) × 10^5^	–6.8	–7.1	–0.3
2	Neu5Ac	N.D.^c^	N.D.^e^	N.D.^e^	N.D.^e^	N.D.^e^
3	Glucose	1822 ± 162	1700 ± 83	–4.4	–4.8	–0.4
4	Methyl β-glucoside	769 ± 30	575 ± 31	–3.7	–3.1	0.6
5	Methyl α-glucoside	192 ± 5	252 ± 25	–3.2	–2.0	1.2
6	GlcNAc	174 ± 5	252 ± 33	–3.2	–0.8	2.4
7	Galactose	68 ± 1	52 ± 3	–2.3	–2.8	–0.5
8	Mannose	<10^d^	N.D.^e^	N.D.^e^	N.D.^e^	N.D.^e^
9	Fructose	<10^d^	N.D.^e^	N.D.^e^	N.D.^e^	N.D.^e^
10	Maltose	640 ± 90	745 ± 34	–3.9	–3.0	0.9
11	Cellobiose	713 ± 15	479 ± 45	–3.6	–2.8	0.8

a D-sugars were used throughout the study.

b Binding affinity is beyond the measurable range of NMR titration experiment.

c No change of chemical shift was observed.

d Binding affinities are beyond the lower limit of NMR titration experiment.

e Not measured on account of low affinity.

Notably, the binding of glucuronate is nearly 2 orders of magnitude stronger than that observed for glucose. Further thermodynamic analysis revealed that glucuronate binding incurs a similar entropy penalty to glucose but is accompanied by a markedly higher binding enthalpy. Given their structural similarity, both substrates likely adopt similar binding modes within the macrocycle. These findings indicate that the carboxylate group in glucuronate establishes a long-range electrostatic attraction with MPNT^2+^·2Cl^–^, contributing an additional ∼2.4 kcal/mol in binding energy and boosting its affinity into the 10^5^ M^–1^ range. In contrast, Neu5Ac, a larger anionic carbohydrate, exhibits no appreciable binding to MPNT^2+^·2Cl^–^. Its bulky size prevents^61^ encapsulation within the tetralactam macrocycle, thereby precluding the formation of hydrogen bonds and [C–H···π] interactions. This finding highlights that simple electrostatic attraction between the cationic macrocycle and an anionic substrate is insufficient to drive effective binding in water.

MPNT^2+^·2Cl^–^ exhibited good selectivity toward carbohydrates with an all-equatorial substitution pattern. Glucuronate, glucose, and methyl β-glucoside are representative examples of this configuration and displayed the highest binding affinities. Disaccharides containing a glucose unit, such as maltose and cellobiose, also showed good affinity for MPNT^2+^·2Cl^–^. In contrast, methyl α-glucoside exhibited reduced binding affinity compared to methyl β-glucoside, due to the α-linkage that alters the orientation of the C–H bond. GlcNAc, which replaces the C2–OH group of glucose with an −NHAc group, disrupted the hydrogen bonding pattern and led to a lower binding affinity of 252 M^–1^. Galactose, with an inverted C4–OH group, showed a markedly reduced affinity of 65 M^–1^. No binding was detected for mannose, which has an inverted C2–OH, or for fructose, which possesses an entirely different molecular framework. These findings highlight that carbohydrate binding is highly sensitive to molecular shape, size, and functional group distribution, all of which must complement the receptor’s binding pocket. Achieving such shape complementarity is essential for effectively binding hydrophilic substrates in water.

### Structure Elucidation by X-ray Crystallography and Computational Modeling

A single crystal of the glucuronate⊂MPNT^2+^ complex was obtained by slow evaporation of a solution containing MPNT^2+^·2Cl^–^ (0.45 mM) and glucuronic acid (3 mM) in water. X-ray analysis revealed a 1:1 binding stoichiometry, consistent with the results from our solution-phase studies. To further refine the structural details, the positions of hydrogen atoms were optimized using density functional theory (DFT) at the BLYP-gCP-D3BJ/SVP level, employing a water continuum model. The glucuronate substrate is positioned (Figure 3a and 3b) between the two naphthalene panels of the macrocycle, which are separated by 7.89 Å. All inward-facing N–H and polarized C–H groups engage in hydrogen bonding with the glucuronate, targeting its C3 and C4 hydroxyl groups, carboxylate moiety, and the pyranose oxygen. The [N–H···O] hydrogen bonds exhibit distances of 1.88–1.97 Å and angles of 169–174°, while the [C–H···O] interactions display distances of 2.01–2.20 Å and angles of 163–167°, indicative of a robust hydrogen-bonding network within the binding pocket.

**Figure 3:**
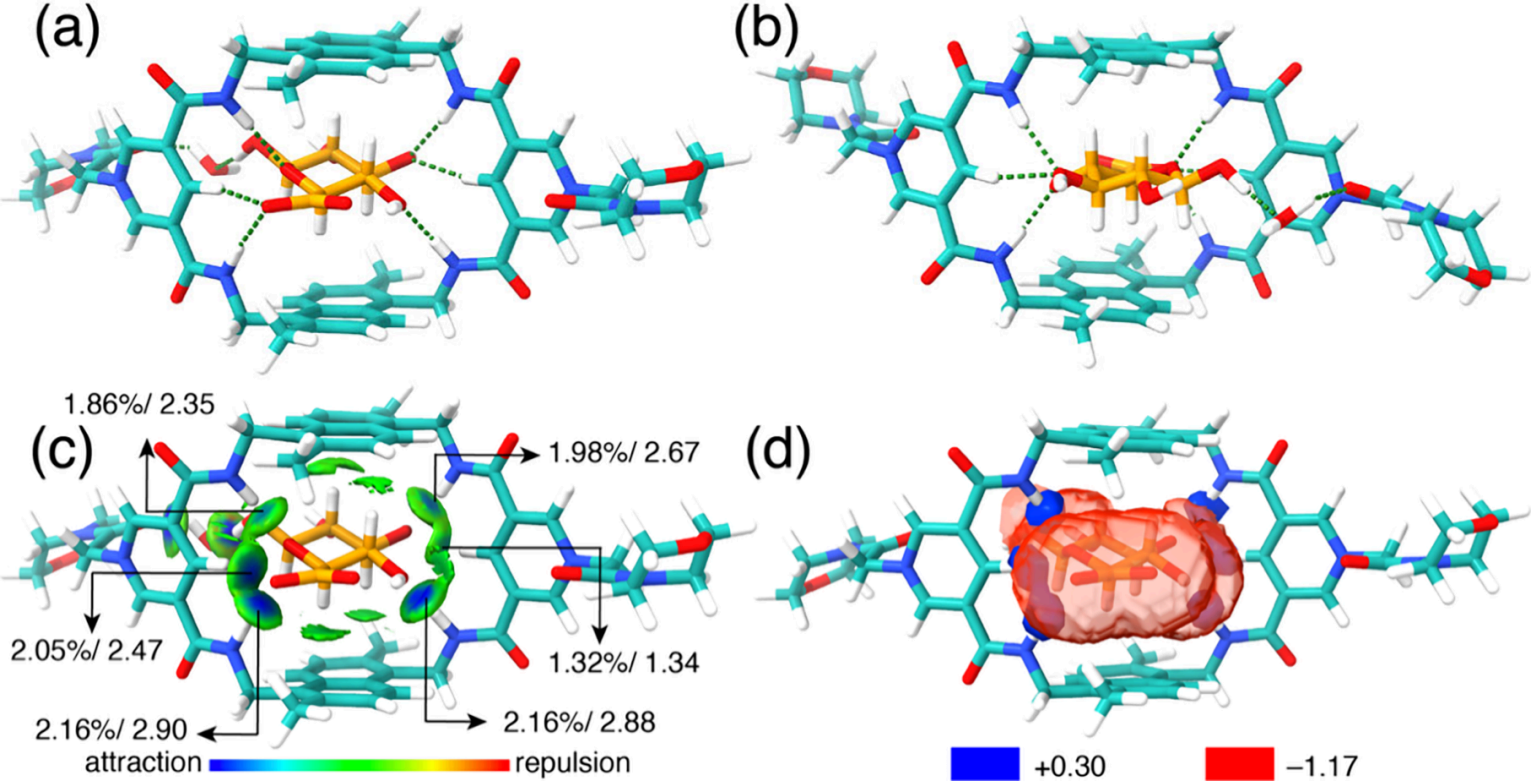
(a) Front and (b) rear view of X-ray single crystal structure (CCDC: 2451974) for MPNT^2+^ complexed with glucuronate. (c) IGMH analysis of X-ray single crystal structures of MPNT^2+^ complexed with glucuronate. Sign­(λ_2_)­ρ colored isosurfaces of δ*g* ^inter^ = 0.005 au. Isosurfaces are colored using a BGR scheme over the electron density range −0.05 < sign­(λ_2_)­ρ < 0.05 au The largest δ*G* ^pair^(%) terms derived from IGMH analysis are labeled before the slash. IBSIW values are labeled after the slash. (d) ESP isosurface of the macrocycles and glucuronate in the crystal structure. The red ESP isosurfaces represent regions of negative electrostatic potential of −1.17, and the blue ESP isosurfaces represent regions of positive electrostatic potential of +0.30.

Notably, a water molecule was observed (Figure 3b) bridging the C1 hydroxyl group of the glucuronate and the carbonyl group adjacent to the pyridinium nitrogen, suggesting that functional groups located outside the binding cavity can still contribute to substrate recognition through solvent-mediated interactions. In addition, this carbonyl group projects above the pyridinium ring, effectively preventing naphthalene panel stacking and thereby eliminating macrocycle aggregation.

An Independent Gradient Model based on Hirshfeld partitioning (IGMH) analysis (Figure 3c) was employed^62–65^ to visualize and quantify the weak interactions governing the glucuronate⊂MPNT^2+^ complex. This method utilizes reduced density gradient (RDG) isosurfaces to identify regions of noncovalent interactions, with interaction strength indicated by the sign­(λ_2_)­ρ color scale: blue denotes strong attractive forces such as hydrogen bonds, while green highlights weaker van der Waals interactions. In the solid-state structure, RDG isosurfaces corresponding to strong hydrogen bonds are observed in front of all inward-facing N–H and C–H bonds within the macrocyclic binding pocket. The relative strength of these interactions is quantitatively assessed using δ*G* ^pair^(%) values, shown before the slash in Figure 3c.

The analysis reveals that the carboxylate group of glucuronate engages in the strongest hydrogen bonds with both N–H and polarized C–H protons of MPNT^2+^. Remarkably, the [C–H···O] interaction with the carboxylate oxygen is stronger than several of the [N–H···O] hydrogen bonds formed with other oxygen atoms on the sugar. This finding underscores the critical role of the polarized C–H donors at the para position of the pyridinium ring in achieving the observed high binding affinity. It also rationalizes why pyridinium-bridged tetralactam macrocycles exhibit superior binding to sugars compared to analogous structures featuring isophthalamide spacers. In addition, the intrinsic bond strength index for weak interactions (IBSIW),^66,67^ calculated simultaneously from the IGMH analysis and presented after the slash in Figure 3c, correlates well with the δ*G* ^pair^(%) values, providing an independent validation of interaction strength.

Notably, the [C–H···π] interactions between the axial C–H protons of the sugar and the naphthalene panels were clearly visualized through the IGMH analysis. The C–H bonds at the C2 and C5 positions of the sugar were associated with larger interaction surfaces compared to those at the C3 and C4 positions, while no [C–H···π] interaction was detected for the C1 C–H bond. These qualitative insights help explain the observed sugar selectivity: axial-to-equatorial flips of C–H protons and hydroxyl groups at specific positions have differential impacts on binding affinity. For instance, only a modest decrease in affinity is observed when comparing methyl-β-glucoside to methyl-α-glucoside, as the C1 C–H bond does not contribute to [C–H···π] interactions, making its flip relatively inconsequential. In contrast, galactose exhibits higher affinity than mannose because mannose loses the critical [C–H···π] interaction at the C2 position, which is more influential for binding than the C4 position affected in galactose. Additionally, axial hydroxyl substitutions impose greater steric demands, causing these sugars to fit less optimally within the MPNT^2+^ binding pocket.

To illustrate the stereoelectronic complementarity between MPNT^2+^ and the glucuronate substrate, partial electrostatic potential (ESP) maps were generated^68,69^ (Figure 3d) using isovalues of +0.30 (blue) and −1.17 (red). The most electropositive regions of MPNT^2+^, depicted by blue isosurfaces, are localized around the inward-facing N–H and C–H groups. In contrast, the most electronegative regions of the glucuronate substrate, shown as red isosurfaces, are centered on the carboxylate group and hydroxyl oxygens. These positively charged regions of MPNT^2+^ extend into the anionic regions of the glucuronate, clearly visualizing the key zones of electrostatic attraction that drive binding within the complex.

### Chiroptical Sensing

To investigate the potential of MPNT^2+^·2Cl^–^ as a sensor for detecting changes in glucuronic acid concentrations in water, we examined its ability to generate^70^ binding-induced circular dichroism (CD) signals. MPNT^2+^·2Cl^–^ is intrinsically achiral and exhibits no CD signal, while glucuronic acid, although chiral, lacks a CD signal due to the absence of a chromophore. Upon complex formation, the highly directional hydrogen bonding between MPNT^2+^·2Cl^–^ and gluconate locks the macrocycle into a chiral conformation. This structural adaptation results in the emergence of an induced CD signal (Figure 4a). Titration of aqueous MPNT^2+^·2Cl^–^ solutions with glucuronic acid led (Figure 4b) to the gradual appearance of a distinct CD peak at 236 nm, corresponding to the absorption band of the pyridinium spacer. This observation indicates that effective chirality transfer is achieved through hydrogen bonding interactions. Notably, the high binding affinity between glucuronate and MPNT^2+^·2Cl^–^ enables sensitive monitoring of glucuronate at physiologically relevant concentrations from 0 to 300 μM in aqueous solution.^71^ These findings highlight the broader potential of synthetic receptors as versatile chiroptical sensors for tracking biologically important hydrophilic analytes in water, opening new avenues for diagnostic and analytical applications.

**Figure 4:**
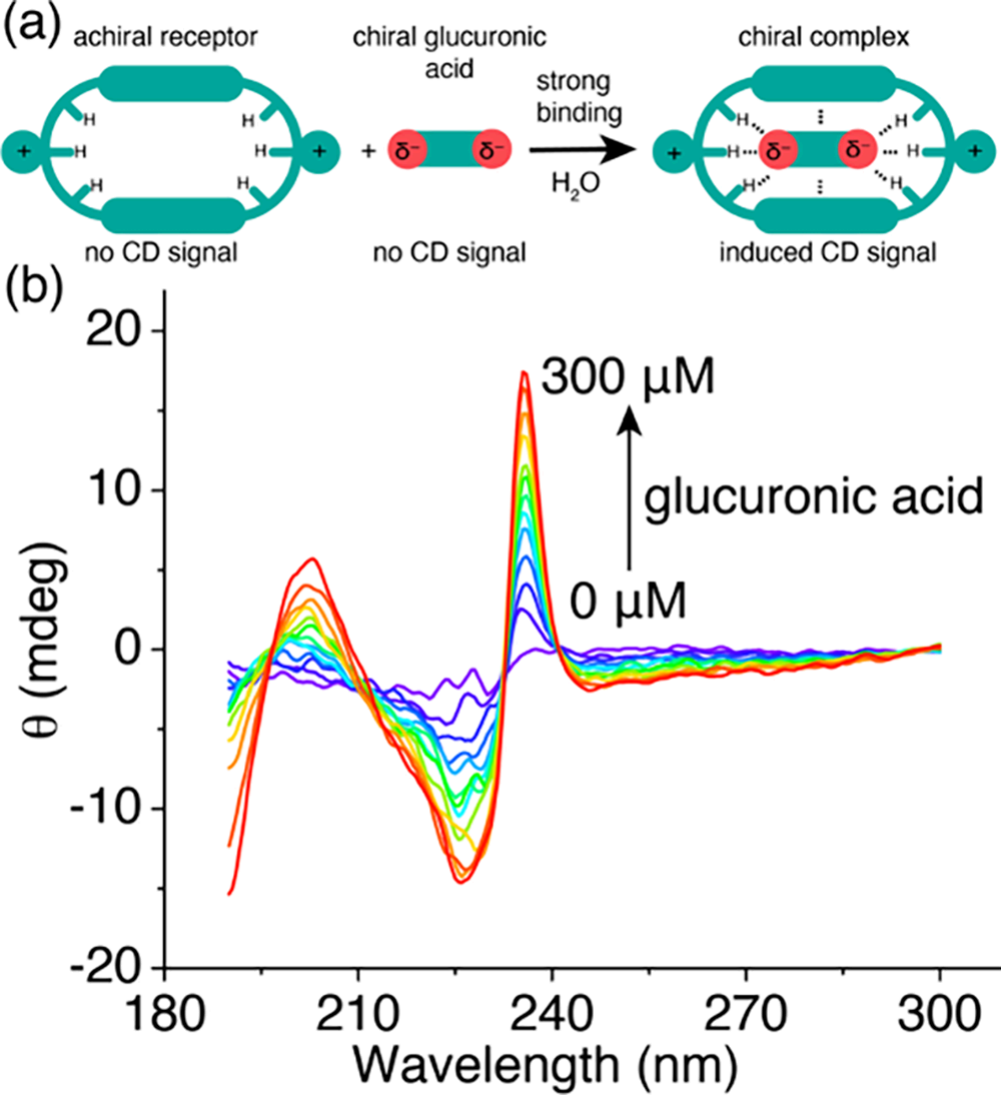
(a) Schematic illustration of binding-induced CD signal. (b) CD spectra of MPNT^2+^·2Cl^–^ (10 μM) with increasing glucuronic acid concentrations.

## Conclusion

In summary, we have developed a water-soluble tetralactam macrocycle, MPNT^2+^·2Cl^–^, that functions as a highly selective and potent synthetic lectin for glucuronate, achieving a binding affinity of 103,000 M^–1^—over 19-fold higher than the best previously reported receptors. By integrating pyridinium spacers, dimethylnaphthalene panels, and morpholine solubilizing groups, we successfully overcame longstanding challenges in aqueous carbohydrate recognition, including receptor aggregation, cavity collapse, and insufficient electrostatic complementarity. Structural analysis by X-ray crystallography, DFT optimization, and IGMH analysis revealed a dense network of hydrogen bonding, [C–H···π] interactions, and charge-assisted contacts that collectively drive high-affinity binding. Furthermore, we demonstrated that MPNT^2+^·2Cl^–^ can serve as a chiroptical sensor, producing induced circular dichroism signals that enable quantitative detection of glucuronic acid in water. This work provides one of the strongest and most selective synthetic receptors for anionic carbohydrates reported to date. Beyond its fundamental innovation in molecular design, this system offers a versatile platform for developing next-generation synthetic lectins, sensors, and diagnostic tools targeting biologically relevant hydrophilic substrates under physiological conditions. Such capabilities are of broad interest not only to supramolecular chemists but also to researchers in chemical biology, biomedical diagnostics, and environmental monitoring, where selective detection of polar biomolecules in aqueous media remains a critical challenge.

## Supplementary Material





## Data Availability

Accession Codes Deposition Numbers 2451974 contain the supplementary crystallographic data for this paper. These data can be obtained free of charge via the joint Cambridge Crystallographic Data Centre (CCDC) and Fachinformationszentrum Karlsruhe Access Structures service.

## Notes

Experimental methods, synthetic procedures, compound characterization, mass spectrometry, NMR spectroscopy, ITC, UV/vis absorption and fluorescence emission spectroscopy, CD spectroscopy, X-ray crystallography, and computational analysis details (PDF)
(CIF) X-ray single crystal structure (CCDC: 2451974) for MPNT2+ complexed with glucuronate.
