# Supplementary material for: A Synthetic Lectin for Glucuronate
Source: ACS Cent Sci. 2025 Aug 1;11(9):1753–61. doi: 10.1021/acscentsci.5c00951 (PMC12464755; doi:10.1021/acscentsci.5c00951)
Supplement: Supplementary file 1 [file oc5c00951_si_001.pdf]

Supporting information for

A Synthetic Lectin for Glucuronate

Canjia Zhai, Chengkai Xu, Yunpeng Cui, Lukasz Wojtas, Jianfeng Cai, Wenqi Liu*.

Department of Chemistry, University of South Florida, Tampa, FL, 33620, USA

Corresponding Author Email: wenqi@usf.edu

Table of contents

1. General Methods. S2

2. Synthesis and Compound Characterization S3

3. Mass Spectrometry S4

4. NMR Spectroscopy S7

5. Isothermal Titration Calorimetry S24

6. UV/Vis Absorption and Fluorescence Spectroscopy S35

7. Circular Dichroism Spectroscopy S36

8. X-Ray Crystallography Data and Analysis S37

9. Computational Analysis S39

10. References S42

1. General Methods.

All commercially available solvents and chemicals were purchased from Sigma-Aldrich, Fisher Scientific, and Ambeed and used without further purification unless otherwise stated. Starting materials **1** (2,6-dimethylnaphthalene-1,5-diyl)dimethanamine[S1] and compound **2** pyridine-3,5-dicarbaldehyde[S2] were prepared by the reported literature. Water was deionized and micro-filtered through a Milli-Q water filtration system. Reactions were monitored by analytical thin-layer chromatography (TLC) on silica gel 60-F_{254} plates, visualized by ultraviolet (254 nm) light. Nuclear magnetic resonance (NMR) spectra were recorded on a Varian Unity Inova 600 MHz spectrometer or a Varian Unity Inova 400 MHz system. Chemical shift was presented in ppm and referenced by residual non-deuterated solvent peaks ($CDCl_3$: δ = 7.26 ppm, D_2O: δ = 4.79 ppm, DMSO-d_6: δ = 2.50 ppm). High-resolution mass spectrometry (HRMS) was obtained on an Agilent LC-MS QTOF 6540 using an ESI source. High-resolution mass spectrometry (HRMS) for the receptor complex with chloride and glucose was performed by a Waters Synapt G2 mass spectrometer using an ESI source. Matrix-assisted laser desorption/ionization-time of flight (MALDI-TOF) mass spectrometry was performed by a Bruker UltraFlextreme spectrometer using trans-2-[3-(4-tert-Butylphenyl)-2-methyl-2-propenylidene]malononitrile as a matrix substance. Isothermal titration was performed on the MicroCal iTC$_{200}$ system. ITC Data were analyzed on MicroCal iTC$_{200}$ software. UV-Vis absorption spectra were collected by a Thermo Scientific Evolution 201 UV/Vis Spectrometer. Fluorescence spectra were collected on a Horiba Scientific Fluoromax-4 Spectrofluorometer. Circular dichroism spectra were recorded on the JASCO J-1500 Circular Dichroism Spectrophotometer. Detailed experimental procedures are provided below in the appropriate sections of this supplementary information.

2. Synthesis and Compound Characterization

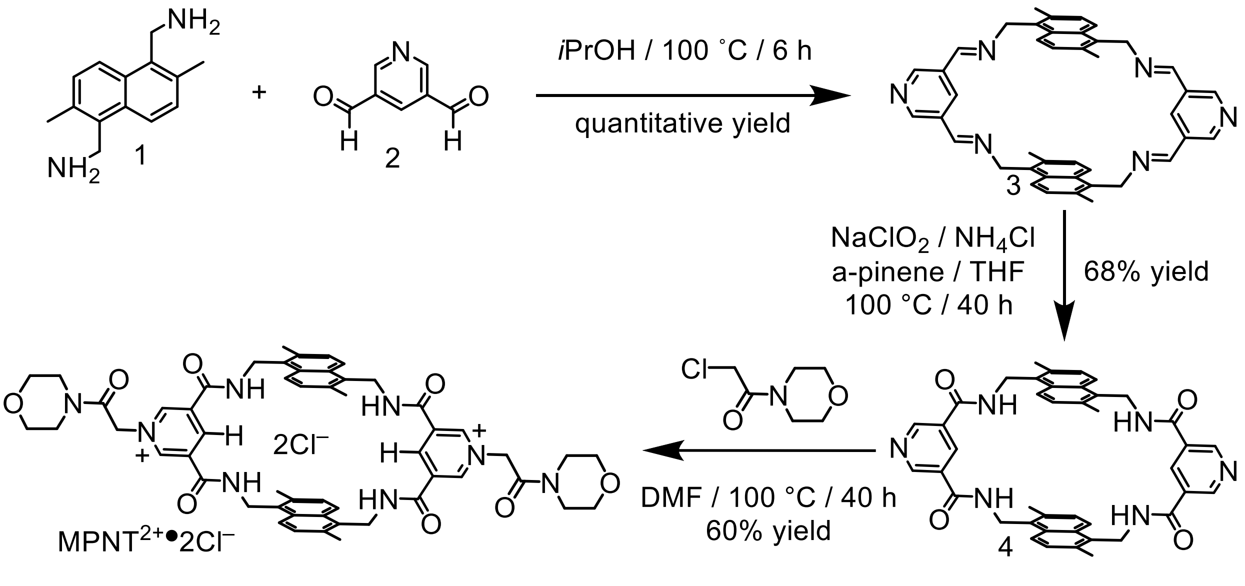

Scheme S1. Synthesis of $MPNT^{2+}$•$2Cl^{-}$.

Imine macrocycle 3: A solution of compound 1 (295 mg, 1.38 mmol, one equiv) and compound 2 (186 mg, 1.38 mmol, 1 equiv) in isopropanol (3 mL) was heated at 100 °C for 6 hours. After the reaction mixture had cooled to room temperature, the solvent was removed under reduced pressure using a rotary evaporator. The resulting yellow solid, corresponding to imine macrocycle 3, was obtained quantitatively and used in subsequent experiments without further purification. ^{1}H NMR (600 MHz, $CDCl_3$) δ 9.15 (s, 2H), 9.07 (s, 2H), 8.04 (s, 2H), 7.90 (s, 2H), 7.85 (d, J = 8.7 Hz, 2H), 7.78 (s, 1H), 7.69 (d, J = 8.7 Hz, 2H), 7.56 (s, 1H), 7.29 (d, J = 9.3 Hz, 4H), 5.55 (d, J = 16.8 Hz, 2H), 5.47 (d, J = 17.5 Hz, 2H), 5.23 (d, J = 17.4 Hz, 2H), 5.11 (d, J = 16.8 Hz, 2H), 2.40 (s, 12H). ^{13}C NMR (151 MHz, $CDCl_3$) δ 157.9, 150.5, 149.9, 141.6, 136.5, 135.4, 134.7, 134.6, 131.7, 130.8, 129.9, 124.1, 123.9, 56.6, 55.9, 19.9, 19.8. MS(MALDI-TOF) *m/z* cald for $C_{42}H_{39}N_6^{+}$: 627.32 $[M\text{+H}]^{+}$; found 627.31. Note: A pair of conformers was observed attributable to the rotation of the naphthalene panels.

Tetralactam macrocycle 4: A solution of imine macrocycle 3 (0.68 mmol, 1 equiv), $NaClO_2$ (1.47 g, 16 mmol, 24 equiv), NH_4Cl (290 mg, 5.4 mmol, 8 equiv), and α-pinene (4.3 mL, 27 mmol, 40 equiv) in anhydrous THF (20 mL) was stirred at 100 °C for 48 hours. After completion, the solvent was removed under reduced pressure using a rotary evaporator. The resulting residue was washed with water (12 mL × 3) to obtain the crude product, followed by sequential washing with DMF (3 mL × 2) and acetone (10 mL × 3) to afford tetralactam macrocycle 4 as a pale yellow solid (319

mg, 68% yield). ^{1}H NMR (400 MHz, DMSO-d_6) δ 9.11 (s, 4H), 8.80 (s, 4H), 8.14 (s, 2H), 7.89 (d, J = 5.0 Hz, 4H), 7.24 (d, J = 8.8 Hz, 5H), 5.04 (s, 3H), 4.64 (s, 3H). ^{13}C NMR (151 MHz, $CDCl_3$) δ 162.4, 162.2, 162.0, 161.9, 161.6, 145.0, 142.2, 135.7, 133.0, 131.3, 130.4, 127.4, 123.6, 117.3, 115.4, 113.5, 111.6, 39.8, 19.3. HRMS(ESI) *m/z* cald for $C_{42}H_{38}N_6NaO_4^+$: 713.2847 [*M*+Na]$^+$; found 713.2825.

MPNT^{2+}•2Cl$^-$: A solution of amide macrocycle 4 (50 mg, 0.0724 mmol, 1 equiv) and 4-(chloroacetyl)morpholine (474 mg, 2.9 mmol, 40 equiv) in DMF (3 mL) was stirred at 100 °C for two days. After cooling to room temperature, the reaction mixture was suspended in EtOAc (20 mL) and centrifuged. The resulting precipitate was washed sequentially with EtOAc (35 mL × 2) and acetonitrile (20 mL × 2) to afford the product MPNT^{2+}·2Cl$^-$ as a yellow solid (45 mg, 60% yield). ^{1}H NMR (400 MHz, D_2O) δ 9.47 (s, 4H), 9.20 (s, 2H), 7.89 (d, J = 8.7 Hz, 4H), 7.40 (d, J = 8.7 Hz, 4H), 6.02 (s, 4H), 5.02 (s, 8H), 3.91 (s, 4H), 3.82 (s, 5H), 3.70 (s, 8H), 2.48 (s, 14H). ^{13}C NMR (151 MHz, D_2O/CD_3CN (5/1)) δ 162.6, 161.2, 147.8, 139.9, 134.7, 132.2, 130.3, 129.0, 127.7, 122.9, 118.3, 65.2, 65.0, 61.5, 44.2, 42.0, 37.3, 18.0. HRMS(ESI) *m/z* cald for $C_{54}H_{58}N_8O_8^{2+}$: 473.2184 [*M*-2Cl]$^{2+}$; found 473.2195.

3. Mass Spectrometry

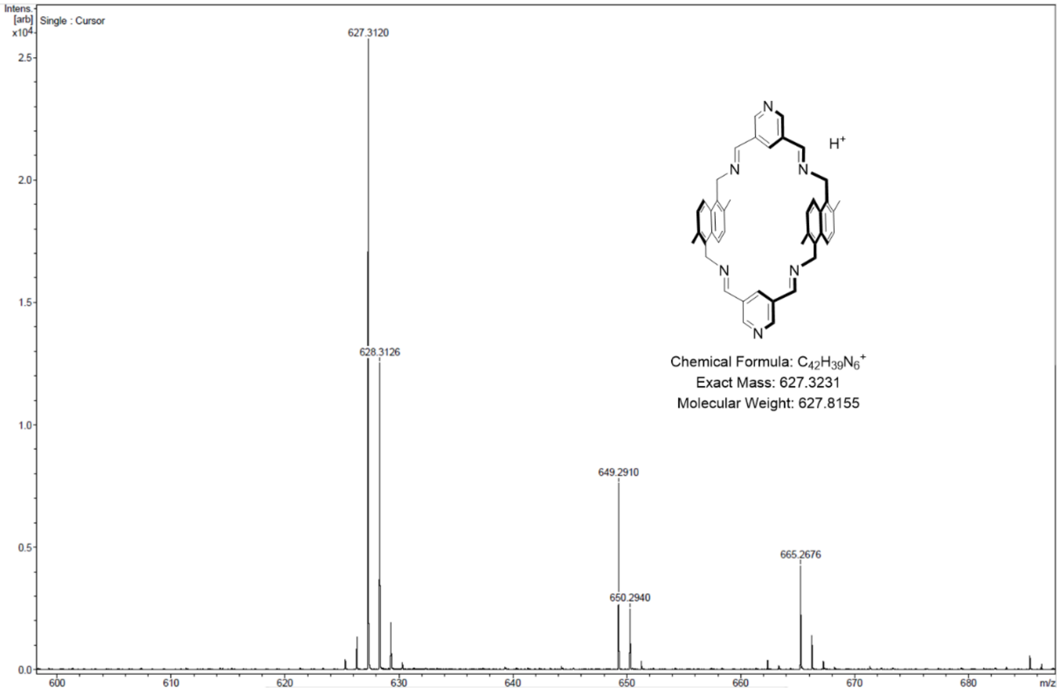

Figure S1. MALDI-TOF spectrum of imine macrocycle 3.

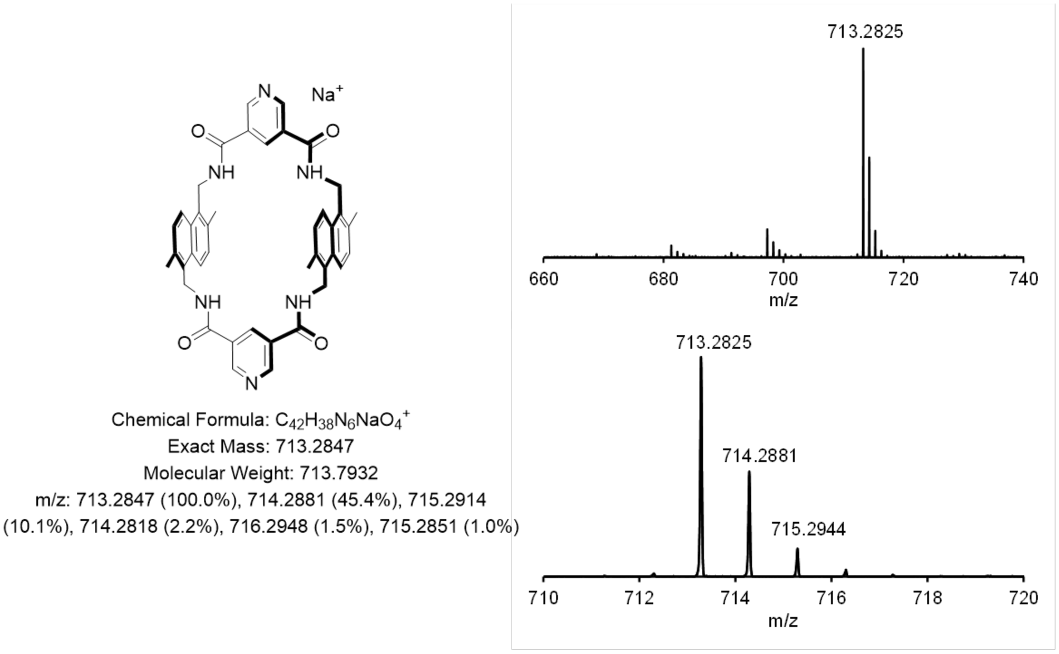

Figure S2. HRMS(ESI) spectrum of tetralactam macrocycle 4.

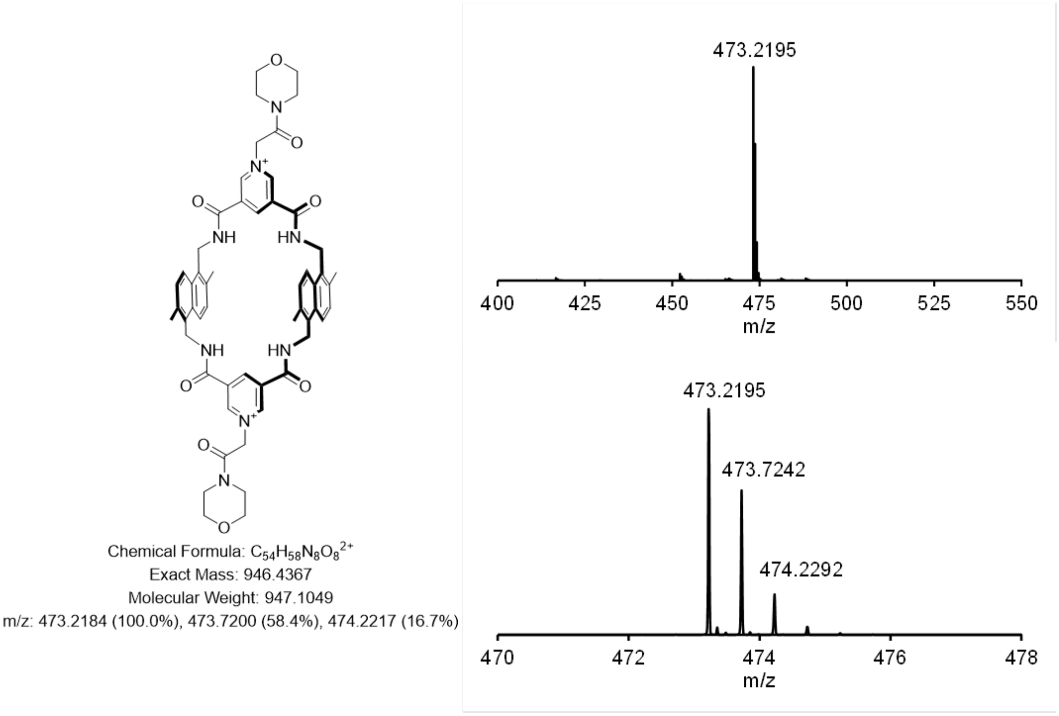

Figure S3. HRMS(ESI) spectrum of $MPNT^{2+}$•$2Cl^{-}$.

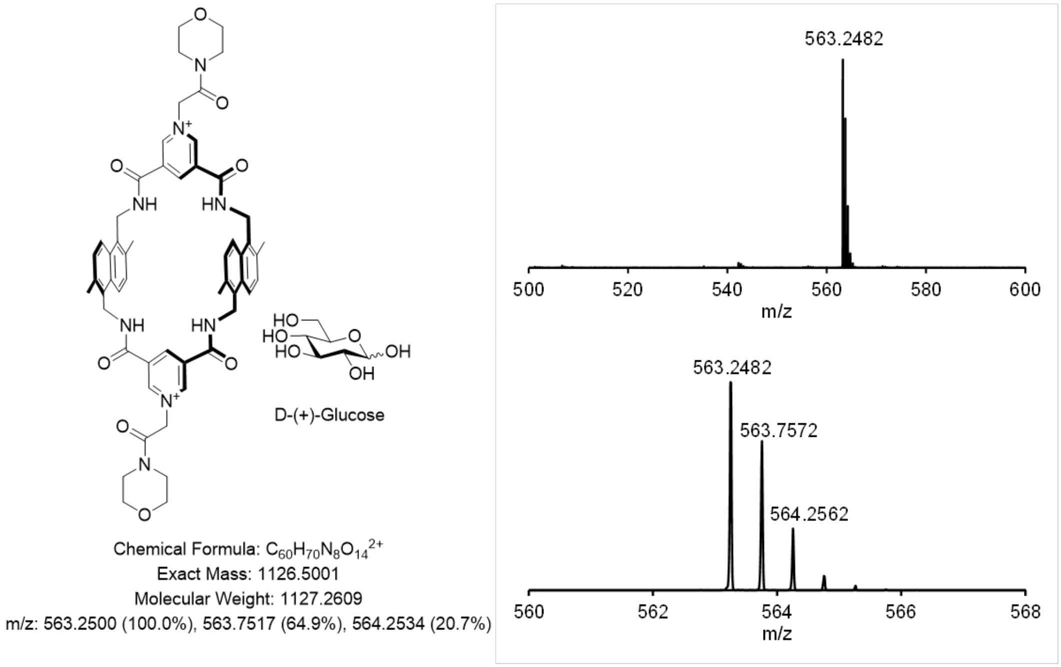

Figure S4. HRMS(ESI) spectrum of complex glucose⊂ MPNT^{2+}.

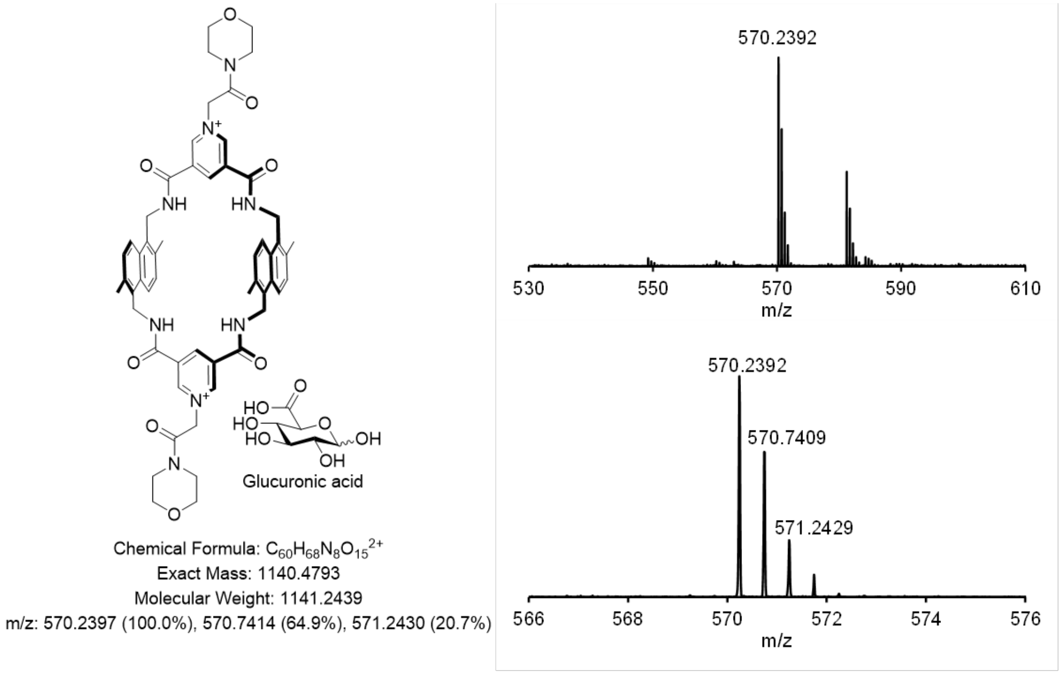

Figure S5. HRMS(ESI) spectrum of complex glucuronic acid⊂MPNT^{2+}.

4. NMR Spectroscopy

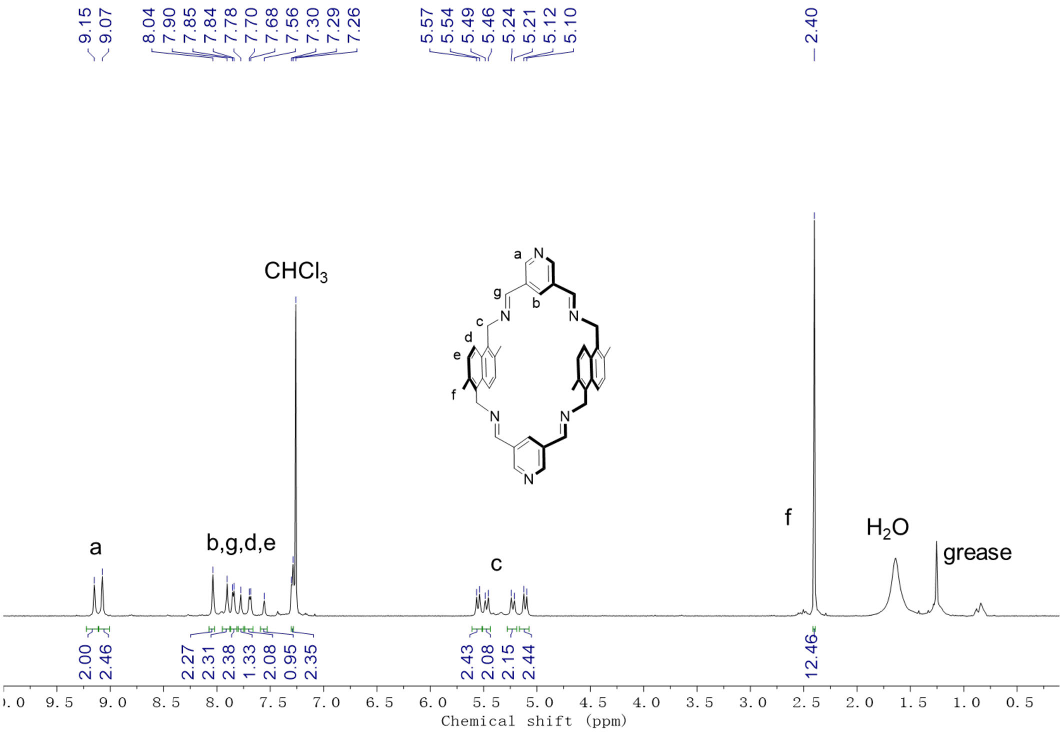

Figure S6. ^{1}H NMR spectrum (400 MHz, $CDCl_3$, 298 K) of imine macrocycle **3**.

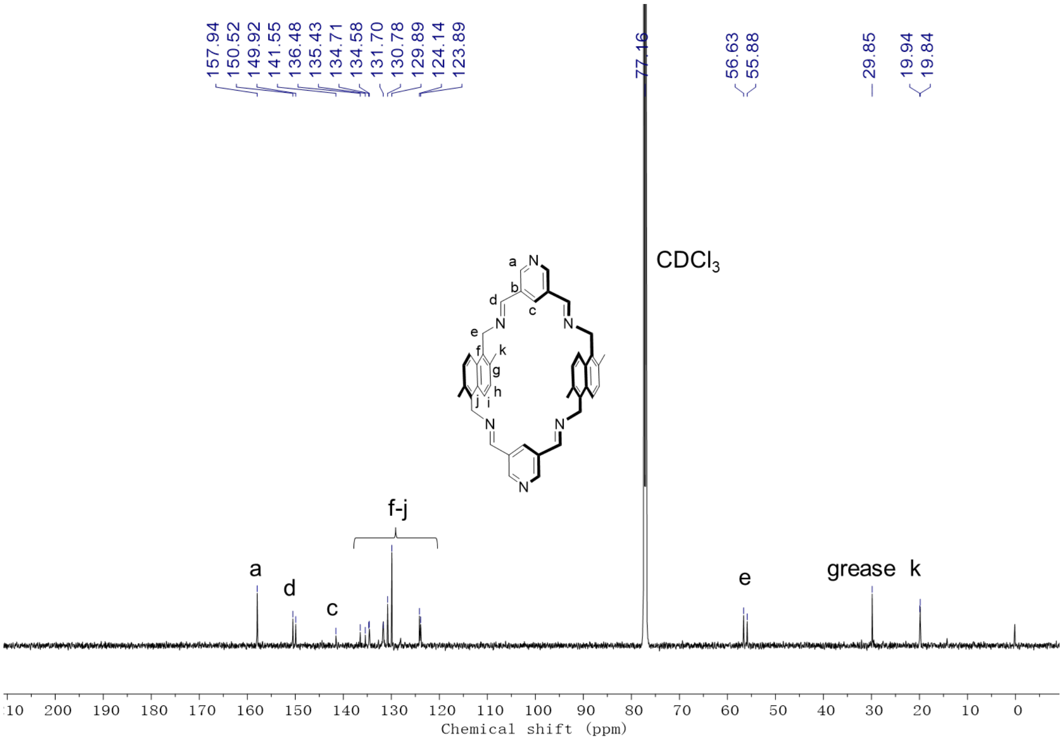

Figure S7. ^{13}C NMR spectrum (151 MHz, $CDCl_3$, 298 K) of imine macrocycle **3**.

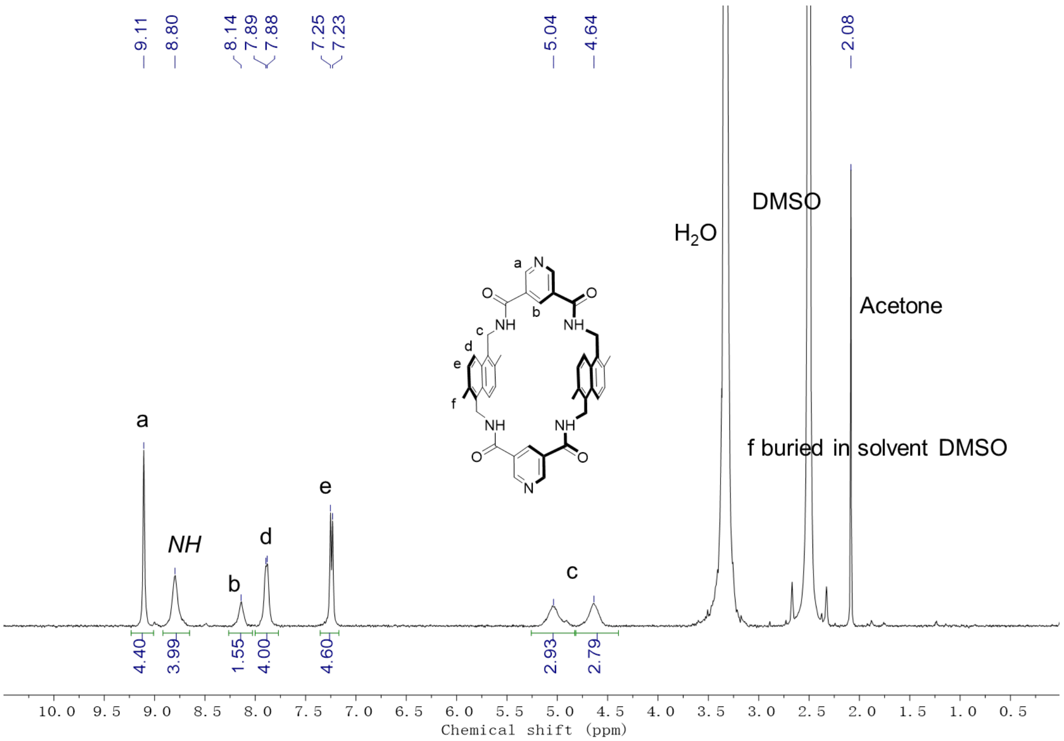

Figure S8. ^{1}H NMR spectrum (400 MHz, DMSO-d_6, 298 K) of tetralactam macrocycle 4.

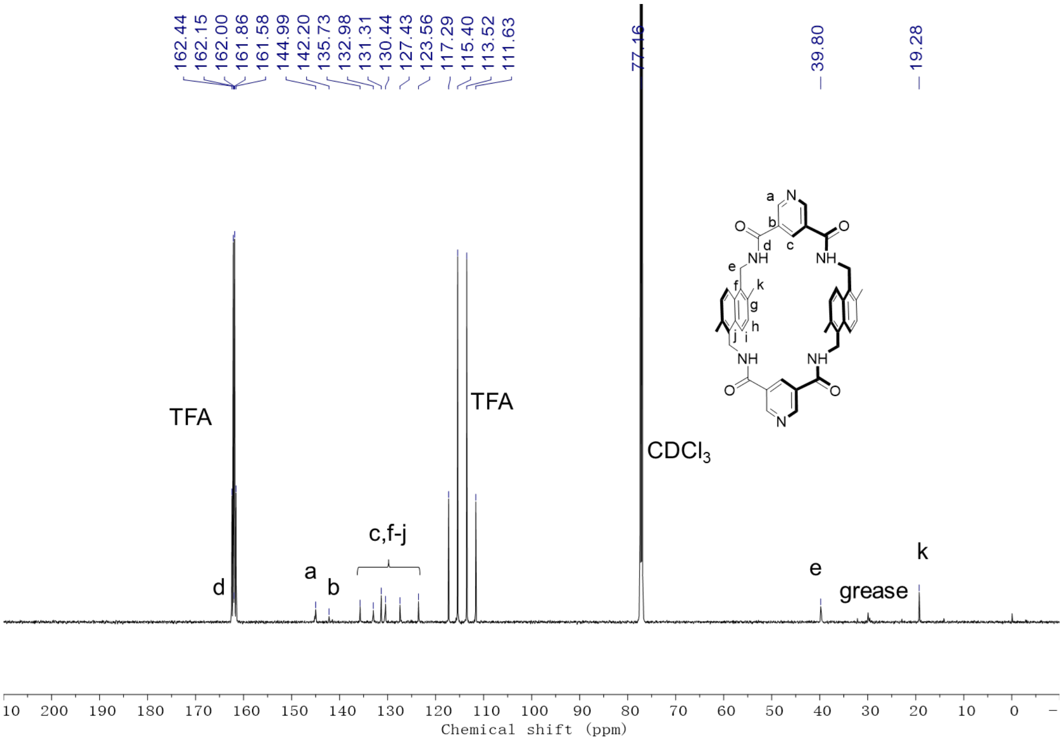

Figure S9. ^{13}C NMR spectrum (151 MHz, $CDCl_3$, 298 K) of tetralactam macrocycle 4 with TFA as additive to increase solubility.

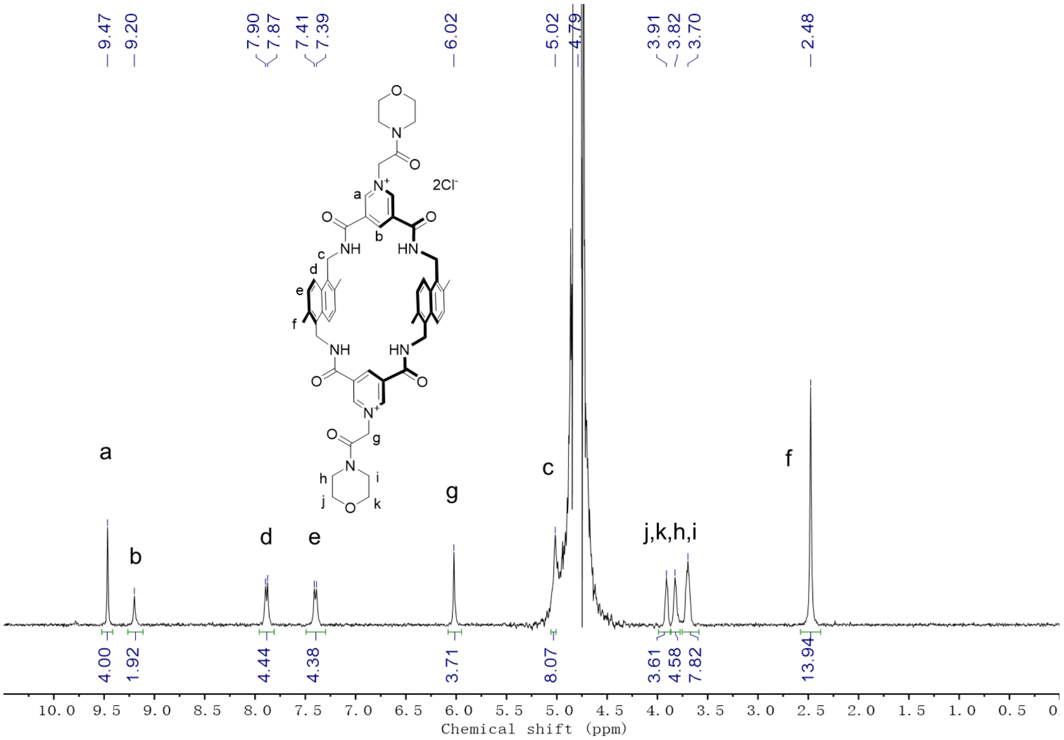

Figure S10. ^{1}H NMR spectrum (400 MHz, D_2O, 298 K) of compound $MPNT^{2+}$•$2Cl^{-}$.

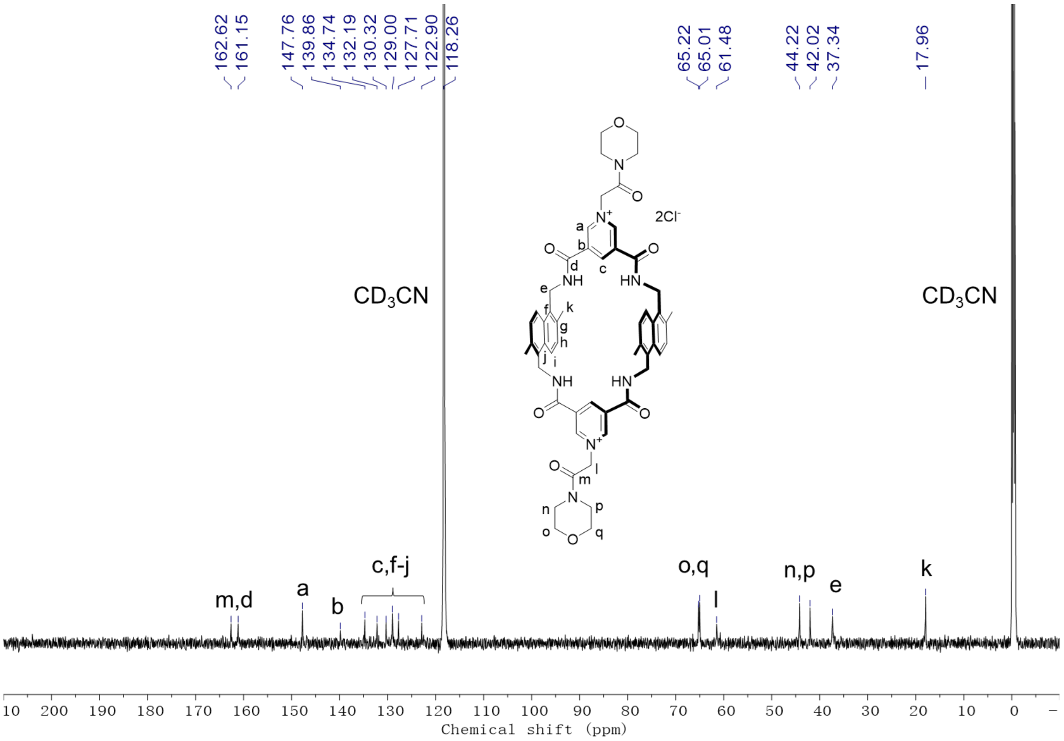

Figure S11. ^{13}C NMR spectrum (151 MHz, D_2O/CD_3CN (5/1), 298 K) of compound $MPNT^{2+}$•$2Cl^{-}$.

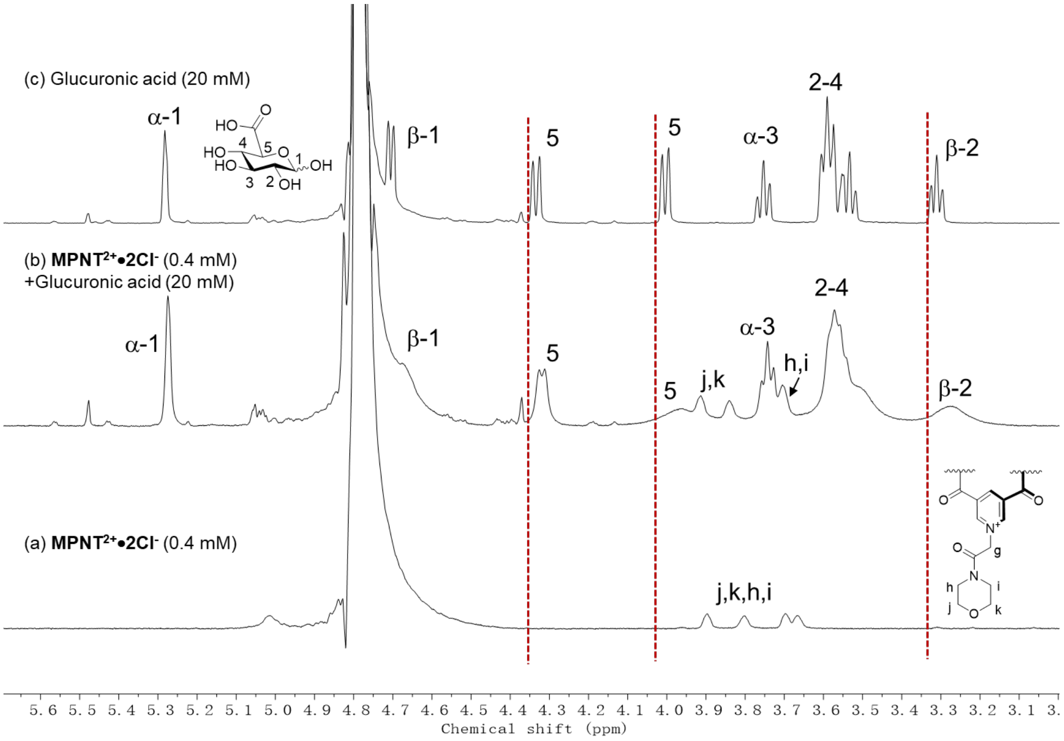

Figure S12. Partial ^{1}H NMR spectra (600 MHz, D_2O, 298 K) of (a) $MPNT^{2+}$•$2Cl^-$. (0.4 mM), (b) a mixture of $MPNT^{2+}$•$2Cl^-$. (0.4 mM) and glucuronic acid (20 mM), and (c) glucuronic acid (20 mM).

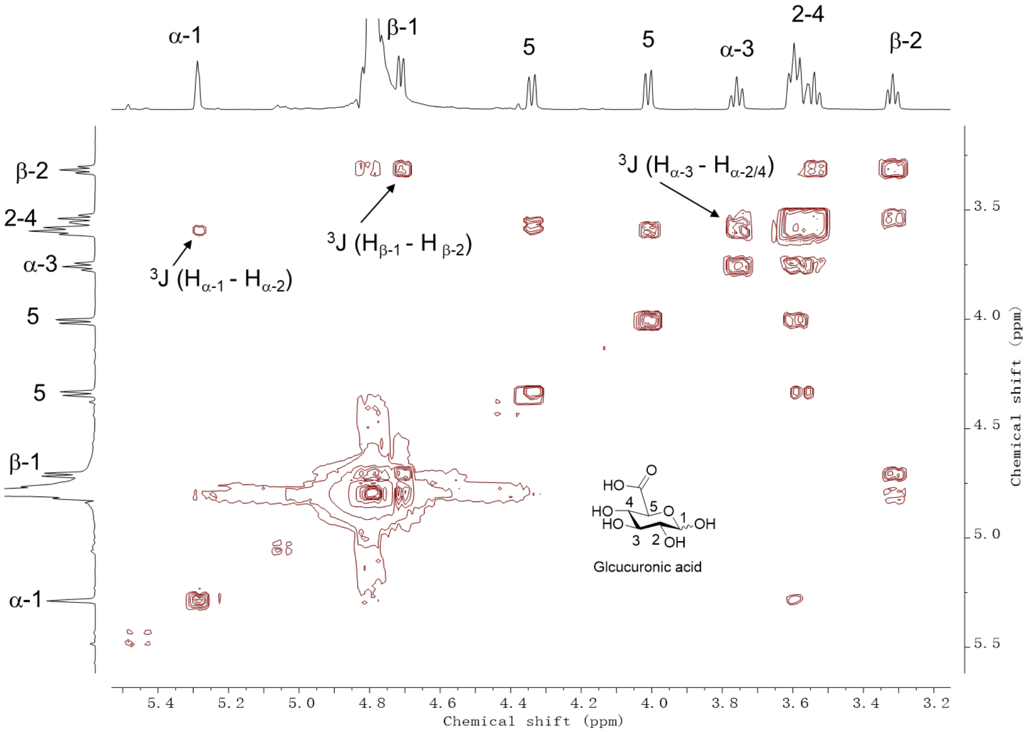

Figure S13. ^{1}H-^{1}H gCOSY NMR spectrum (600 MHz, D_2O, 298 K) of Glucuronic acid (20 mM).

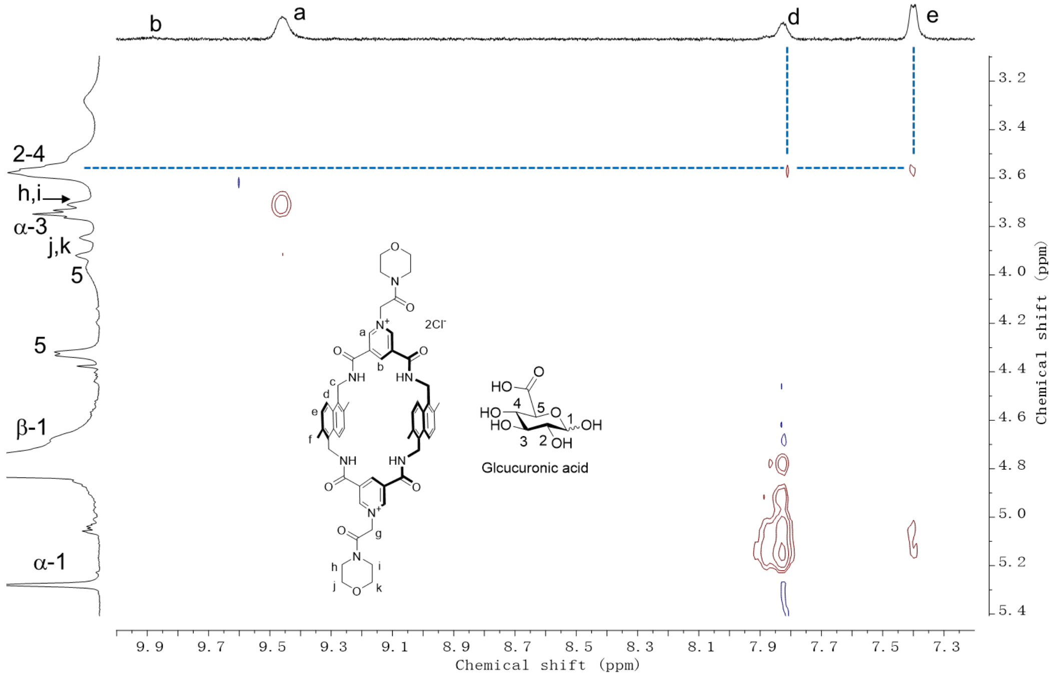

Figure S14. Partial ^{1}H-^{1}H NOESY NMR spectrum (600 MHz, D_2O, 298 K) of a mixture of glucuronic acid (20 mM) and **MPNT^{2+}** (0.4 mM). The mixing time for data acquisition is set at 0.2 seconds.

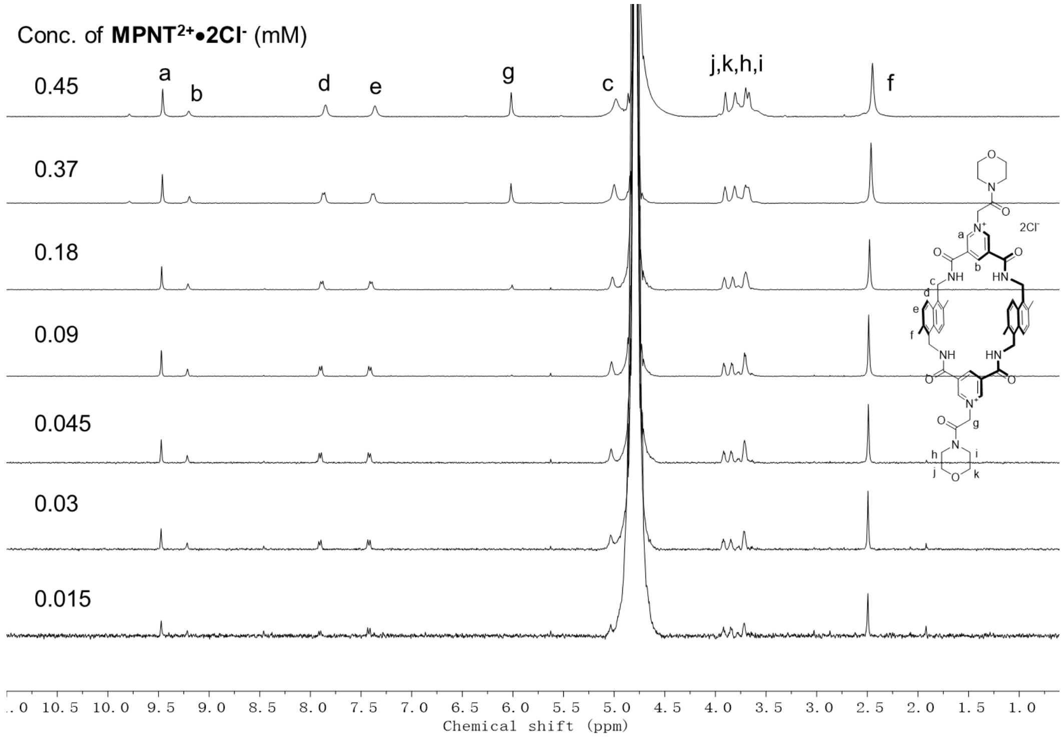

Figure S15. ^{1}H NMR spectrum (400 MHz, D_2O, 298 K) of compound MPNT^{2+}•2Cl^{-} at different concentrations.

Binding Analysis Using ^{1}H NMR Titration

^{1}H NMR titrations in D_2O were conducted at 298 K on a Varian Unity Inova 400 MHz system with a cryoprobe or a Varian Unity Inova 600 MHz spectrometer. Aliquots from a stock solution containing the corresponding carbohydrate were added sequentially to an NMR tube containing the solution of **MPNT^{2+}•2Cl$^-$** (600 μL). The ^{1}H NMR spectrum was acquired after each addition. The ^{1}H NMR titration spectra were analyzed using MestReNova software. The NMR titration isotherms were fitted[S3,S4] to a 1:1 host-guest binding model using Thordarson's equations at http://app.supramolecular.org/bindfit/. The data were then plotted using OriginLab software. The binding constants K_a were presented with standard deviations from the fitting outcomes.

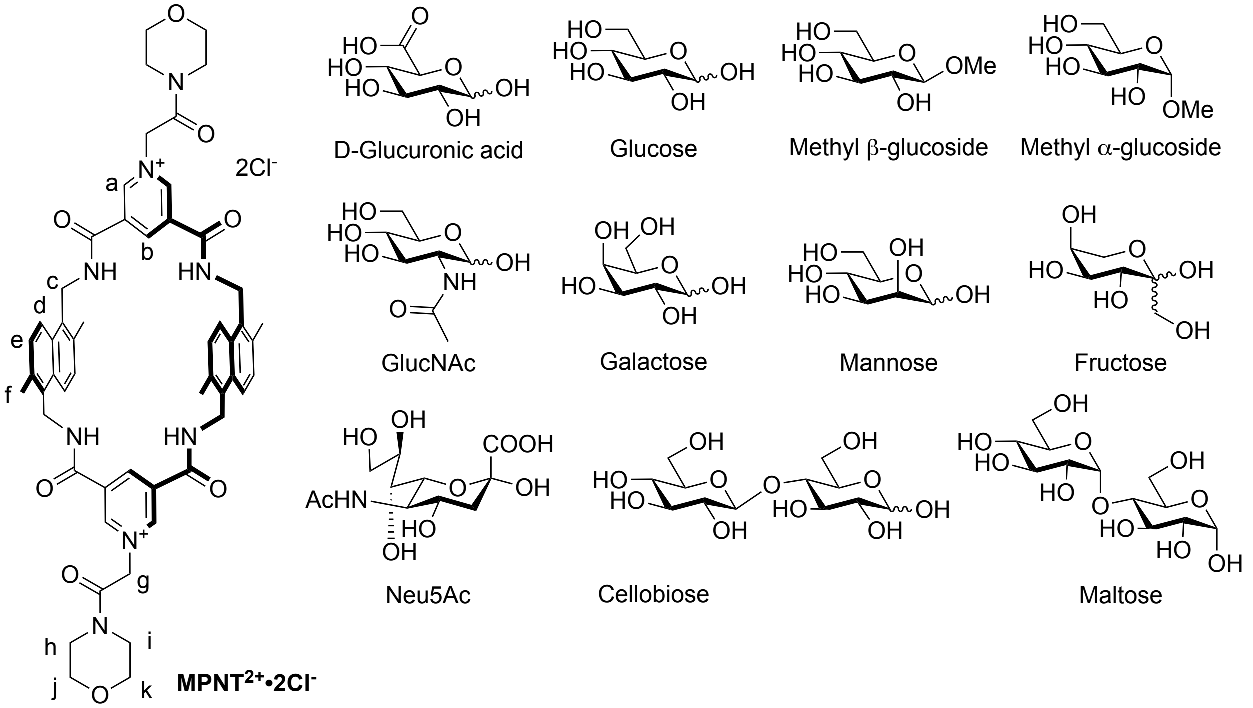

Figure S16. The structural formula of MPNT^{2+}•2Cl$^-$ with label and carbohydrate substrates, was investigated in this work.

^{1}H NMR Titration of $MPNT^{2+}$•$2Cl^-$ with carbohydrates

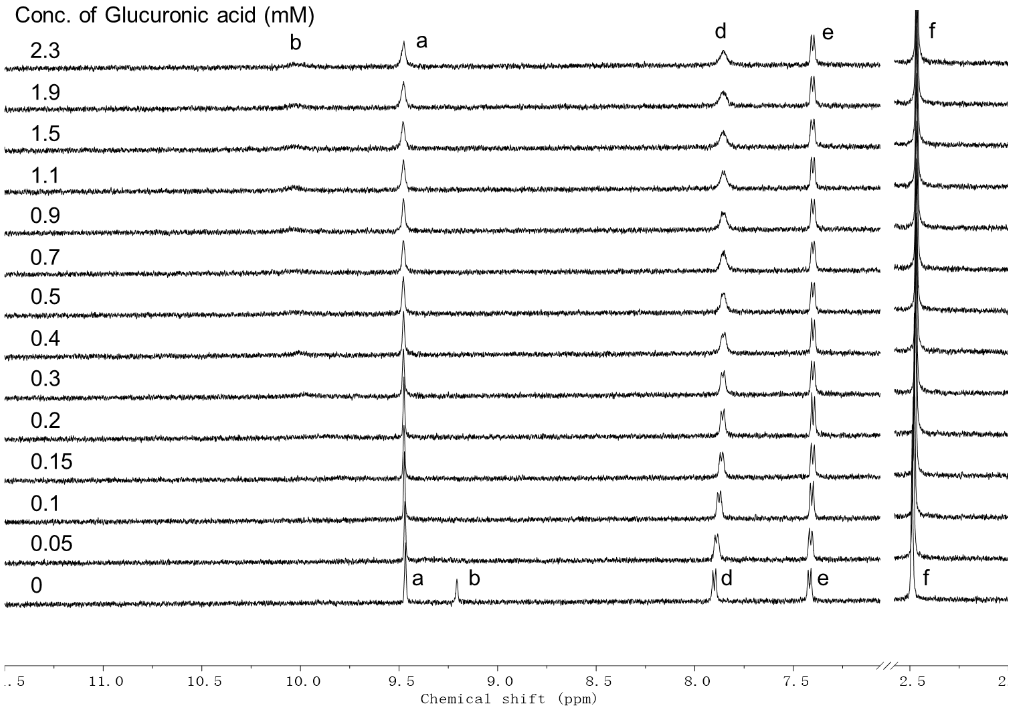

Figure S17. ^{1}H NMR spectra (600 MHz, D_2O, 298 K) of **$MPNT^{2+}$•$2Cl^-$** (0.15 mM) titrated with glucuronic acid.

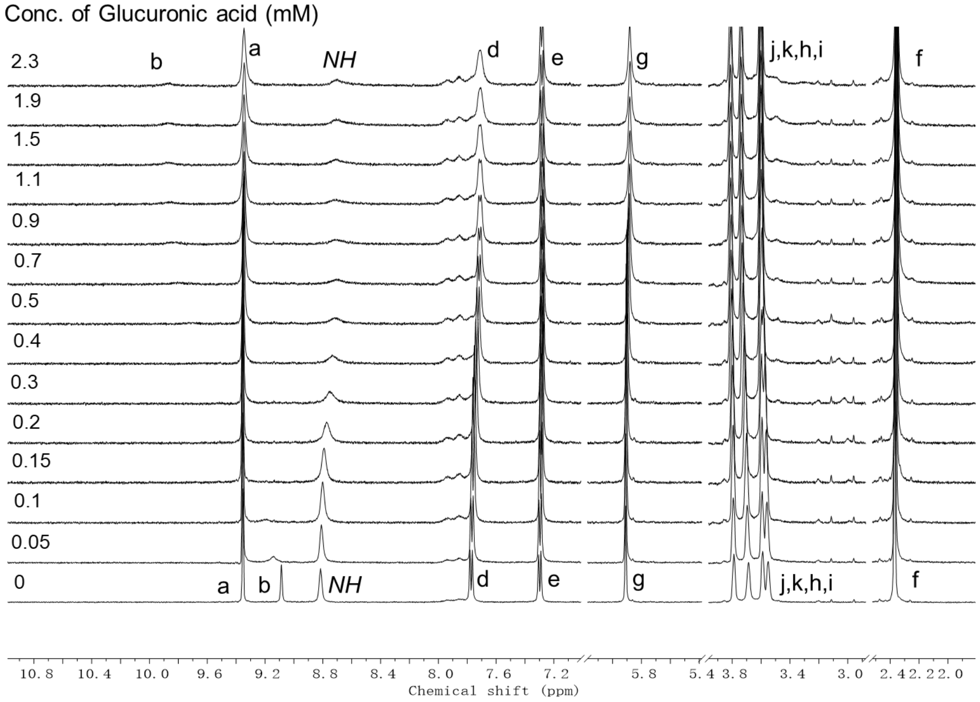

Figure S18. ^{1}H NMR spectra (600 MHz, 98% H_2O+ 2% D_2O, 298 K) of $MPNT^{2+}$•$2Cl^-$ (0.45 mM) titrated with glucuronic acid.

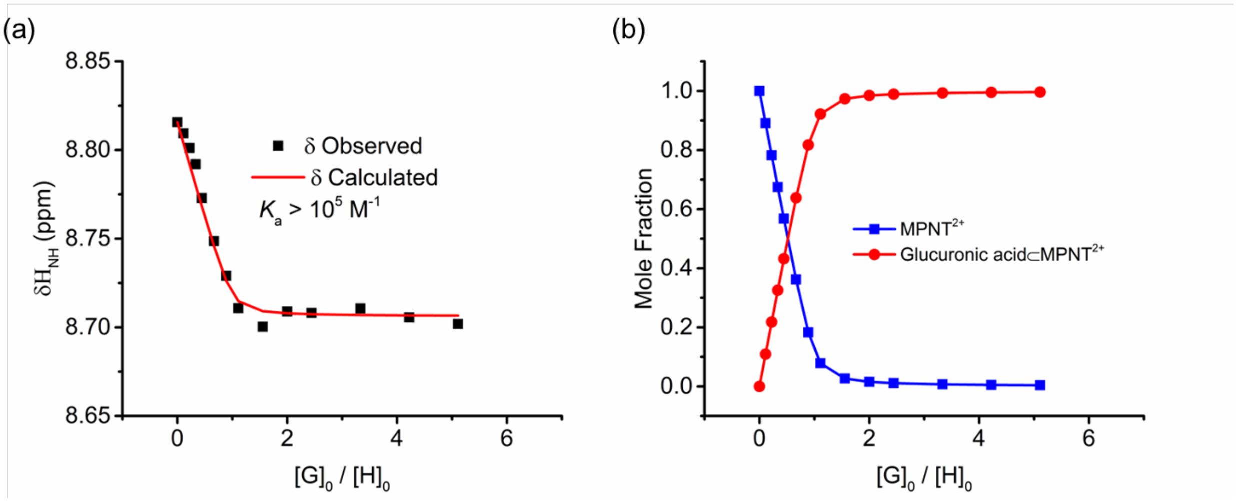

Figure S19. (a) Titration isotherm obtained by monitoring the chemical shift changes of amide NH of $MPNT^{2+}•2Cl^{-}$ (0.45 mM) upon the incremental addition of glucuronic acid in 98% H_2O+ 2% D_2O at 298 K. Red curves represent the best fit using a 1:1 host–guest binding model. (b) Calculated changes of mole fractions for $MPNT^{2+}•2Cl^{-}$ (blue trace) and glucuronic acid⊂$MPNT^{2+}$ (red trace) over the host-guest mole ratio.

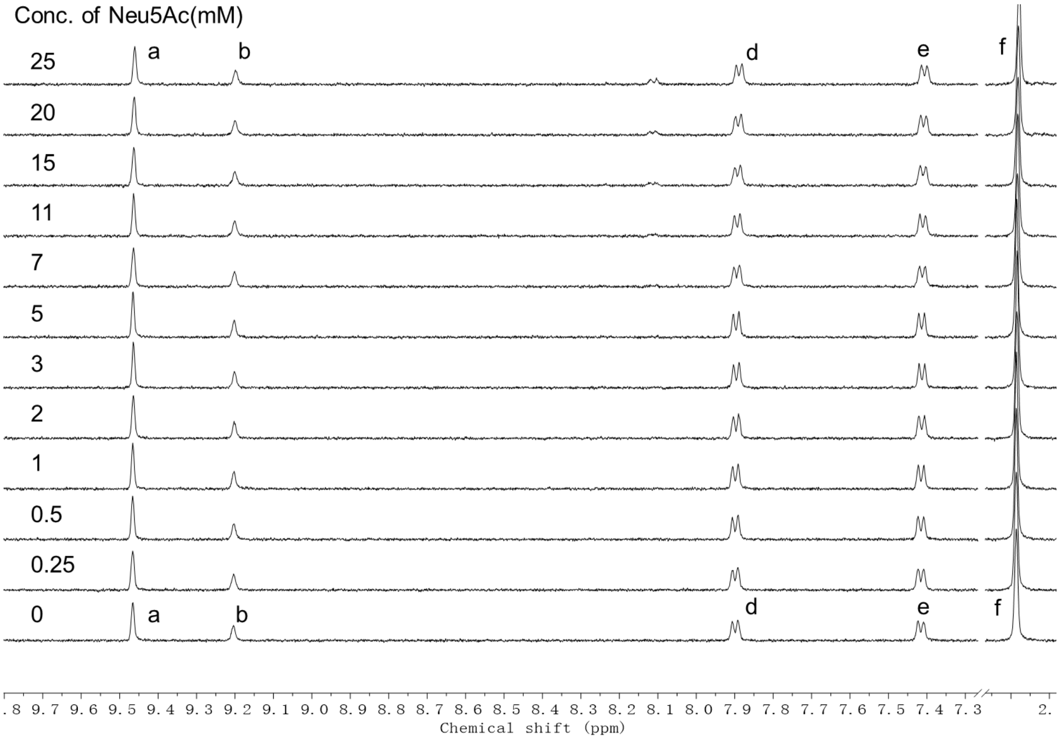

Figure S20. ^{1}H NMR spectra (600 MHz, D_2O, 298 K) of $MPNT^{2+}•2Cl^{-}$ (0.15 mM) titrated with Neu5Ac.

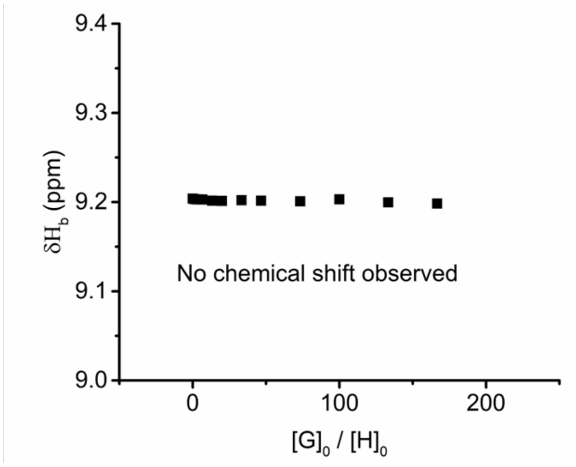

Figure S21. Titration isotherm obtained by monitoring the chemical shift changes of proton b of $MPNT^{2+}$•$2Cl^{-}$ (0.15 mM) upon the incremental addition of Neu5Ac in D_2O at 298 K.

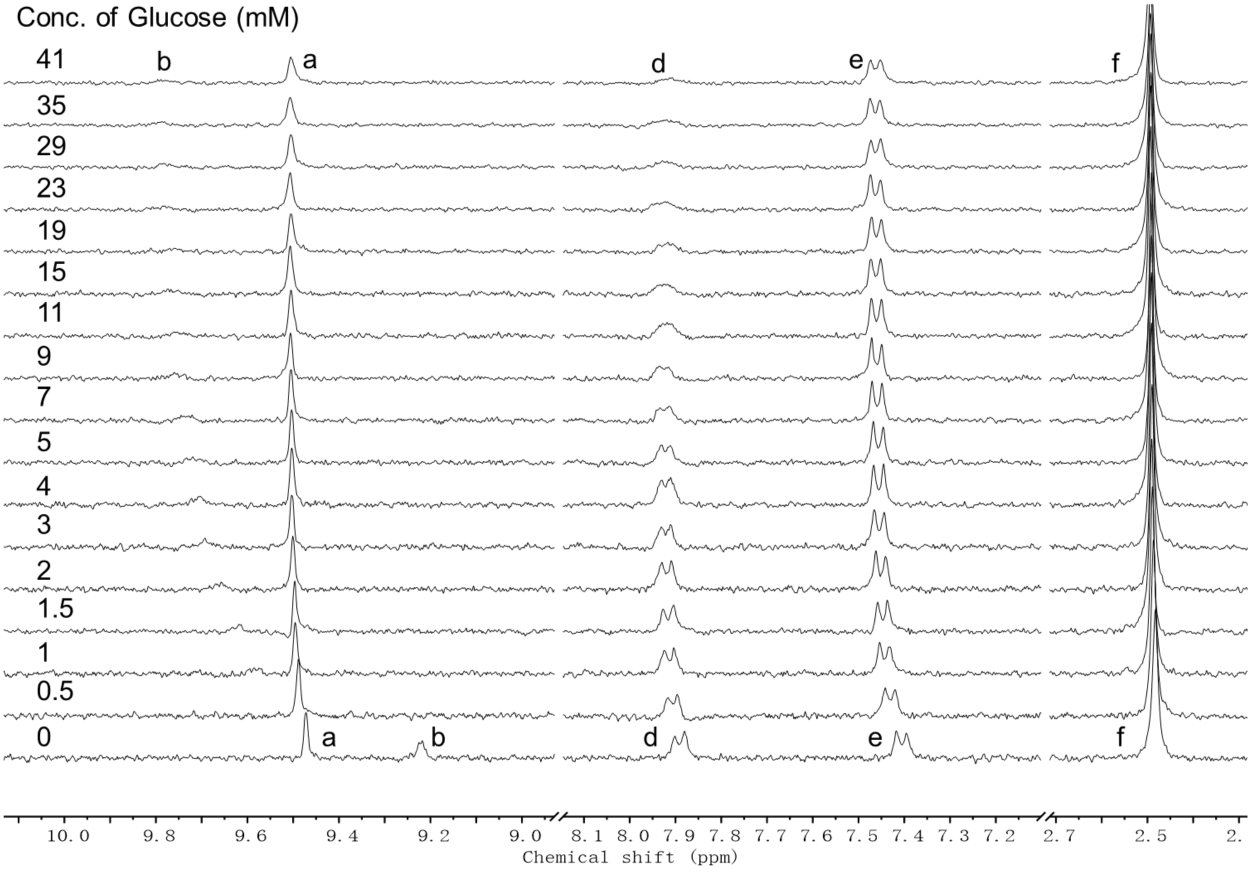

Figure S22. ^{1}H NMR spectra (400 MHz, D_2O, 298 K) of $MPNT^{2+}$•$2Cl^{-}$ (0.15 mM) titrated with glucose.

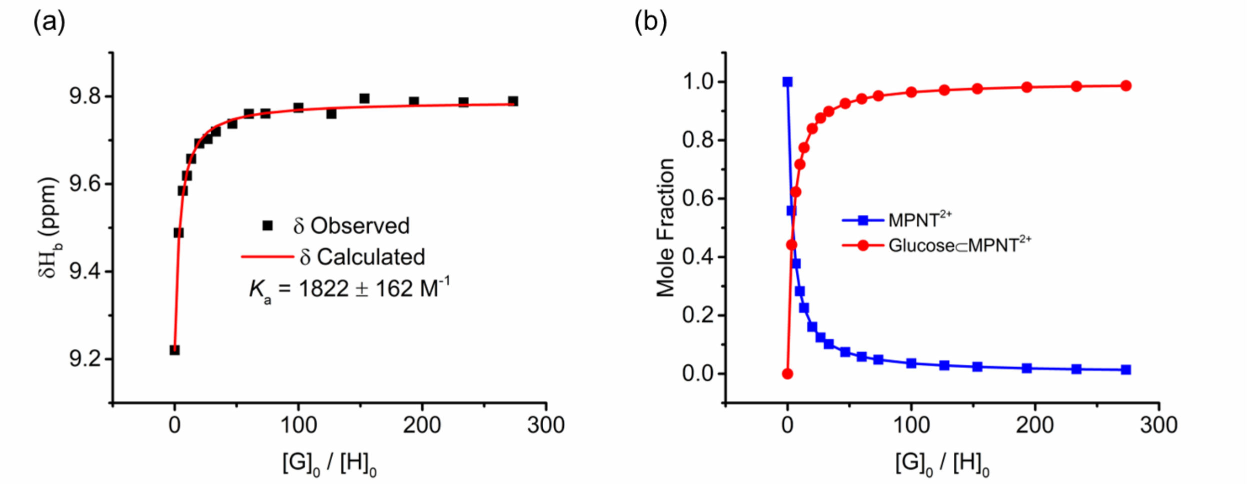

Figure S23. (a) Titration isotherm obtained by monitoring the chemical shift changes of proton b of $MPNT^{2+}$•$2Cl^-$ (0.15 mM) upon the incremental addition of glucose in D_2O at 298 K. Red curves represent the best fit using a 1:1 host–guest binding model. (b) Calculated changes of mole fractions for $MPNT^{2+}$•$2Cl^-$ (blue trace) and glucose⊂$MPNT^{2+}$ (red trace) over the host-guest mole ratio.

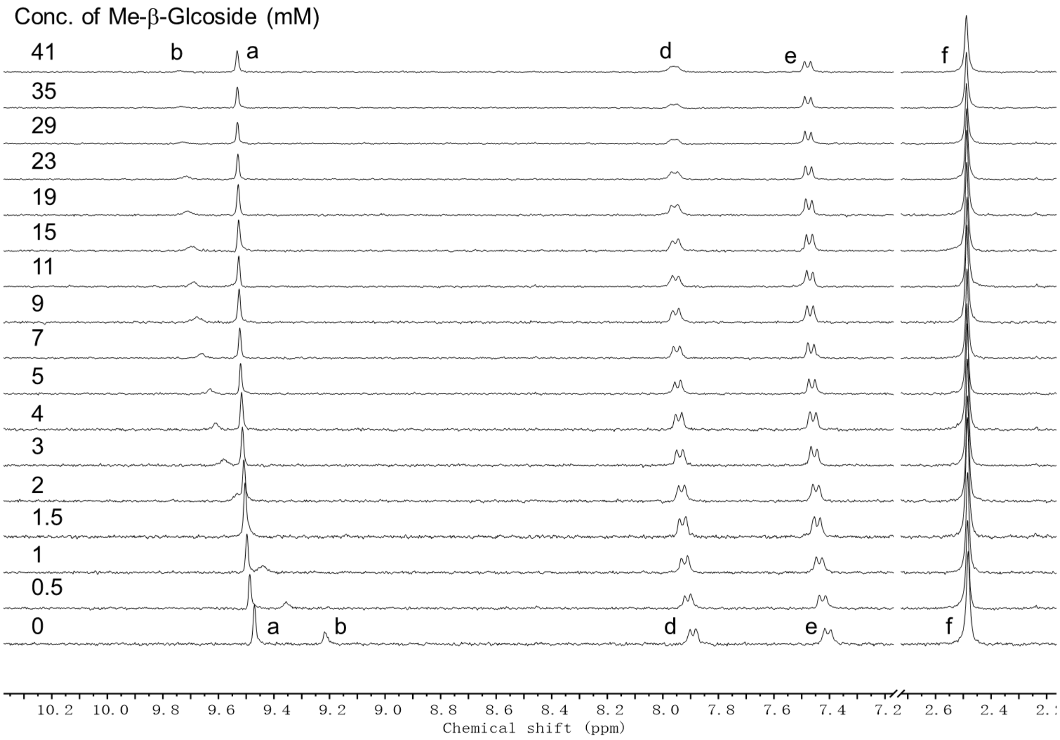

Figure S24. ^{1}H NMR spectra (400 MHz, D_2O, 298 K) of **$MPNT^{2+}$•$2Cl^-$** (0.15 mM) titrated with Methyl β-glucoside.

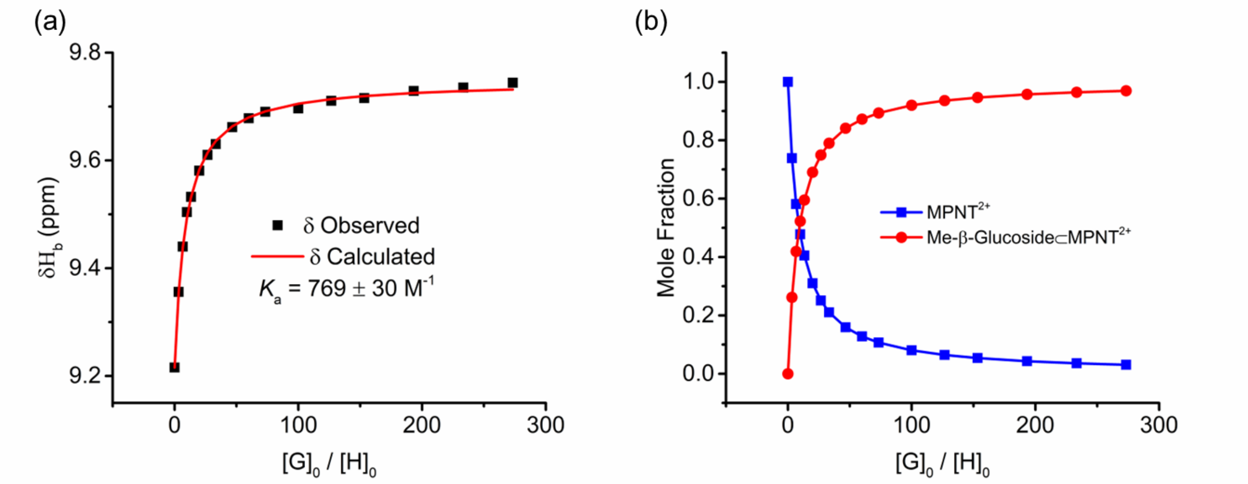

Figure S25. (a) Titration isotherm obtained by monitoring the chemical shift changes of proton b of $MPNT^{2+}$•$2Cl^-$ (0.15 mM) upon the incremental addition of Methyl β-glucoside in D_2O at 298 K. Red curves represent the best fit using a 1:1 host–guest binding model. (b) Calculated changes of mole fractions for $MPNT^{2+}$•$2Cl^-$ (blue trace) and Methyl β-glucoside⊂$MPNT^{2+}$ (red trace) over the host-guest mole ratio.

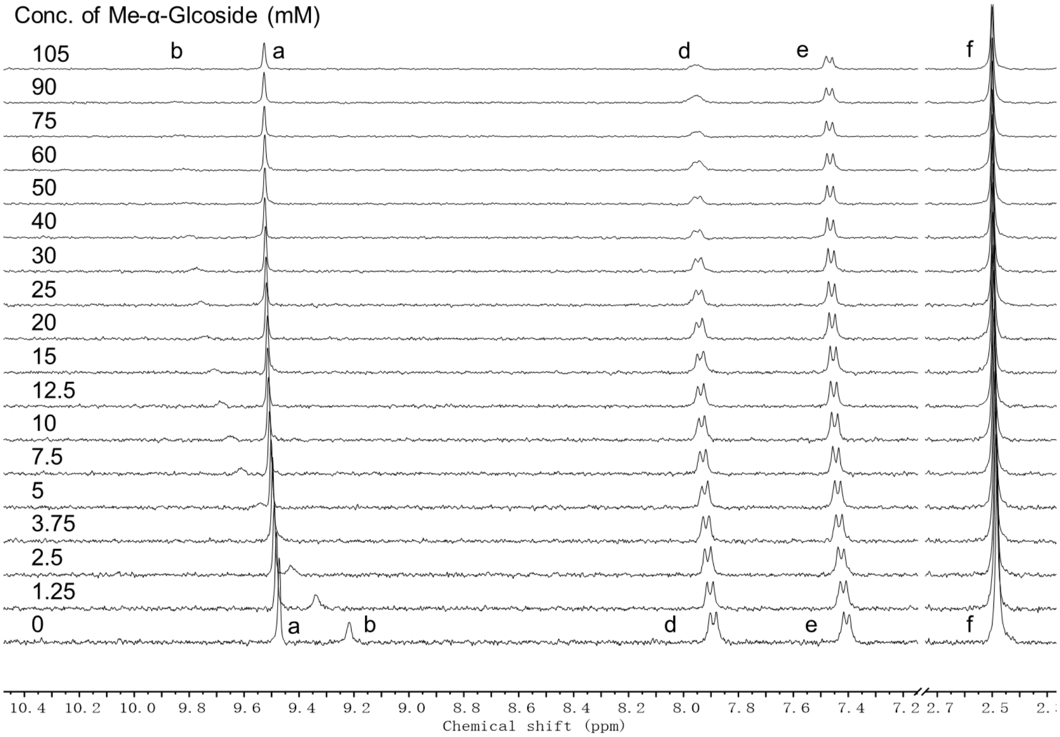

Figure S26. ^{1}H NMR spectra (400 MHz, D_2O, 298 K) of **$MPNT^{2+}$•$2Cl^-$** (0.15 mM) titrated with Methyl α-glucoside.

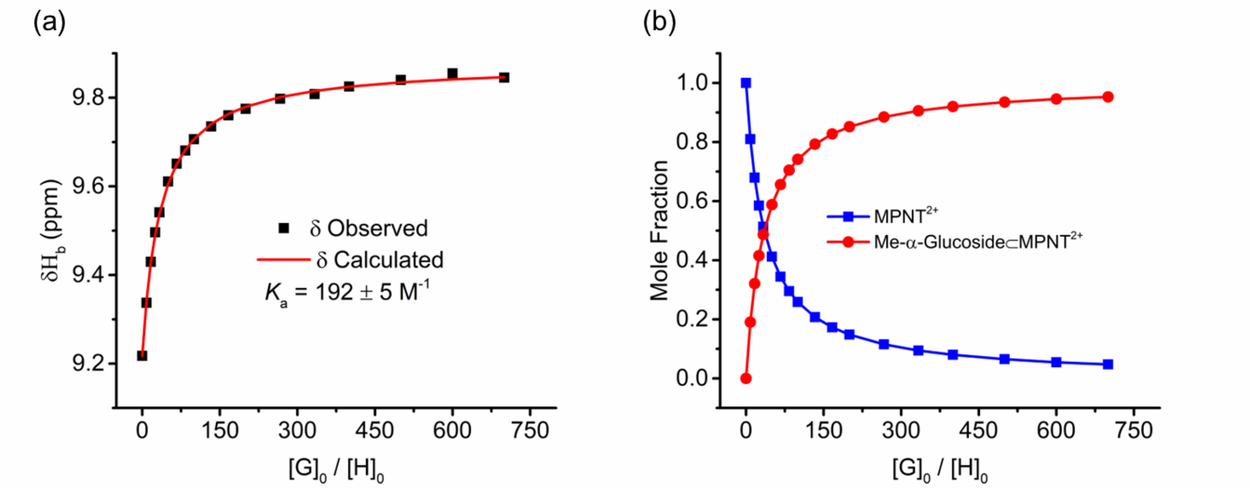

Figure S27. (a) Titration isotherm obtained by monitoring the chemical shift changes of proton b of $MPNT^{2+}$•$2Cl^-$ (0.15 mM) upon the incremental addition of Methyl α-glucoside in D_2O at 298 K. Red curves represent the best fit using a 1:1 host–guest binding model. (b) Calculated changes of mole fractions for $MPNT^{2+}$•$2Cl^-$ (blue trace) and Methyl α-glucoside⊂$MPNT^{2+}$ (red trace) over the host-guest mole ratio.

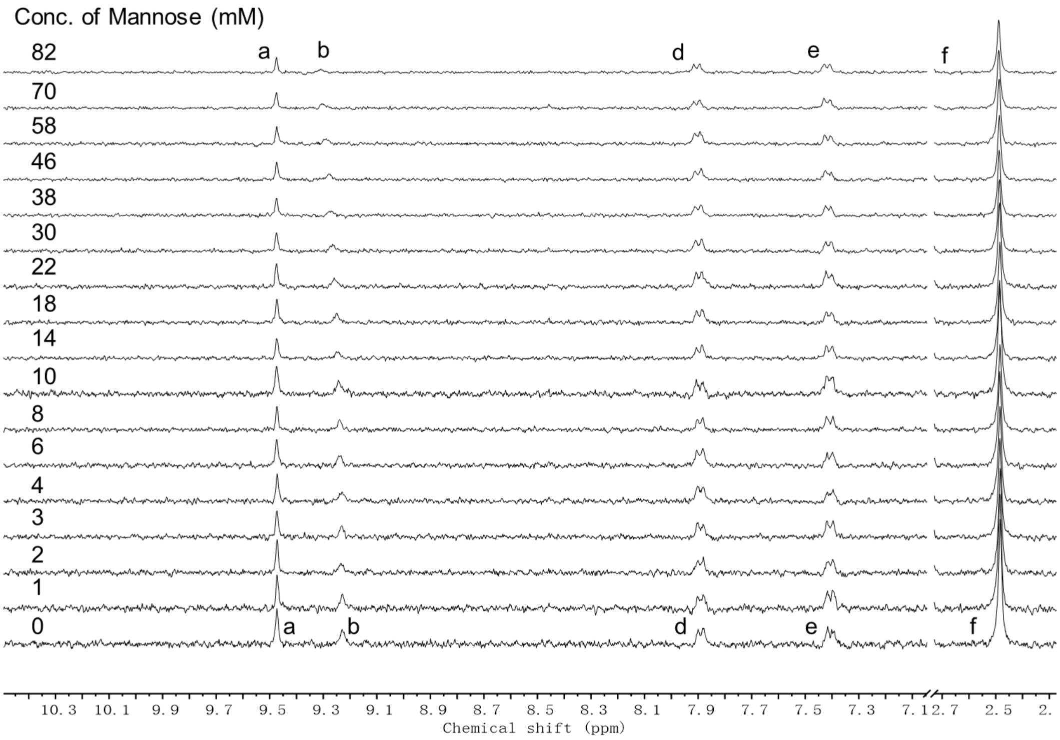

Figure S28. ^{1}H NMR spectra (400 MHz, D_2O, 298 K) of $MPNT^{2+}$•$2Cl^-$ (0.12 mM) titrated with mannose.

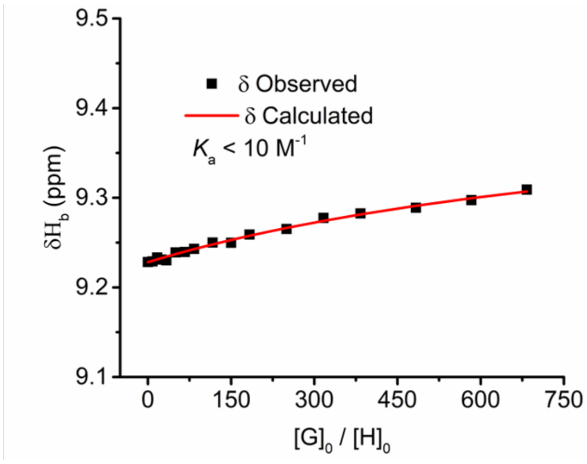

Figure S29. (a) Titration isotherm obtained by monitoring the chemical shift changes of proton b for $MPNT^{2+}$•$2Cl^{-}$ (0.15 mM) upon the incremental addition of mannose in D_2O at 298 K. Red curves represent the best fit using a 1:1 host–guest binding model.

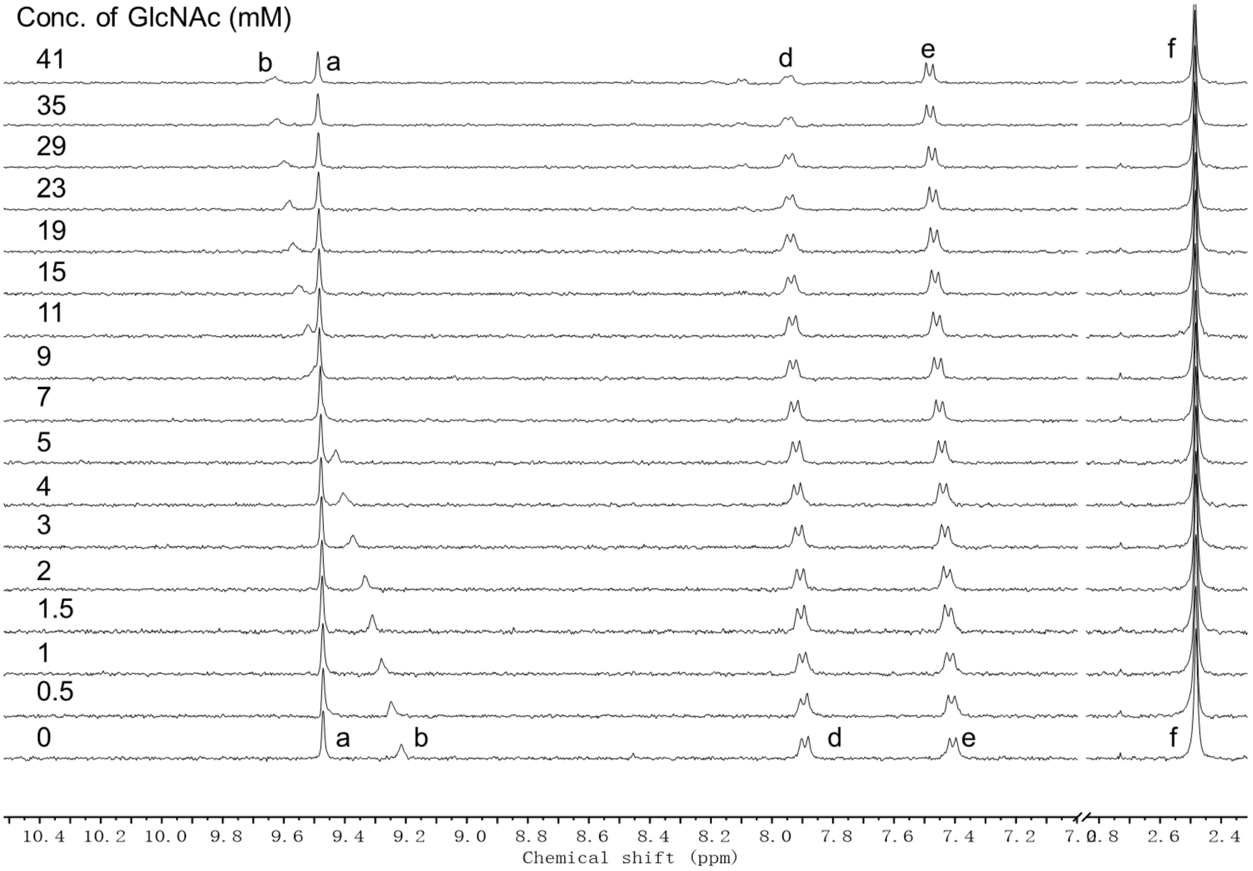

Figure S30. ^{1}H NMR spectra (400 MHz, D_2O, 298 K) of $MPNT^{2+}$•$2Cl^{-}$ (0.15 mM) titrated with GlcNAc.

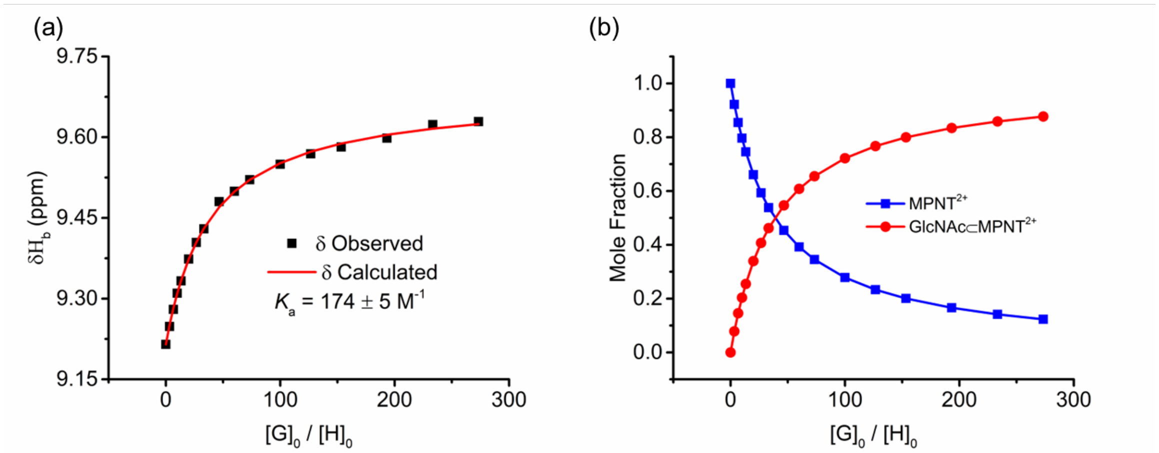

Figure S31. (a) Titration isotherm obtained by monitoring the chemical shift changes of proton b for $MPNT^{2+}$•$2Cl^{-}$ (0.15 mM) upon the incremental addition of GlcNAc in D_2O at 298 K. Red curves represent the best fit using a 1:1 host–guest binding model. (b) Calculated changes of mole fractions for $MPNT^{2+}$•$2Cl^{-}$ (blue trace) and GlcNAc⊂$MPNT^{2+}$ (red trace) over the host-guest mole ratio.

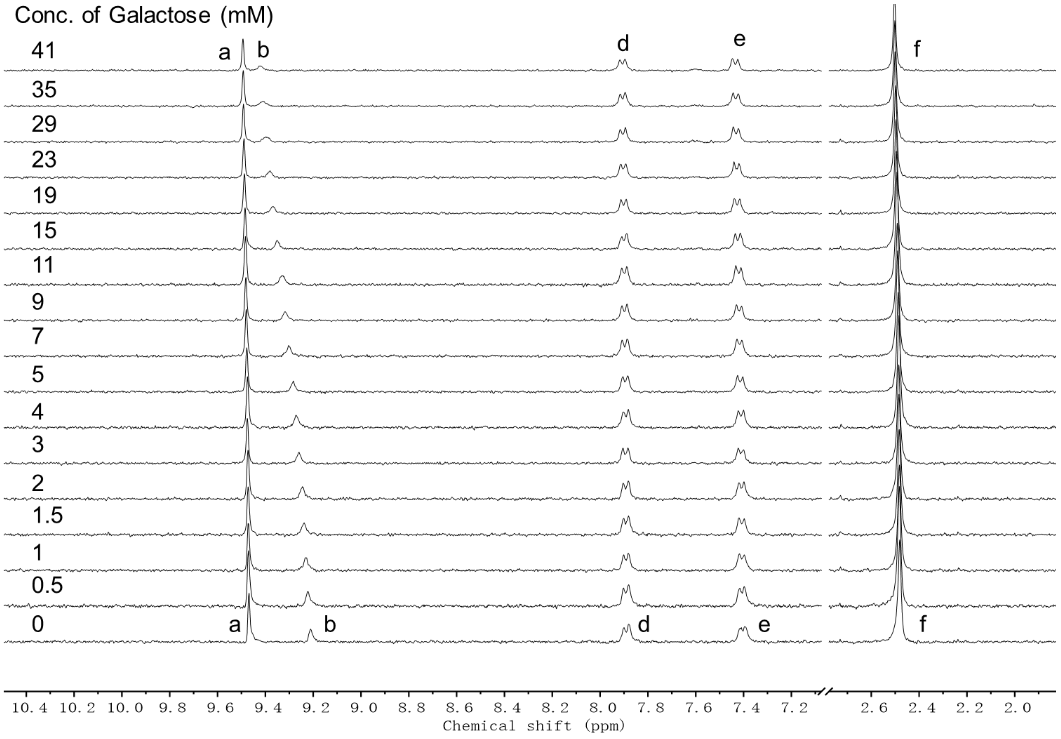

Figure S32. ^{1}H NMR spectra (400 MHz, D_2O, 298 K) of $MPNT^{2+}$•$2Cl^{-}$ (0.15 mM) titrated with galactose.

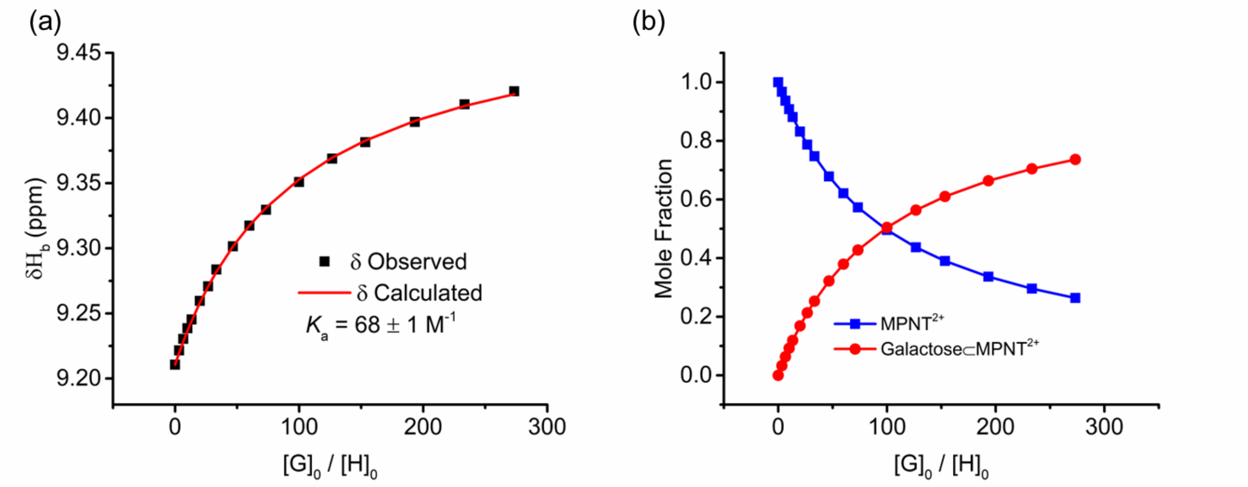

Figure S33. (a) Titration isotherm obtained by monitoring the chemical shift changes of proton b for $MPNT^{2+}•2Cl^{–}$ (0.15 mM) upon the incremental addition of galactose in D_2O at 298 K. Red curves represent the best fit using a 1:1 host–guest binding model. (b) Calculated changes of mole fractions for $MPNT^{2+}•2Cl^{–}$ (blue trace) and galactose⊂$MPNT^{2+}$ (red trace) over the host-guest mole ratio.

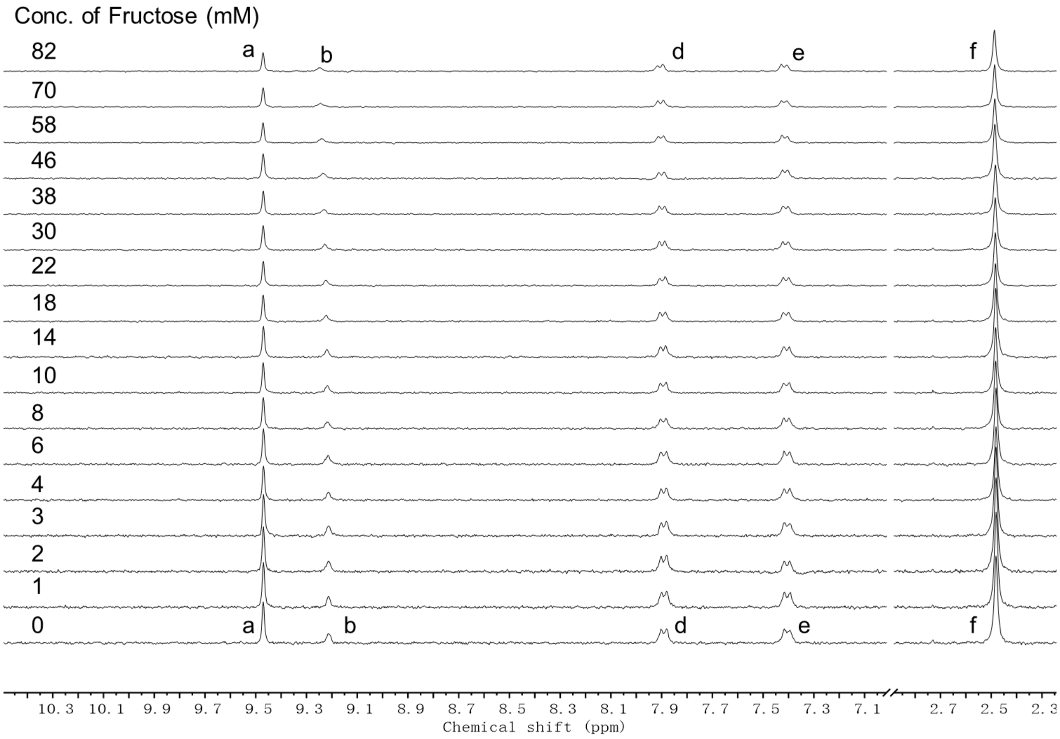

Figure S34. ^{1}H NMR spectra (400 MHz, D_2O, 298 K) of $MPNT^{2+}•2Cl^{–}$ (0.15 mM) titrated with fructose.

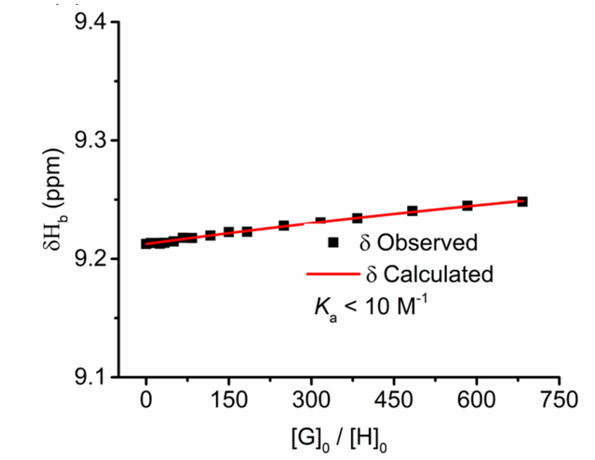

Figure S35. (a) Titration isotherm obtained by monitoring the chemical shift changes of proton b for MPNT^{2+}•2Cl^{-} (0.15 mM) upon the incremental addition of fructose in D_2O at 298 K. Red curves represent the best fit using a 1:1 host–guest binding model.

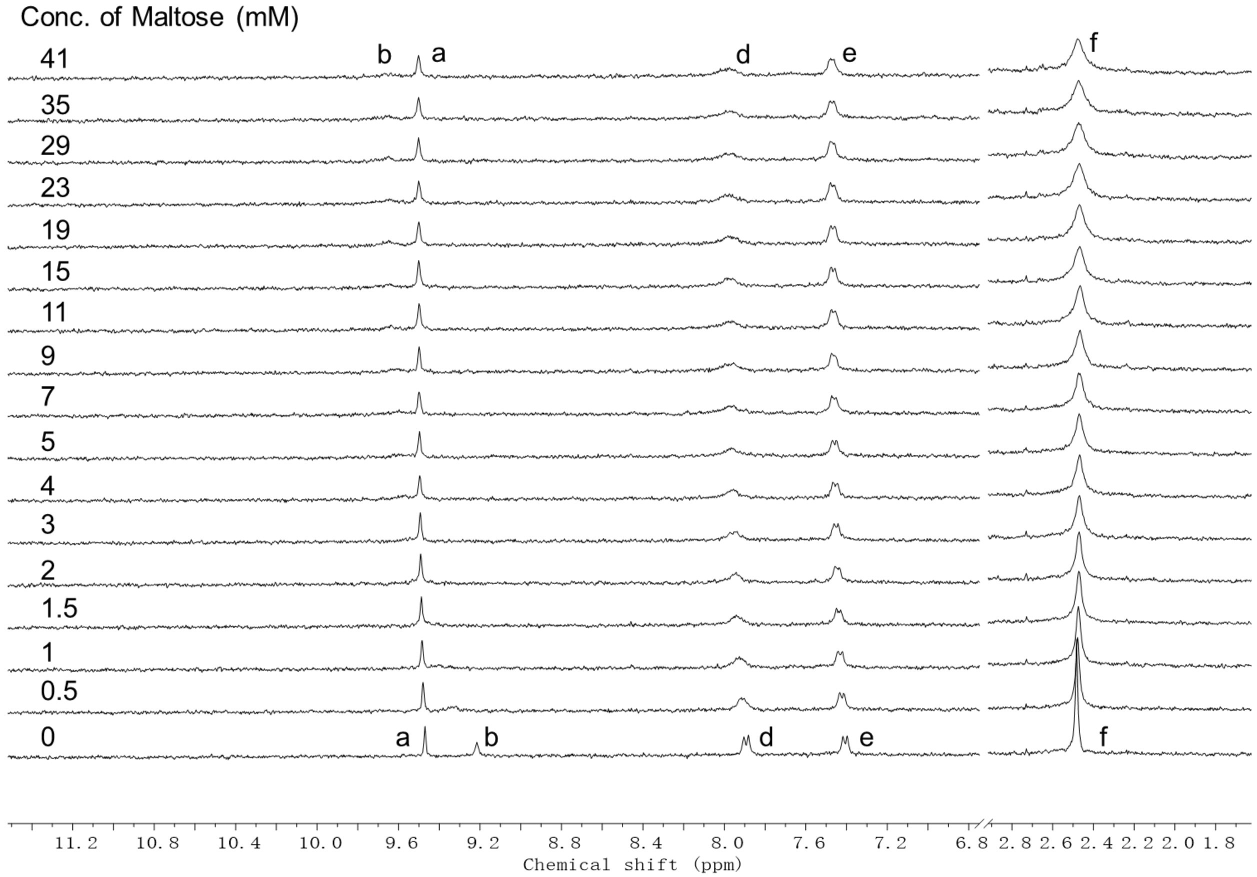

Figure S36. ^{1}H NMR spectra (400 MHz, D_2O, 298 K) of MPNT^{2+}•2Cl^{-} (0.15 mM) titrated with maltose.

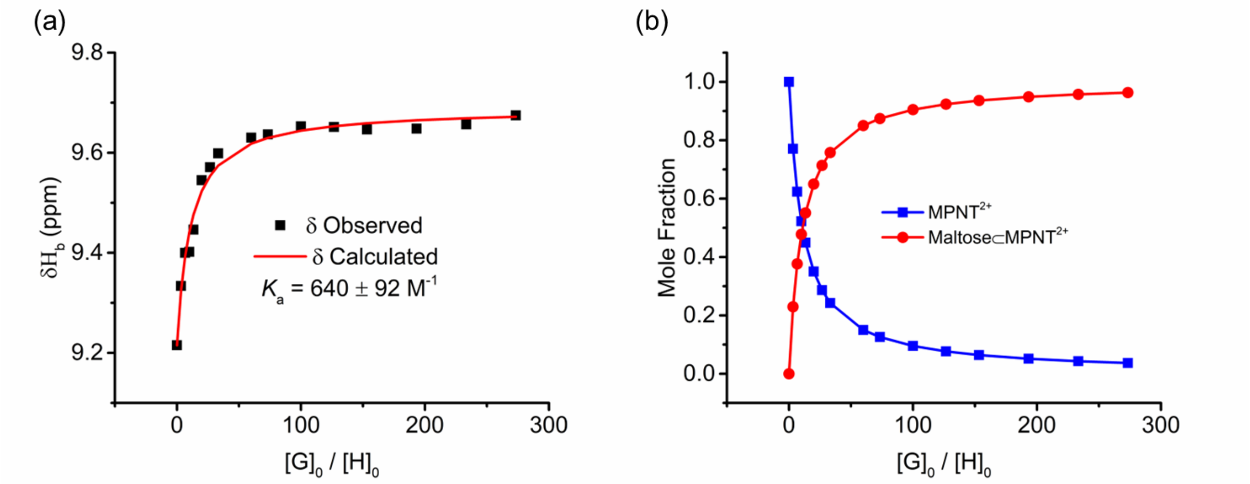

Figure S37. (a) Titration isotherm obtained by monitoring the chemical shift changes of proton b for $MPNT^{2+}$•$2Cl^{-}$ (0.15 mM) upon the incremental addition of maltose in D_2O at 298 K. Red curves represent the best fit using a 1:1 host–guest binding model. (b) Calculated changes of mole fractions for $MPNT^{2+}$•$2Cl^{-}$ (blue trace) and maltose⊂$MPNT^{2+}$ (red trace) over the host-guest mole ratio.

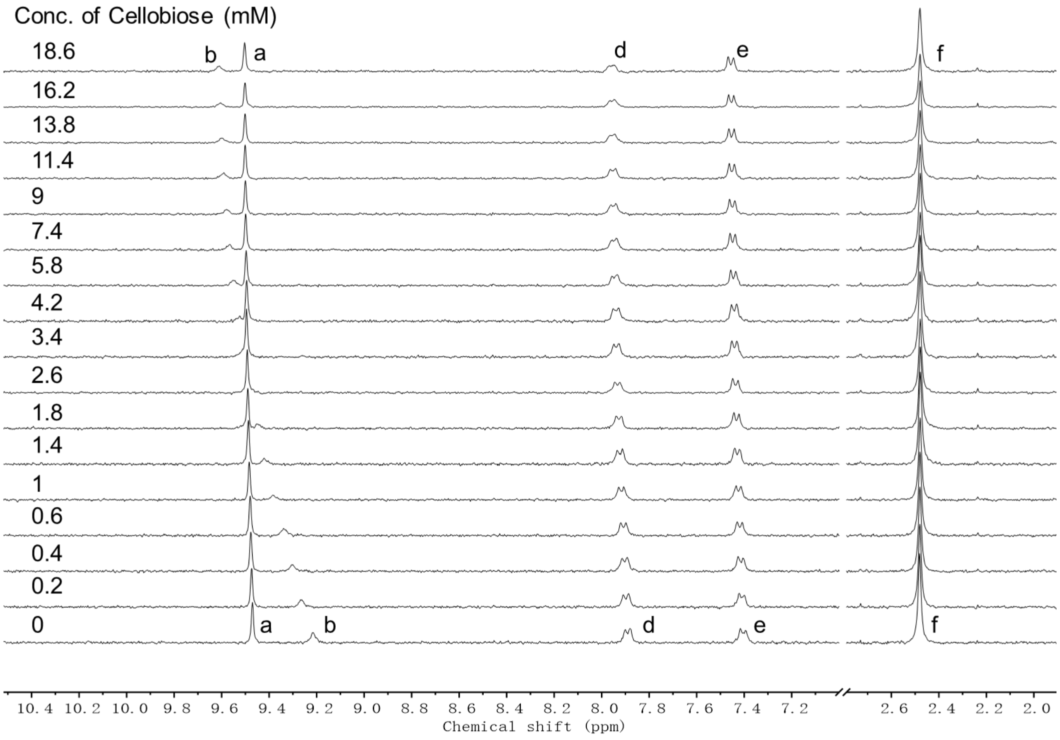

Figure S38. ^{1}H NMR spectra (400 MHz, D_2O, 298 K) of $MPNT^{2+}$•$2Cl^{-}$ (0.15 mM) titrated with cellobiose.

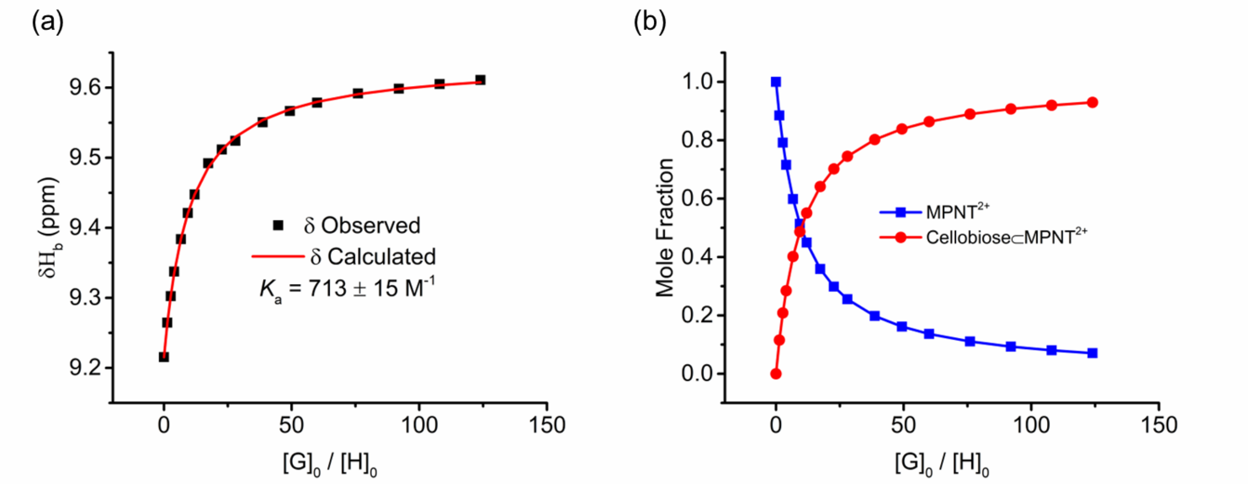

Figure S39. (a) Titration isotherm obtained by monitoring the chemical shift changes of proton b for $MPNT^{2+}•2Cl^{-}$ (0.15 mM) upon the incremental addition of cellobiose in D_2O at 298 K. Red curves represent the best fit using a 1:1 host–guest binding model. (b) Calculated changes of mole fractions for $MPNT^{2+}•2Cl^{-}$ (blue trace) and cellobiose⊂$MPNT^{2+}$ (red trace) over the host-guest mole ratio.

5. Isothermal Titration Calorimetry

TC experiments were carried out using a MicroCal ITC_{200} system at 23 °C. Titrations were performed in a 200 μL sample cell with a 40 μL injection syringe, under constant stirring at 750 rpm. All receptor and substrate solutions were prepared in Milli-Q water unless otherwise stated. A stock solution of $MPNT^{2+}·2Cl^{-}$ was prepared in Milli-Q water and filtered before use. The receptor solution ($MPNT^{2+}·2Cl^{-}$) was loaded into the sample cell, while the substrate solution was placed in the syringe. Each titration consisted of 20–25 injections. Control experiments were conducted by titrating the guest solution into Milli-Q water to measure the heat of dilution, which was subtracted from the raw data. The corrected data were analyzed using MicroCal ITC_{200} software with a 1:1 host–guest binding model and plotted using OriginLab software.

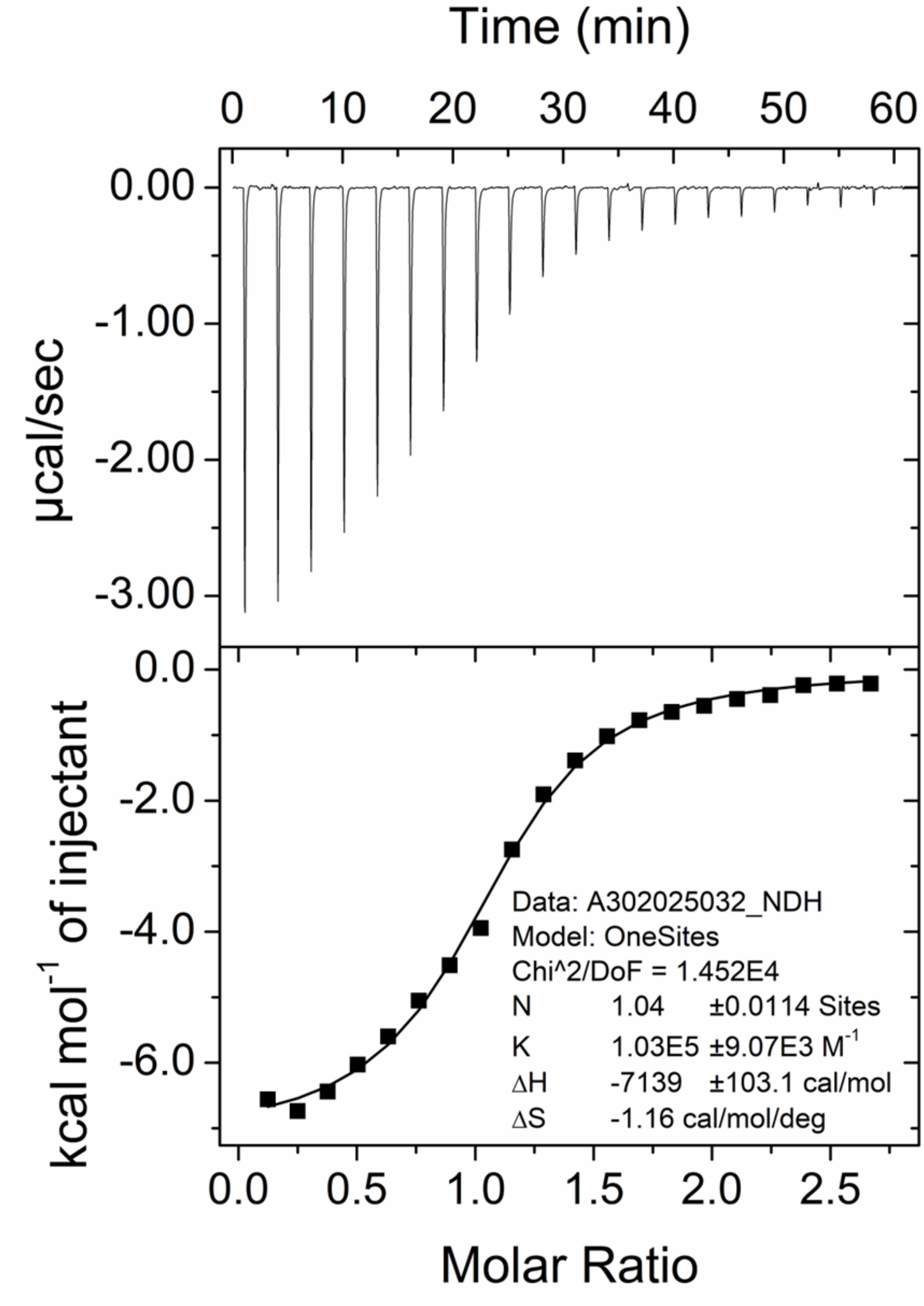

Figure S40. ITC profiles for the titration of **MPNT^{2+}•2Cl^{-}** (0.15 mM, in cell) with glucuronic acid (2.5 mM, in syringe) in H_2O at 23˚C. The solid line represents the best fit to a 1:1 binding model, yielding a binding constant $K_a = (1.03 \pm 0.09) \times 10^5$ M^{-1} and an enthalpy change ΔH = -7.14 ± 0.1 kcal/mol.

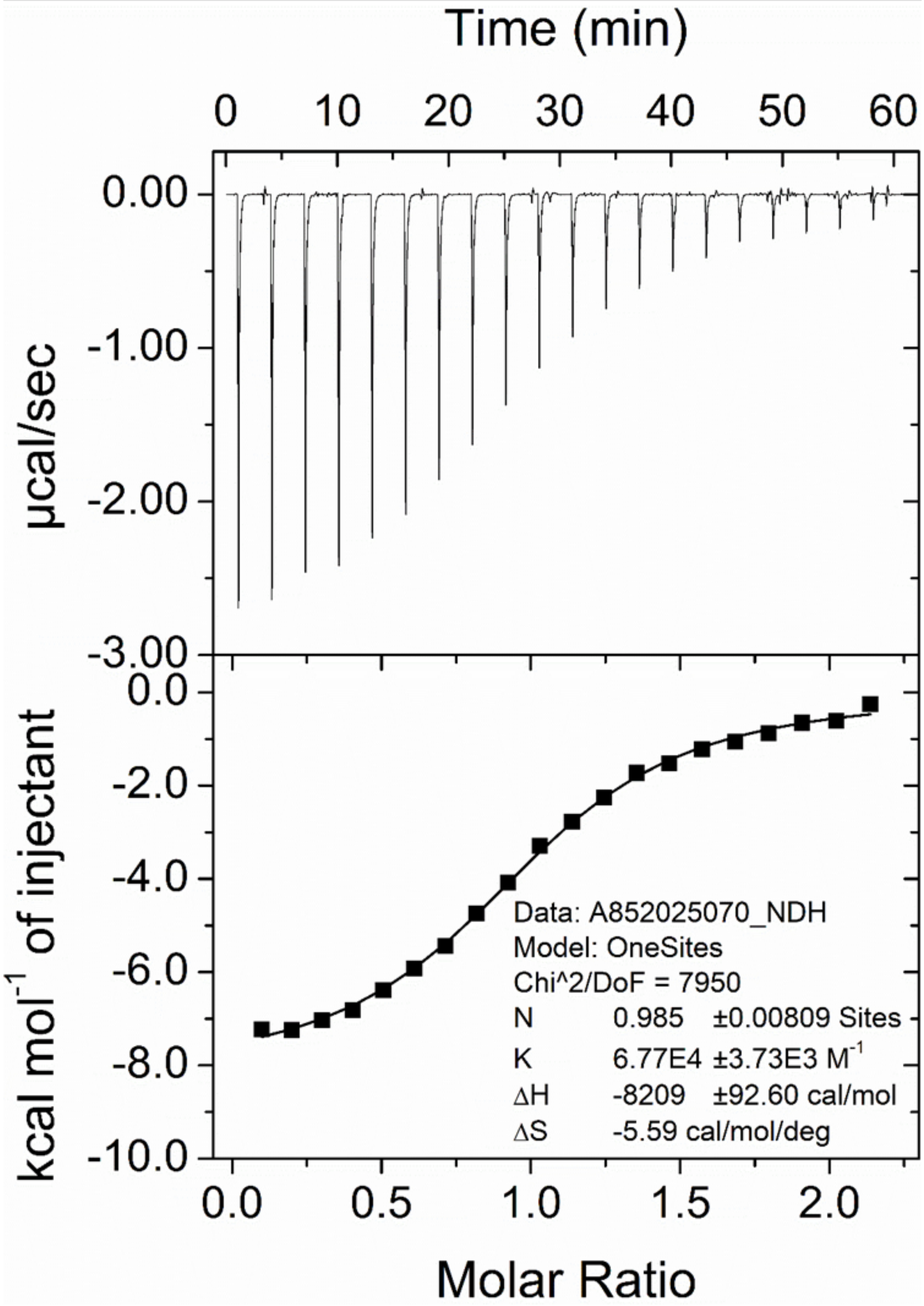

Figure S41. ITC profiles for the titration of **MPNT^{2+}•2Cl^{-}** (0.15 mM, in cell) with sodium glucuronate (2 mM, in syringe) in water at 23˚C. The solid line represents the best fit to a 1:1 binding model, yielding a binding constant K_a = (6.8 ± 0.4) × 10^4 M^{-1} and an enthalpy change ΔH = -8.2 ± 0.1 kcal/mol.

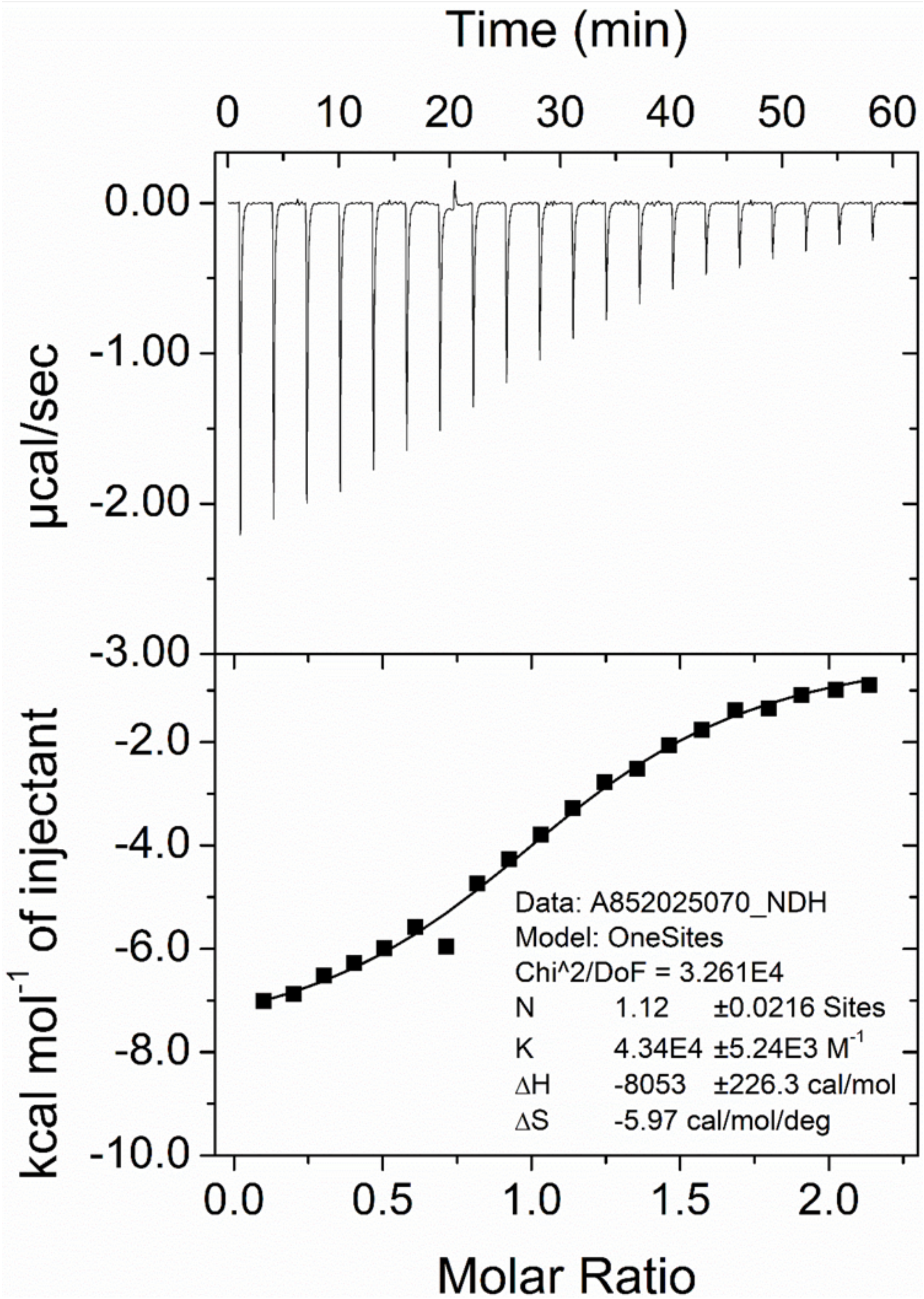

Figure S42. ITC profiles for the titration of **$MPNT^{2+}$•2Cl^-** (0.15 mM, in cell) with glucuronic acid (2 mM, in syringe) in 10 mM MES buffer (pH 6.5) at 23˚C. The solid line represents the best fit to a 1:1 binding model, yielding a binding constant K_a = (4.3 ± 0.5) × 10^4 M^{-1} and an enthalpy change ΔH = -8.1 ± 0.23 kcal/mol.

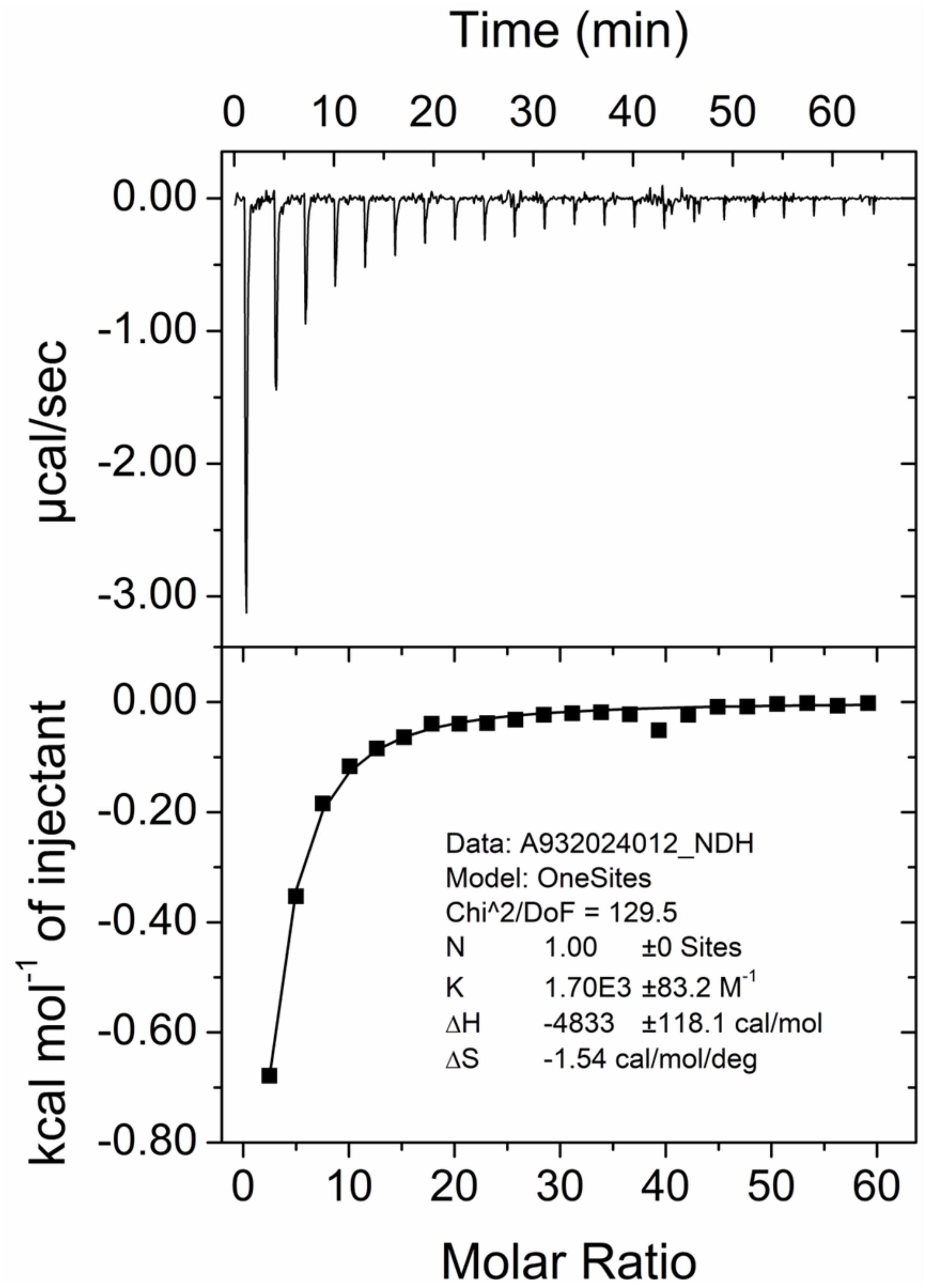

Figure S43. ITC profiles for the titration of $MPNT^{2+}$•$2Cl^-$ (0.15 mM, in cell) with glucose (50 mM, in syringe) in H_2O at 23 ˚C. The solid line represents the best fit to a 1:1 binding model, yielding a binding constant K_a = 1700 ± 83 M^{-1} and an enthalpy change ΔH = -4.83 ± 0.12 kcal/mol.

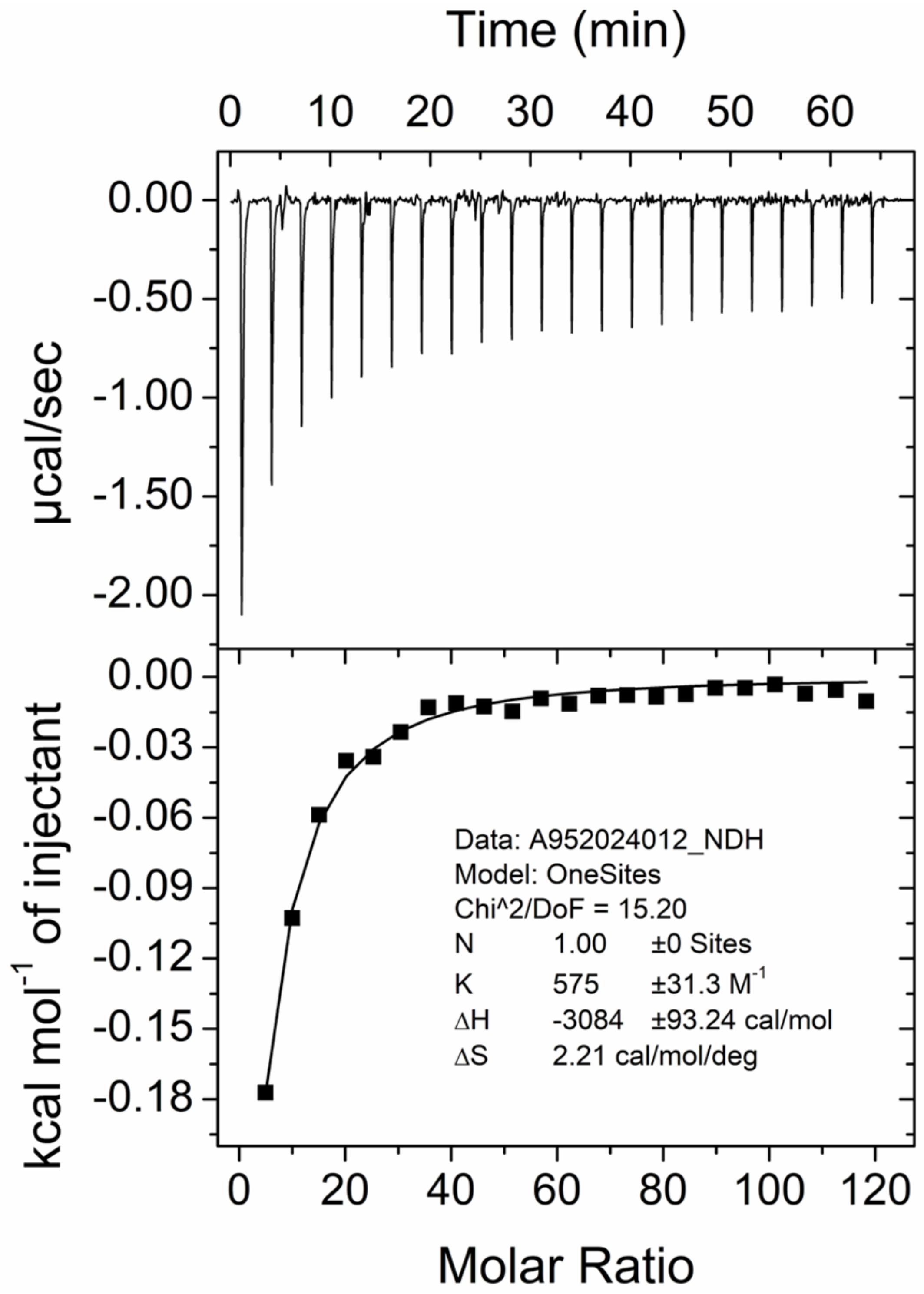

Figure S44. ITC profiles for the titration of MPNT^{2+}•2Cl^{-} (0.15 mM, in cell) with Methyl β-glucoside (100 mM, in syringe) in H_2O at 23 ˚C. The solid line represents the best fit to a 1:1 binding model, yielding a binding constant K_a = 575 ± 31 M^{-1} and an enthalpy change ΔH = -3.08 ± 0.09 kcal/mol.

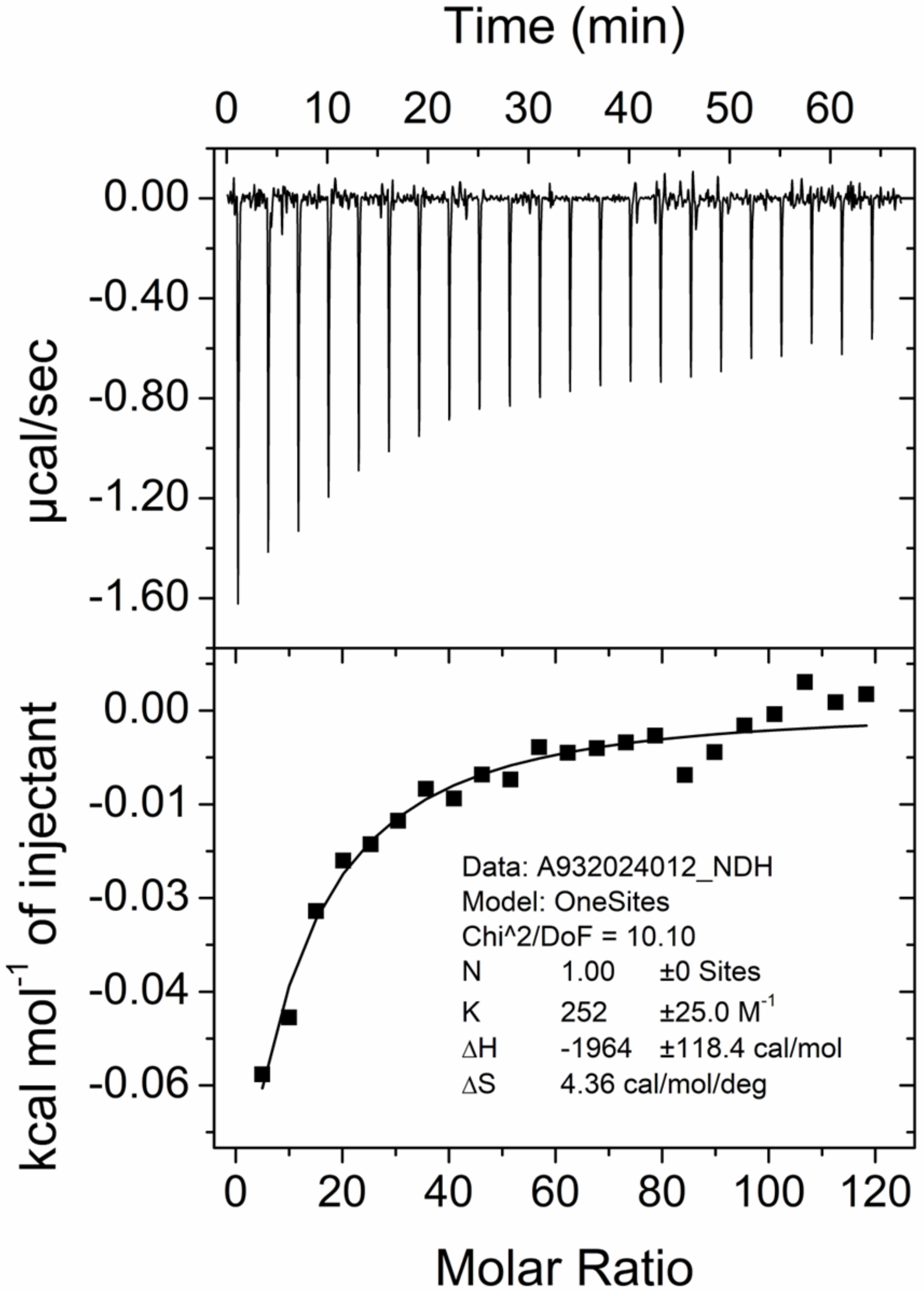

Figure S45. ITC profiles for the titration of $MPNT^{2+}$•$2Cl^{–}$ (0.15 mM, in cell) with Methyl α-glucoside (100 mM, in syringe) in H_2O at 23 ˚C. The solid line represents the best fit to a 1:1 binding model, yielding a binding constant K_a = 252 ± 25 M^{-1} and an enthalpy change ΔH = -1.96 ± 0.12 kcal/mol.

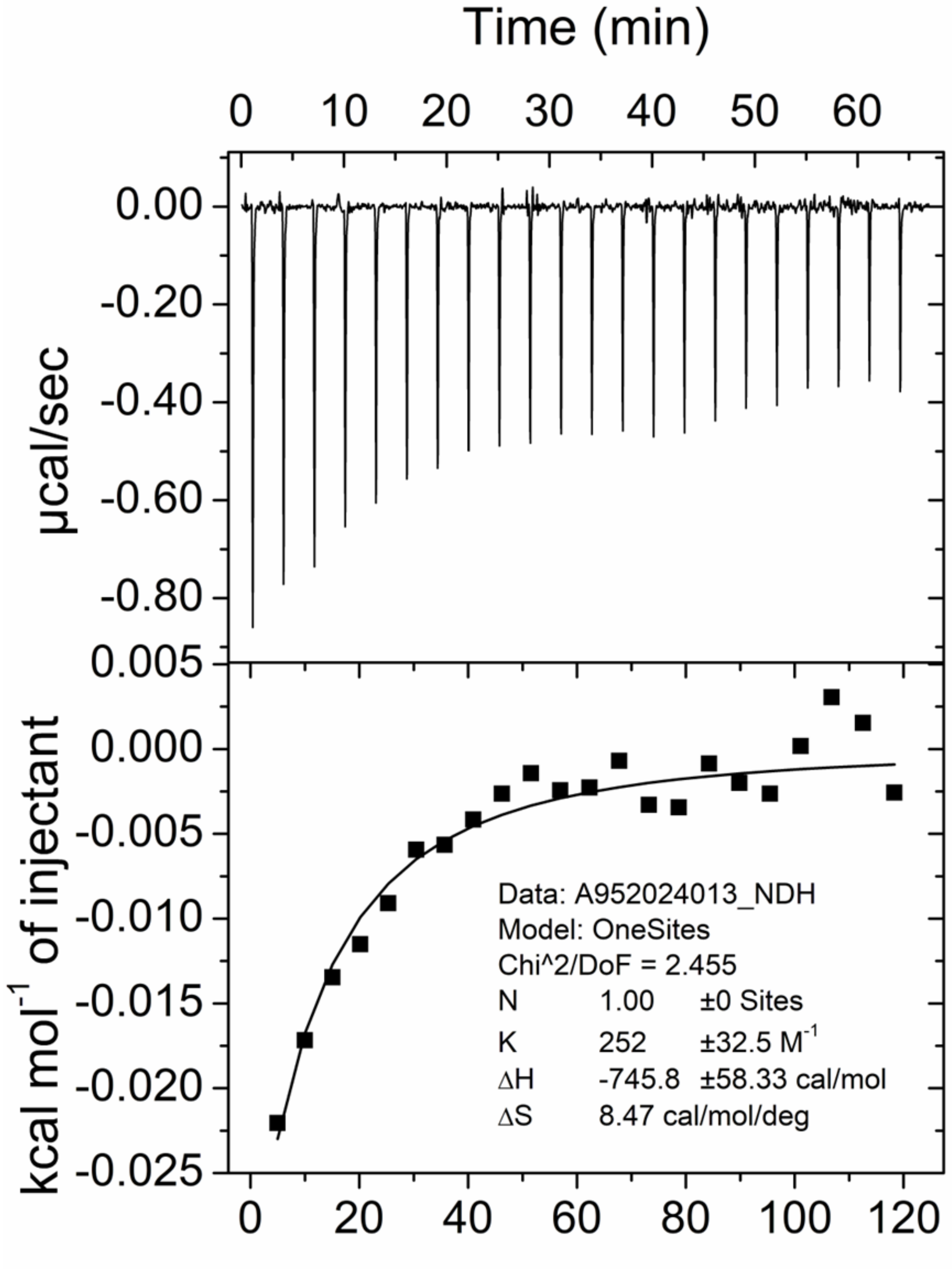

Figure S46. ITC profiles for the titration of $MPNT^{2+}$•$2Cl^{-}$ (0.15 mM, in cell) with GlcNAc (100 mM, in syringe) in H_2O at 23 ˚C. The solid line represents the best fit to a 1:1 binding model, yielding a binding constant K_a = 252 ± 33 M^{-1} and an enthalpy change ΔH = -0.75 ± 0.06 kcal/mol.

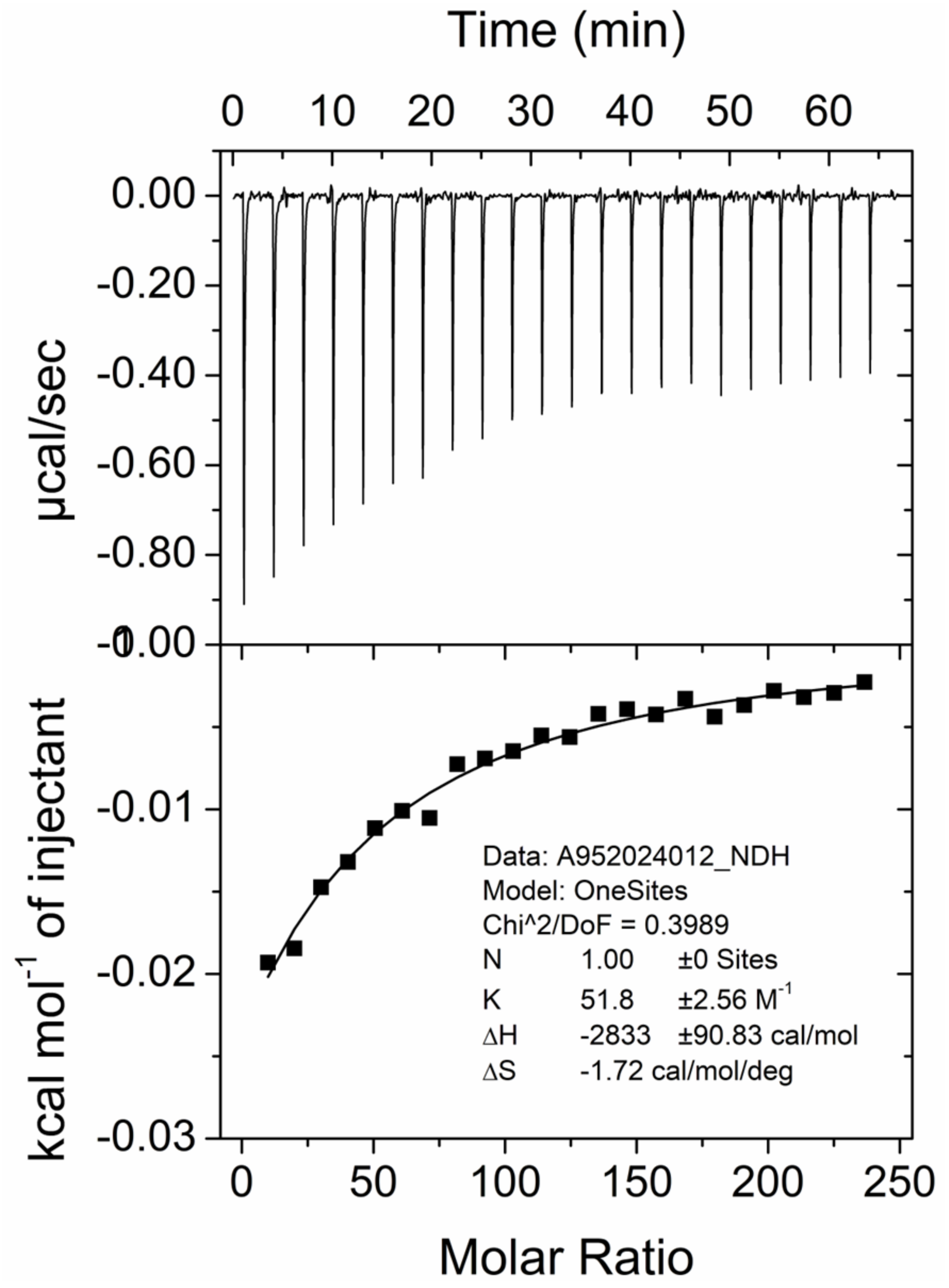

Figure S47. ITC profiles for the titration of $MPNT^{2+}$•$2Cl^{-}$ (0.15 mM, in cell) with galactose (200 mM, in syringe) in H_2O at 23 ˚C. The solid line represents the fit to a 1:1 binding model, yielding a binding constant K_a = 52 ± 2.6 M^{-1} and an enthalpy change ΔH = -2.8 ± 0.09 kcal/mol.

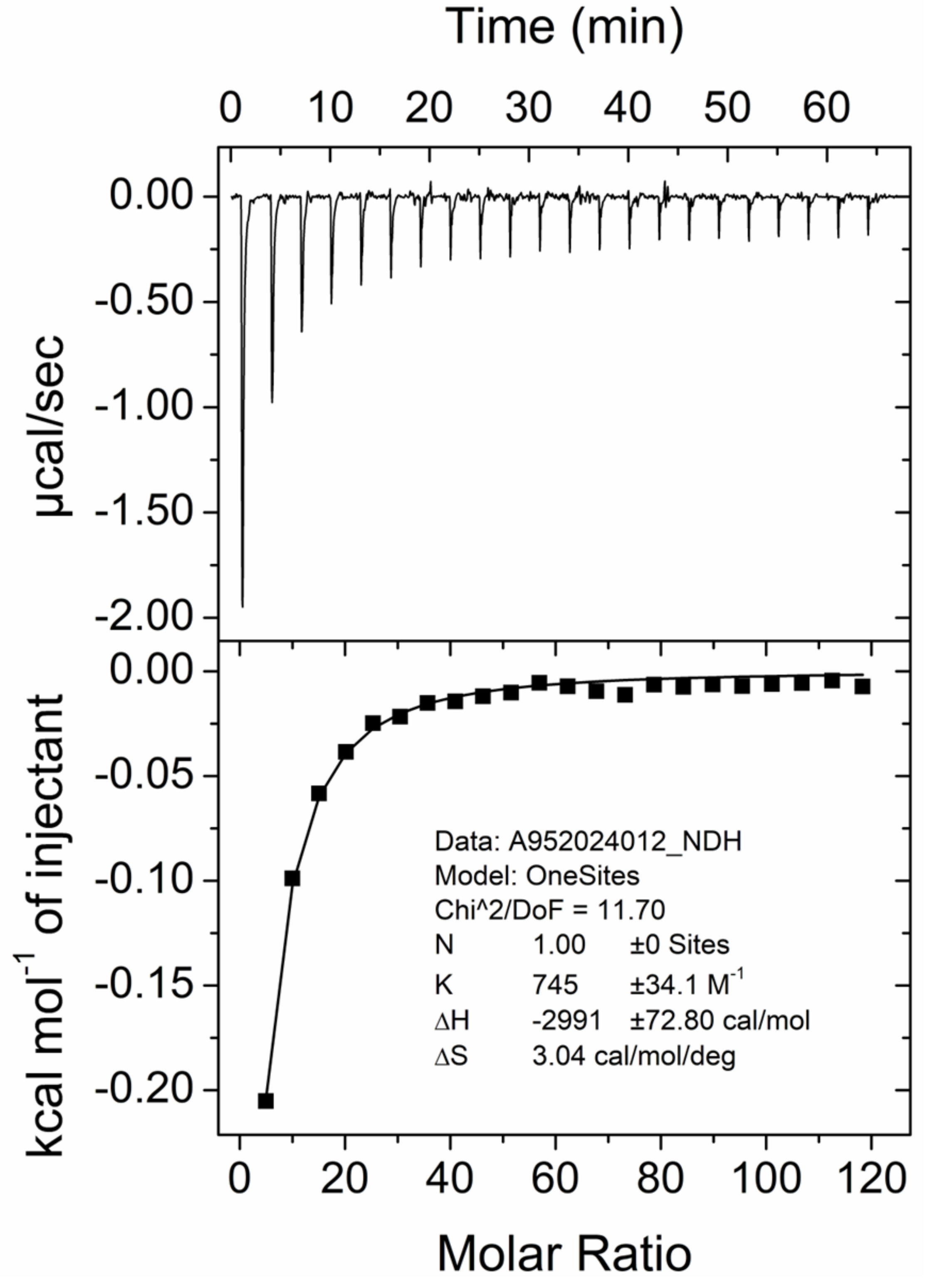

Figure S48. ITC profiles for the titration of $MPNT^{2+}$•$2Cl^{-}$ (0.15 mM, in cell) with maltose (50 mM, in syringe) in H_2O at 23 °C. The solid line represents the best fit to a 1:1 binding model, yielding a binding constant K_a = 745 ± 34 M^{-1} and an enthalpy change ΔH = -2.99 ± 0.07 kcal/mol.

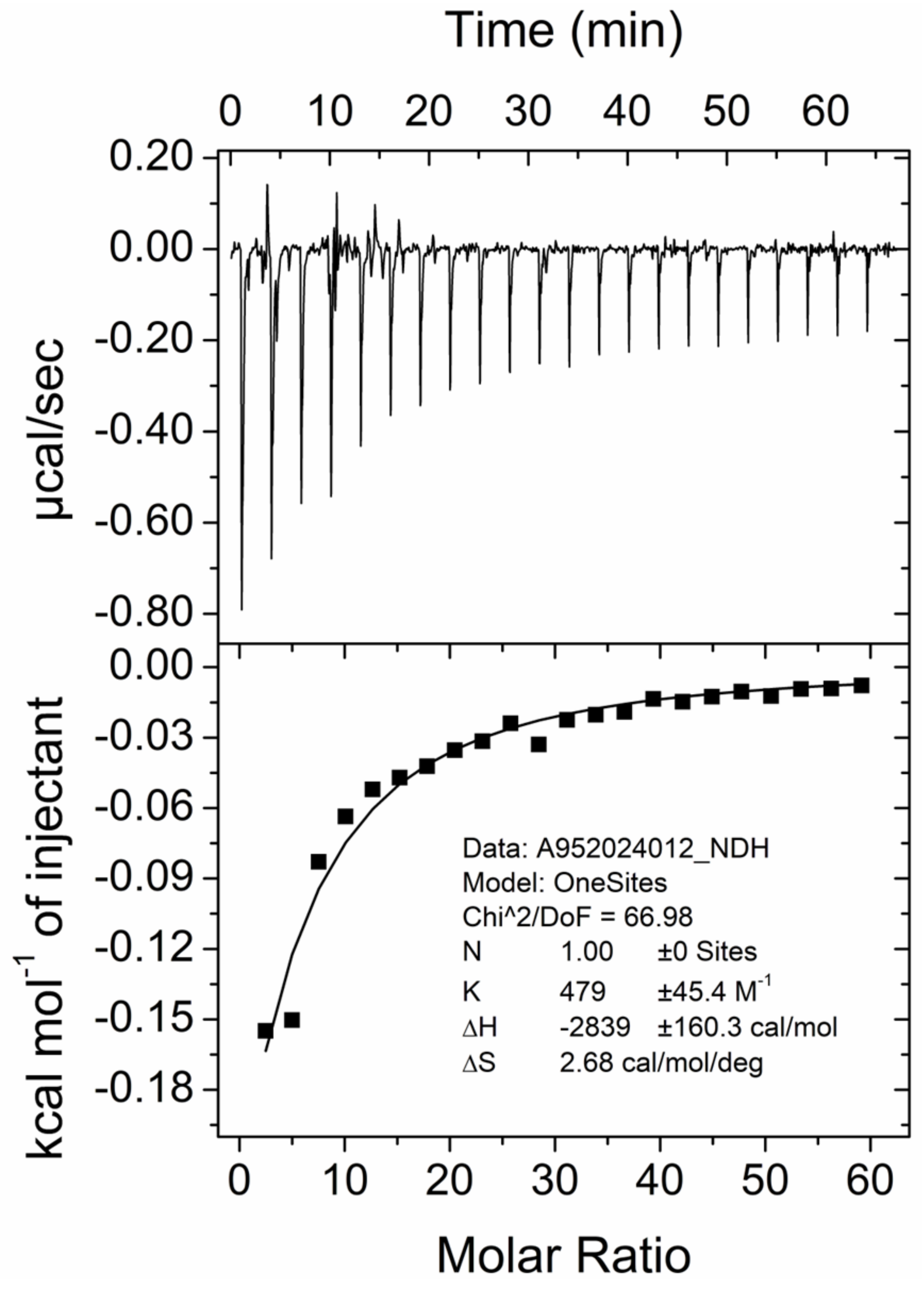

Figure S49. ITC profiles for the titration of $MPNT^{2+}$•$2Cl^{-}$ (0.15 mM, in cell) with cellobiose (50 mM, in syringe) in H_2O at 23 ˚C. The solid line represents the best fit to a 1:1 binding model, yielding a binding constant K_a = 479 ± 45 M^{-1} and an enthalpy change ΔH = -2.8 ± 0.16 kcal/mol.

6. UV/Vis Absorption and Fluorescence Emission Spectroscopy

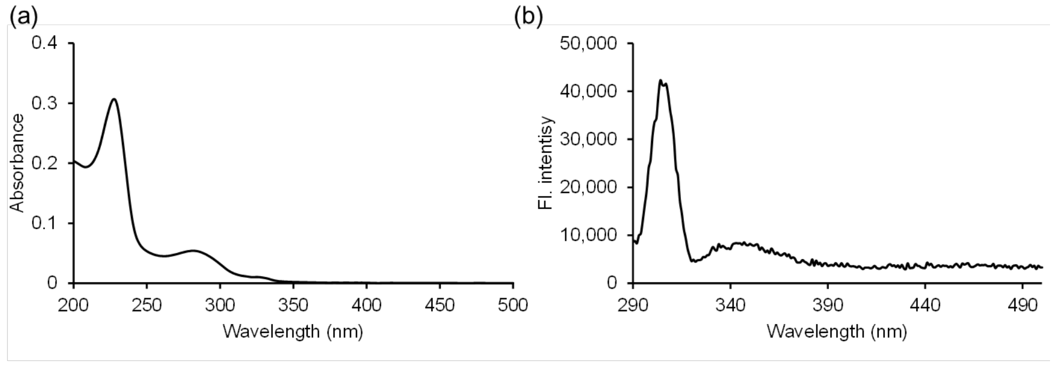

Figure S50. (a) UV/Vis absorption and (b) fluorescence emission (λ_{ex} = 276 nm, slit width 8 nm) spectra of $MPNT^{2+}$•$2Cl^{-}$ (1.6 μM) in water.

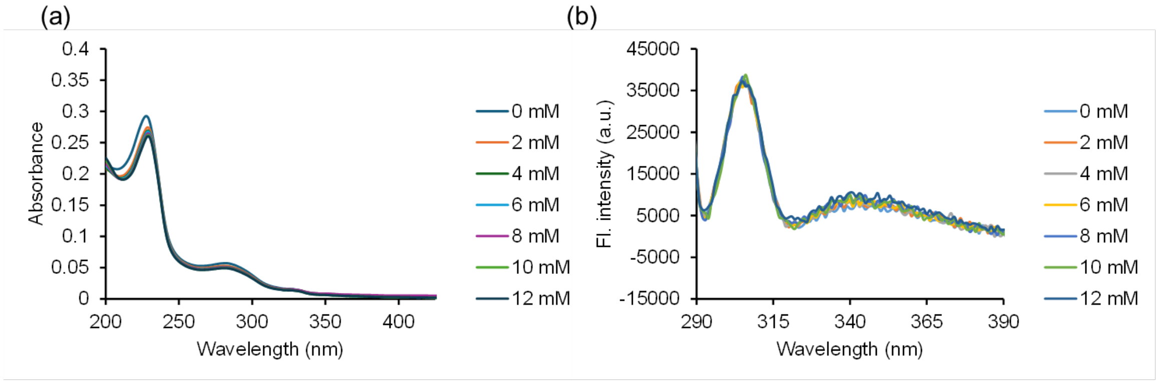

Figure S51. (a) UV/Vis absorption and (b) fluorescence emission (λ_{ex} = 276 nm, slit width 8 nm) spectra of $MPNT^{2+}$•$2Cl^{-}$ (1.6 μM) titrated with glucose in water.

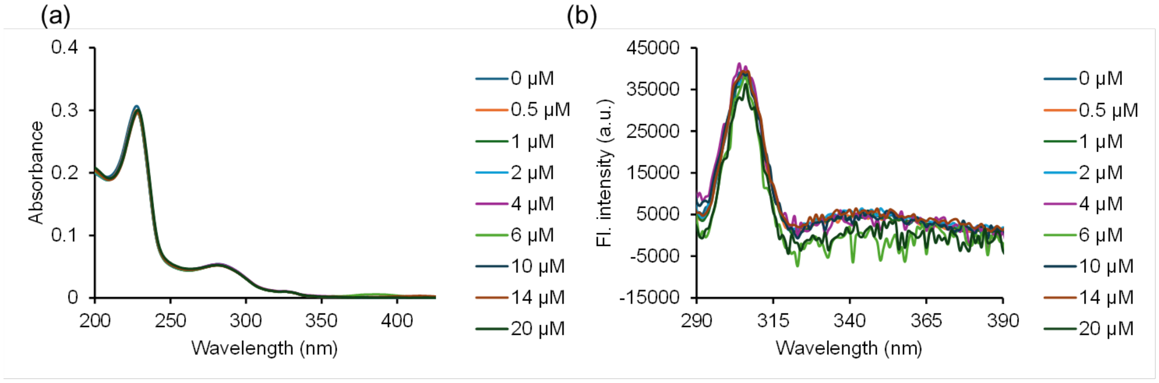

Figure S52. (a) UV/Vis absorption and (b) fluorescence emission (λ_{ex} = 276 nm, slit width 8 nm) spectra of sample $MPNT^{2+}$•$2Cl^{-}$ (1.6 μM) titrated with glucuronic acid in water.

7. Circular Dichroism Spectroscopy

Circular dichroism spectra were collected using the following acquisition parameters:

Measure Range	300 – 190 nm
Data pitch	0.5 nm
Data points	221
D.I.T.	0.5 sec
Bandwidth	1 nm
Scanning speed	200 nm/min
Shutter control	Auto
Accumulations	15
Solvent	DI. water
Cell length	1 cm
Temperature	25 °C

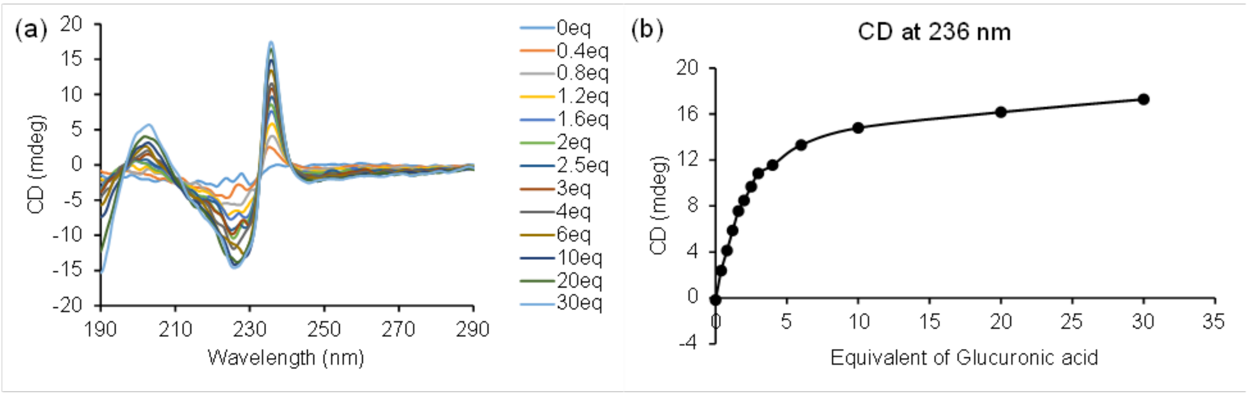

Figure S53. (a) CD spectra of $MPNT^{2+}$•$2Cl^{-}$ (10 μM) titration with glucuronic acid (0-300 μM) in water. (b) Change of CD signal at 236 nm over the incremental addition of glucuronic acid.

8. X-Ray Crystallography Data and Analysis

A single crystal of the glucuronate⊂$MPNT^{2+}$ complex was obtained by slow evaporation of a solution containing $MPNT^{2+}\cdot 2Cl^{-}$ (0.45 mM) and glucuronic acid (3 mM) in water. X-ray diffraction data were measured on Bruker D8 Venture PHOTON III CMOS diffractometer equipped with a Cu Kα INCOATEC ImuS micro-focus source (λ = 1.54178 Å). Indexing was performed using[S5] APEX4 (Difference Vectors method). Data integration and reduction were performed using[S6] SaintPlus. Absorption correction was performed[S7] by multi-scan method implemented in SADABS. Space group was determined using XPREP implemented in APEX3. Structure was solved using[S8] SHELXT and refined using[S9] SHELXL-2019/1 (full-matrix least-squares on F2) through OLEX2 interface program[S10]. The ellipsoid plot was made with Olex2. Crystals were very thin plates and did not diffract past approximately 1Å resolution. The model was refined with global SIMU / RIGU restraints. Organic molecules were refined with SAME restraints. The geometry of water molecules was refined using DFIX/DANG restraints. Hydrogen atoms of water molecules were modeled based on presence of hydrogen bond network and were refined using $H\ldots O_{(accpetor)}$ distance restraints determined using CSD database search[S11]. It was not possible to calculate some of hydrogen atoms due to heavy disorder. It was assumed that glucuronic acid is deprotonated based on available evidence (no electron density present, shortened C-O bond distances of 1.24Å and 1.28Å, pKa ~3 value and the lack of clear evidence where two extra Cl^{-} anions could be located). The structure is pseudo-centrosymmetric. The true symmetry is P1 as enantiopure glucuronic acid was used in synthesis. Data and refinement conditions are shown in Table S1.

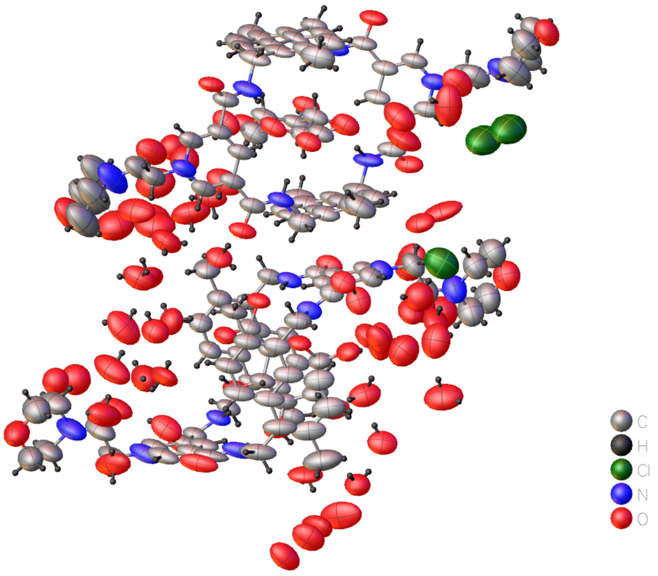

Figure S54. Ellipsoid plot of glucuronate ⊂$MPNT^{2+}\bullet Cl^{-}$. Anisotropic displacement parameters were drawn at 50% probability level.

Table S1 Crystal data and structure refinement for MPNT (CCDC: 2451974).	
Identification code	MPNT
Empirical formula	$C_{60}H_{93.73}ClN_8O_{33.91}$
Moiety formula	$C_{54}H_{58}N_8O_8$, $C_6H_9O_7$, Cl, 13.37(H_2O), 5.54$(O)_{H2O}$
Formula weight	1505.09
Temperature/K	145.00
Crystal system	triclinic
Space group	P1
a/Å	13.5765(3)
b/Å	17.2163(5)
c/Å	17.8948(4)
α/°	66.847(2)
β/°	89.697(2)
γ/°	81.427(2)
Volume/$Å^3$	3796.28(17)
Z	2
ρ_{calc}g/cm^3	1.317
μ/mm^{-1}	1.232
F(000)	1596.0
Crystal size/mm^3	0.15 × 0.08 × 0.02
Radiation	CuKα (λ = 1.54178)
2Θ range for data collection/°	5.38 to 99.5
Index ranges	-13 ≤ h ≤ 13, -17 ≤ k ≤ 17, -17 ≤ l ≤ 17
Reflections collected	41839
Independent reflections	15011 [R_{int} = 0.1143, R_{sigma} = 0.1159]
Data/restraints/parameters	15011/3260/2149
Goodness-of-fit on F^2	1.029
Final R indexes [I>=2σ (I)]	R_1 = 0.0953, wR_2 = 0.2540
Final R indexes [all data]	R_1 = 0.1466, wR_2 = 0.3034
Largest diff. peak/hole / e Å^{-3}	0.34/-0.23

9. Computational Analysis

Structural optimization and ESP calculation: The XYZ coordinates for the calculations were extracted from the X-ray single-crystal data. Structural optimizations of hydrogen atoms were performed with density functional theory (DFT) in the Orca program[S12] (version 5.0.3) using the Becke '88 exchange and Lee-Yang-Parr correlation (BLYP) functional[S13], the Ahlrich's double zeta Def2-SVP basis sets[S14] with geometrical counterpoise (gCP) scheme[S15], and Grimme's third-generation dispersion correction[S16] with Becke-Johnson damping (D3BJ). To speed up the DFT optimizations, the Coulomb integral[S17] and numerical chain-of-sphere integration[S18] for the HF exchanges (RIJCOSX) method was applied with the Def2/J auxiliary basis (AuxJ) [S19]. All optimizations were performed in a water continuum with the Conductor-like Polarizable Continuum Model (CPCM) in Orca. Frequency calculations of the resulting optimized structures reveal no imaginary frequency, suggesting the optimized structures were in local energy minima. The resulting wavefunction files for the optimized structures were used as input for calculating electron density and total electrostatic potential using the Multiwfn 3.6 program[S20] based on computerized optimized code method[S21, S22]. The resulting data were visualized using Chimera software to generate the electrostatic potential map (ESP) of the complex.[S23] To better visualize the electrostatic attractions between the the substrate and the receptor, the total electrostatic potentials of the macrocycle and glucuronic acid were calculated separately using the optimized coordinates of the complex.

IGMH analysis IBSIW calculation: Independent Gradient Model based on Hirshfeld partition (IGMH) analysis[S24, S25] is an approach to identifying and visualizing intermolecular interactions. Strong polar attractions and weak van der Waals contacts are visualized as an isosurface with blue and green colors. The binding surface was calculated by the Multiwfn 3.8 program[S20] through function 20 (visual study of weak interaction) and visualized by Chimera software. Intrinsic bond strength index for weak interactions[S25–S27] (IBSIW) was calculated by the Multiwfn 3.8 program as an approach to quantify the strength of individual hydrogen bonds between the receptor and glucuronic acid.

CREST sampling of cavity water: The coordinates of $MPNT^{2+}$ were obtained from its single-crystal structure, and a $(H_2O)_{30}$ cluster was positioned around its binding pocket to model the hydration environment. This hydrated complex was initially optimized using the semi-empirical

GFN2-xTB method.[S28] The resulting structure was then subjected to conformational sampling using the CREST program with the NCI model to explore potential configurations of water molecules within the macrocyclic cavity.[S29–S31] For comparison, a separate CREST sampling was performed on a $(H_2O)_{30}$ cluster in the absence of $MPNT^{2+}$ to represent bulk water structures.

Four low-energy conformers (within a 6 kcal·mol^{-1} energy window) were identified for the bulk $(H_2O)_{30}$ cluster (Figure S55), with an average of 46.5 hydrogen bonds—equivalent to approximately 1.6 hydrogen bonds per water molecule. In contrast, three low-energy conformers (also within 6 kcal·mol^{-1}) were found for the $(H_2O)_{30}$ cluster in the presence of $MPNT^{2+}$ (Figure S56), averaging 45.3 hydrogen bonds, or about 1.5 hydrogen bonds per water molecule.

The similarity in hydrogen bonding patterns suggests that water molecules within the $MPNT^{2+}$ binding cavity maintain a hydrogen-bonding network comparable to that of bulk water. This contrasts with conventional macrocycles and cyclophanes, where cavity-bound water typically exhibits significantly fewer hydrogen bonds and is often classified as "high-energy water."[S32] Such high-energy water, upon displacement, contributes favorably to binding enthalpy. However, in the case of $MPNT^{2+}$, no such enthalpically destabilized water species were observed, indicating that displacement of cavity water is unlikely to provide a significant enthalpic driving force for guest binding in this system.

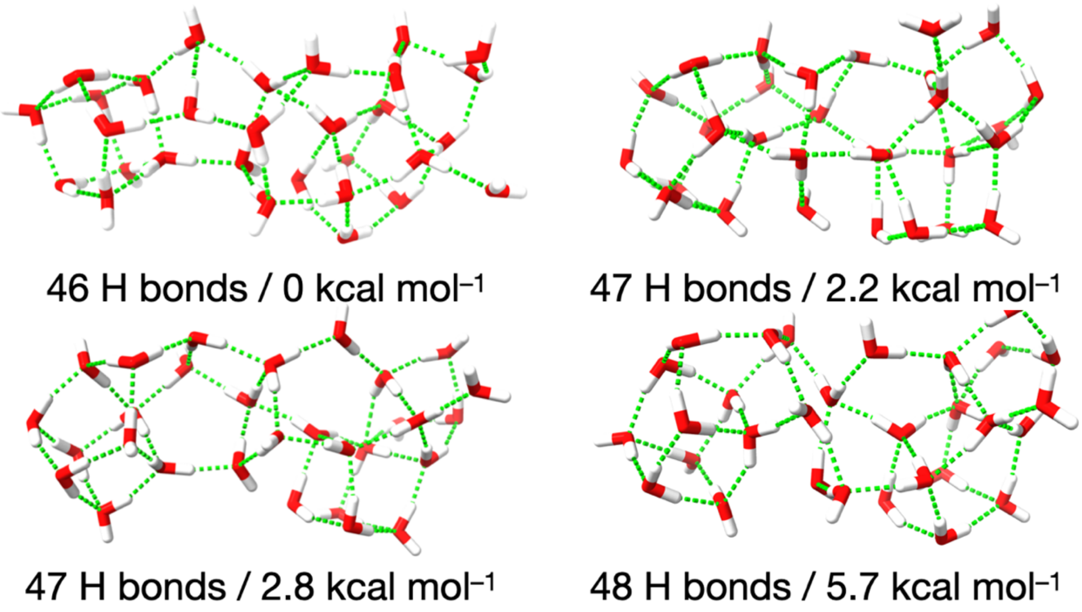

Figure S55. Hydrogen-bonding networks in low-energy $(H_2O)_{30}$ conformers within 6 kcal·mol^{-1} sampled using CREST

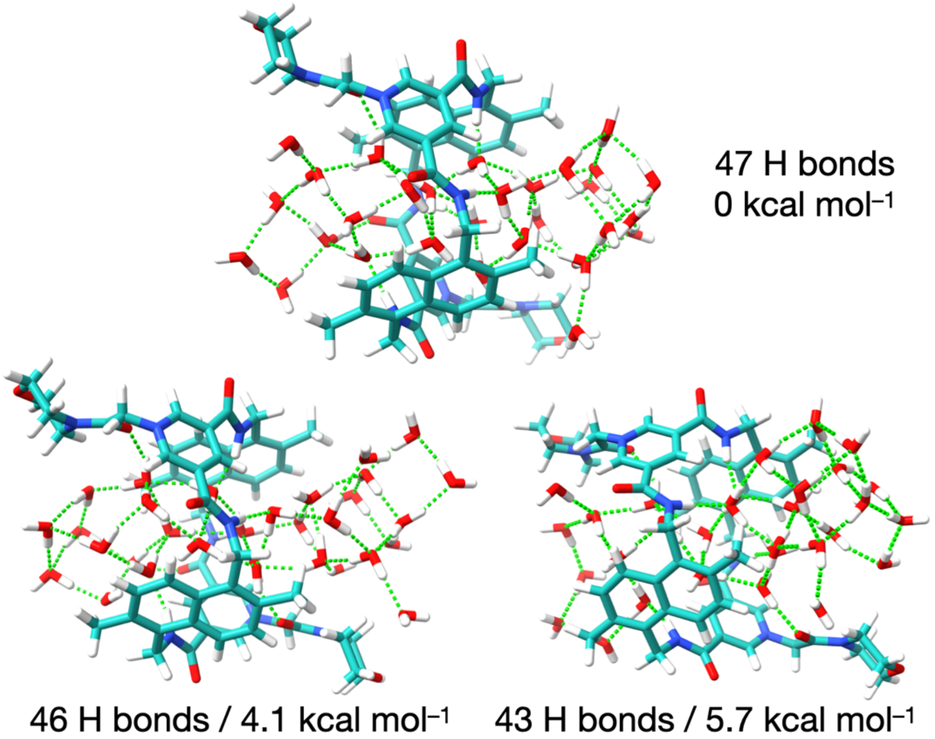

Figure S56. Hydrogen-bonding networks in low-energy $(H_2O)_{30}$ conformers in the presence of $MPNT^{2+}$ within 6 kcal·mol^{-1} sampled using CREST.

10. References

(S1) Timmer, B. J. J.; Mooibroek, T. J. A Simple Strategy to Obtain Synthetic Ca^{2+} -Dependent Lectin Mimics. *Eur J Org Chem* **2021**, *2021* (29), 4218–4223.

(S2) Collman, J. P.; Decréau, R. A.; Costanzo, S. Appending a Tris-Imidazole Ligand with a Tyr^{244} Mimic on the Distal Face of Bromoacetamidoporphyrin. *Org. Lett.* **2004**, *6* (6), 1033–1036.

(S3) Brynn Hibbert, D.; Thordarson, P. The Death of the Job Plot, Transparency, Open Science and Online Tools, Uncertainty Estimation Methods and Other Developments in Supramolecular Chemistry Data Analysis. *Chem. Commun.* **2016**, *52* (87), 12792–12805.

(S4) Thordarson, P. Determining Association Constants from Titration Experiments in Supramolecular Chemistry. *Chem. Soc. Rev.* **2011**, *40* (3), 1305–1323.

(S5) Bruker (2023). APEX4. Bruker AXS LLC, Madison, Wisconsin, USA.

(S6) Bruker SAINT. Bruker AXS LLC, Madison, Wisconsin, USA.

(S7) Krause, L.; Herbst-Irmer, R.; Sheldrick, G. M.; Stalke, D. Comparison of Silver and Molybdenum Microfocus X-Ray Sources for Single-Crystal Structure Determination. *J Appl Crystallogr* **2015**, *48* (1), 3–10.

(S8) Sheldrick, G. M. *SHELXT* – Integrated Space-Group and Crystal-Structure Determination. *Acta Crystallogr A Found Adv* **2015**, *71* (1), 3–8.

(S9) Sheldrick, G. M. Crystal Structure Refinement with *SHELXL*. *Acta Crystallogr C Struct Chem* **2015**, *71* (1), 3–8.

(S10) Dolomanov, O. V.; Bourhis, L. J.; Gildea, R. J.; Howard, J. A. K.; Puschmann, H. *OLEX2* : A Complete Structure Solution, Refinement and Analysis Program. *J Appl Crystallogr* **2009**, *42* (2), 339–341.

(S11) Bruno, I. J.; Cole, J. C.; Edgington, P. R.; Kessler, M.; Macrae, C. F.; McCabe, P.; Pearson, J.; Taylor, R. New Software for Searching the Cambridge Structural Database and Visualizing Crystal Structures. *Acta Cryst B* **2002**, *58* (3), 389–397.

(S12) Neese, F. The ORCA Program System. *WIREs Comput Mol Sci* **2012**, *2* (1), 73–78.

(S13) Becke, A. D. Density-Functional Thermochemistry. III. The Role of Exact Exchange. *The Journal of Chemical Physics* **1993**, *98* (7), 5648–5652.

(S14) Weigend, F.; Ahlrichs, R. Balanced Basis Sets of Split Valence, Triple Zeta Valence and Quadruple Zeta Valence Quality for H to Rn: Design and Assessment of Accuracy. *Phys. Chem. Chem. Phys.* **2005**, *7* (18), 3297.

(S15) Kruse, H.; Grimme, S. A Geometrical Correction for the Inter- and Intra-Molecular Basis Set Superposition Error in Hartree-Fock and Density Functional Theory Calculations for Large Systems. *The Journal of Chemical Physics* **2012**, *136* (15), 154101.

(S16) Grimme, S.; Antony, J.; Ehrlich, S.; Krieg, H. A Consistent and Accurate *Ab Initio* Parametrization of Density Functional Dispersion Correction (DFT-D) for the 94 Elements H-Pu. *The Journal of Chemical Physics* **2010**, *132* (15), 154104.

(S17) Weigend, F. Accurate Coulomb-Fitting Basis Sets for H to Rn. *Phys. Chem. Chem. Phys.* **2006**, *8* (9), 1057.

(S18) Izsák, R.; Neese, F. An Overlap Fitted Chain of Spheres Exchange Method. *The Journal of Chemical Physics* **2011**, *135* (14), 144105.

(S19) Stoychev, G. L.; Auer, A. A.; Neese, F. Automatic Generation of Auxiliary Basis Sets. *J. Chem. Theory Comput.* **2017**, *13* (2), 554–562.

(S20) Lu, T.; Chen, F. Multiwfn: A Multifunctional Wavefunction Analyzer. *J Comput Chem* **2012**, *33* (5), 580–592.

(S21) Zhang, Y.; Zhao, J. A Density Fitting Scheme for the Fast Evaluation of Molecular Electrostatic Potential. *J. Comput. Chem.* **2023**, *44* (7), 806–813.
(S22) Zhang, J.; Lu, T. Efficient Evaluation of Electrostatic Potential with Computerized Optimized Code. *Phys. Chem. Chem. Phys.* **2021**, *23* (36), 20323–20328.
(S23) Pettersen, E. F.; Goddard, T. D.; Huang, C. C.; Couch, G. S.; Greenblatt, D. M.; Meng, E. C.; Ferrin, T. E. UCSF Chimera—A Visualization System for Exploratory Research and Analysis. *J Comput Chem* **2004**, *25* (13), 1605–1612.
(S24) Lefebvre, C.; Rubez, G.; Khartabil, H.; Boisson, J.-C.; Contreras-García, J.; Hénon, E. Accurately Extracting the Signature of Intermolecular Interactions Present in the NCI Plot of the Reduced Density Gradient versus Electron Density. *Phys. Chem. Chem. Phys.* **2017**, *19* (27), 17928–17936.
(S25) Lu, T.; Chen, Q. Independent Gradient Model Based on Hirshfeld Partition: A New Method for Visual Study of Interactions in Chemical Systems. *J. Comput. Chem.* **2022**, *43* (8), 539–555.
(S26) Šivickytė, O.; Costa, P. J. Intrinsic Bond Strength Index as a Halogen Bond Interaction Energy Predictor. *Phys. Chem. Chem. Phys.* **2023**, *25* (26), 17535–17546.
(S27) Klein, J.; Khartabil, H.; Boisson, J.-C.; Contreras-García, J.; Piquemal, J.-P.; Hénon, E. New Way for Probing Bond Strength. *J. Phys. Chem. A* **2020**, *124* (9), 1850–1860.
(S28) Bannwarth, C.; Ehlert, S.; Grimme, S. GFN2-xTB—An Accurate and Broadly Parametrized Self-Consistent Tight-Binding Quantum Chemical Method with Multipole Electrostatics and Density-Dependent Dispersion Contributions. *J. Chem. Theory Comput.* **2019**, *15* (3), 1652–1671.
(S29) Pracht, P.; Grimme, S.; Bannwarth, C.; Bohle, F.; Ehlert, S.; Feldmann, G.; Gorges, J.; Müller, M.; Neudecker, T.; Plett, C.; Spicher, S.; Steinbach, P.; Wesołowski, P. A.; Zeller, F. CREST—A Program for the Exploration of Low-Energy Molecular Chemical Space. *J. Chem. Phys* **2024**, *160* (11), 114110.
(S30) Pracht, P.; Bohle, F.; Grimme, S. Automated Exploration of the Low-Energy Chemical Space with Fast Quantum Chemical Methods. *Phys. Chem. Chem. Phys.* **2020**, *22* (14), 7169–7192.
(S31) Grimme, S. Exploration of Chemical Compound, Conformer, and Reaction Space with Meta-Dynamics Simulations Based on Tight-Binding Quantum Chemical Calculations. *J. Chem. Theory Comput.* **2019**, *15* (5), 2847–2862.
(S32) Biedermann, F.; Nau, W. M.; Schneider, H. J. The Hydrophobic Effect Revisited - Studies with Supramolecular Complexes Imply High-Energy Water as a Noncovalent Driving Force. *Angew. Chem. Int. Ed.* **2014**, *53* (42), 11158–11171.
